# Immunosuppressive Cytochalasins from the Mangrove Endophytic Fungus *Phomopsis asparagi* DHS-48

**DOI:** 10.3390/md20080526

**Published:** 2022-08-18

**Authors:** Zhao Feng, Xuexia Zhang, Jingwan Wu, Chengwen Wei, Ting Feng, Dongdong Zhou, Zhenchang Wen, Jing Xu

**Affiliations:** School of Chemical Engineering and Technology, Hainan University, Haikou 570228, China

**Keywords:** mangrove endophytic fungi, *Phomopsis* sp., cytochalasins, immunosuppressive activity, CaN/NFAT signaling pathway

## Abstract

Three new cytochalasins, phomoparagins A-C (**1**–**3**), along with five known analogs (**4**–**8**), were isolated from *Phomopsis asparagi* DHS-48, a mangrove-derived endophytic fungus. Their structures, including their absolute configurations, were elucidated using a combination of detailed HRESIMS, NMR, and ECD techniques. Notably, **1** possessed an unprecedented 5/6/5/8/5-fused pentacyclic skeleton. These compounds were tested for their inhibitory activity against concanavalin A (ConA)/lipopolysaccharide (LPS)-induced spleen lymphocyte proliferation and calcineurin (CN) enzyme. Several metabolites (**2** and **4**–**6**) exhibited fascinating inhibitory activities with a relatively low toxicity. Furthermore, **2** was demonstrated to inhibit ConA-stimulated activation of NFAT1 dephosphorylation and block NFAT1 translocation in vitro, subsequently inhibiting the transcription of interleukin-2 (IL-2). Our results provide evidence that **2** may, at least partially, suppress the activation of spleen lymphocytes via the CN/NFAT signaling pathway, highlighting that it could serve as an effective immunosuppressant that is noncytotoxic and natural.

## 1. Introduction

Immunosuppressants are the main clinical drugs for the treatment of undesirable or abnormal activations in the body, such as immune system activation, which is associated with a variety of autoimmune diseases and allergic reactions, including rheumatoid arthritis, multiple sclerosis, systemic lupus erythematosus, and glomerulonephritis, and responses in organ transplantation recipients [1]. Most of the representative immunosuppressive drugs used in the clinic were developed from microbial secondary metabolites, such as cyclosporin A (CsA), tacrolimus (FK506), and rapamycin [2]. These drugs form complexes with intracellular immunophilin receptors. Among them, the CsA/cyclophilin and FK506/FKBP complexes share the same pharmacodynamic property of suppressing activated T cells by inhibiting the activity of calcineurin (CN) phosphatase; thus, these complexes prevent the dephosphorylation and nuclear translocation of activated T cell nuclear factor (NFAT) and NFAT-mediated transcription of a large number of cytokine genes, such as interleukin 2 (IL-2) [3,4]. However, despite their undeniable satisfactory therapeutic effects, the aforementioned immunosuppressive drugs have been found to cause dramatic side effects, such as nephrotoxicity, hepatotoxicity, neurotoxicity, malignancy, and other adverse effects [5,6]. Thus, the discovery of new clinically applied immunosuppressants with a high efficacy but no major cytotoxicity is urgently needed.

**Figure 1:**
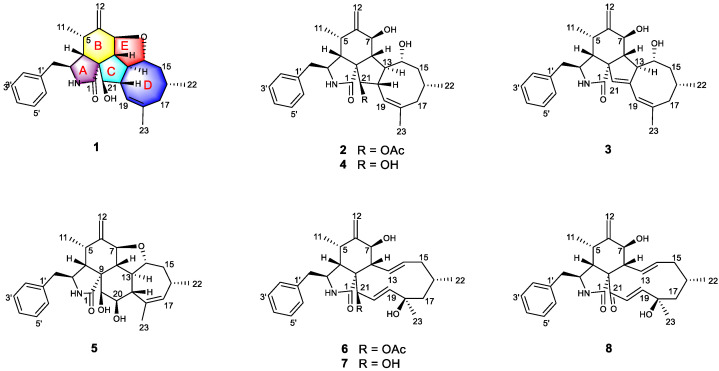
Structures of the isolated Compounds **1**–**8**.

Endophytic fungi isolated from mangrove trees are one of the most pivotal and promising sources of bioactive natural products, presumably owing to their intriguing structural skeleton and the promising pharmacological effect of their secondary metabolites, making them attractive repositories for therapeutic agents and lead compounds [7,8]. Over 50% of mangrove-derived endophytic fungal bioactive secondary metabolites are produced by the genera *Aspergillus* and *Penicillium*, while *Pestalotiopsis*, *Alternaria,* and *Phomopsis* are considered the predominant producers [9]. The *Phomopsis* (teleomorph *Diaporthe*) fungi, which contains more than 900 species named from a wide range of hosts, have attracted considerable attention from natural product researchers in recent years [10,11]. Versatile bioactive metabolites, such as cytotoxic phomopchalasins B and C [12]; cytochalasins J_1_–J_3_, H_1_, and H_2_ [13]; dicerandrols [14]; antibiotic isopentylated diphenyl ethers [15], phomoxanthone A [16] and phompsichalasin [17]; *β*-site amyloid precursor protein cleaving enzyme 1 (BACE1) inhibitory protoilludane-type sesquiterpenoids [18]; and anti-Tobacco mosaic virus (TMV) cytosporone U [19] and arylbenzofurans [20] have been isolated from *Phomopsis* strains. Among them, cytochalasins are a diverse group of polyketide synthase nonribosomal peptide synthetase (PKS-NRPS)-derived fungal metabolites characterized by a perhydroisoindolone moiety, which is typically fused to a macrocyclic ring (ring size 9–14) [21]. Since the first representatives, cytochalasin A and B, were isolated in 1966 [22], the number of natural products belonging to this family has increased to over 200 [12]. As part of our research on mangrove-derived fungi, a series of structurally novel and biologically active metabolites have been discovered [23,24,25,26,27]. Our primary application of in vitro immunosuppressive activity screening indicated that the MeOH extracts from the endophytic fungus strain *Phomopsis asparagi* DHS-48, which were obtained from a fresh root of the mangrove plant *Rhizophora mangle*, strongly inhibited ConA/LPS-induced spleen lymphocyte proliferation and CN enzyme activity, with IC_50_ values of 6.20 ± 0.20 μg/mL, 10.28 ± 0.24 μg/mL, and 78.03 ± 0.45 μg/mL, respectively. Bioassay-guided investigation of the immunosuppressive constituents obtained from the large-scale fermentation of the abovementioned *P. asparagi* DHS-48 resulted in the isolation of three new cytochalasins, namely, phomoparagins A-C (**1**–**3**), along with five known analogs, phomopchalasin A and B (**4**,**5**) [12], cytochalasin H (**6**) [28], and J (**7**) [29] and fragiformin B (**8**) [30] (Figure 1). Herein, we report the isolation, structure elucidation, and immunosuppressive activity, as well as the plausible biosynthetic pathway, of the isolated compounds.

## 2. Results

**Figure 2:**
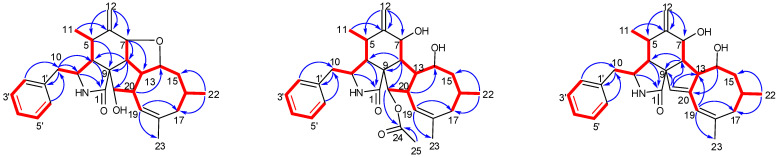
Key COSY and HMBC correlations of Compounds **1**–**3**.

Phomoparagin A (**1**) was obtained as a colorless amorphous powder. Its molecular formula, C_28_H_35_NO_3_ with 12 degrees of unsaturation, was established using the high-resolution-electrospray ionization mass spectrometry (HRESIMS) positive ion at *m*/*z* 434.2644 ([M+H]^+^, calcd for 434.2695). The ^1^H NMR data of **1** (Table 1), as well as the coupling constants of the connected protons, indicated the presence of a tertiary methyl at *δ*_H_ (1.74, 3H, s, H_3_-23), two secondary methyl groups at *δ*_H_ (0.73, 3H, d, *J* = 6.1 Hz, H_3_-11; 0.95, 3H, d, *J* = 6.7 Hz, H_3_-22), an exocyclic methylene group at *δ*_H_ (5.13 and 4.96, 2H, both s, H_2_-12), three oxygenated methine groups at *δ*_H_ (4.13, 1H, d, *J* = 9.8 Hz, H-7; 4.19, 1H, t, *J* = 9.7 Hz, H-14; 2.99, 1H, d, *J* = 2.4 Hz, H-21), an olefinic methine group at *δ*_H_ (5.28, 1H, br s, H-19), and typical resonance of a single substituted phenyl at *δ*_H_ (7.22–7.30, 5H). Its ^13^C NMR spectrum (Table 2) disclosed 28 carbon resonances, including three sp^3^ methyls, three sp^3^ methylenes, 10 sp^3^ methines, one sp^3^ quaternary carbon, one sp^2^ exocyclic methylene, six sp^2^ olefinic methines, and four sp^2^ quaternary carbons (three olefinic carbons and one amide carbonyl), as supported by the DEPT and HSQC spectra. The complete structure of **1** was established by extensive analysis of its 2D NMR spectra. The ^1^H-^1^H COSY (Figure 2) and HSQC spectra suggested the presence of the fragments CH_2_(10)-CH(3)-CH(4)-CH(5)-CH_3_(11)-, CH(7)-CH(8)-CH(13)-CH(14)-CH_2_(15)-CH(16)-CH_2_(17), incorporating CH_3_-(22), which was coupled to CH-(16); -CH(7)-CH(8)-CH(13)-CH(20)-CH(21)-, incorporating CH-(19), which was coupled to CH-(20), and CH(2′) to CH(6′). In the HMBC spectrum (Figure 2), ^13^C-^1^H long-range correlations were observed from H-3 (*δ*_H_ 3.30, m) and H-4 (*δ*_H_ 2.64, m) to C-1 (*δ*_C_ 171.7); H-4 and H-5 (*δ*_H_ 2.65, m) to C-9 (*δ*_C_ 53.8); H-7 (*δ*_H_ 4.13, d, 9.8) to C-5(*δ*_C_ 33.4), C-6(*δ*_C_ 150.9), C-12(*δ*_C_ 113.4), C-14(*δ*_C_ 76.8), and C-8(*δ*_C_ 41.9), establishing the phenylalanine moiety (rings A and B). HMBC correlations from H-8 (*δ*_H_ 2.19, t, 10.0 Hz) to C-1, C-4 (*δ*_C_ 47.9), C-9 (*δ*_C_ 53.8), and H-4 (*δ*_H_ 2.49, m) to C-21 (*δ*_C_ 75.6) supported that the five-membered ring C was fused to ring B via C-8 and C-9. An eight-membered ring D was elucidated using the HMBC correlations from H-19 (*δ*_H_ 5.28, br s) to C-17 (*δ*_C_ 43.4), C-21 (*δ*_C_ 75.6), C-18 (*δ*_C_ 138.9), and C-23 (*δ*_C_ 27.7), and from H-22 (*δ*_H_ 0.95, d, 6.7) to C-15 (*δ*_C_ 44.4) and C-17 (*δ*_C_ 43.4). Additionally, H-10 (*δ*_H_ 2.76, 2.69) to C-3 (*δ*_C_ 54.5), C-4 (*δ*_C_ 47.6), C-1′ (*δ*_C_ 138.7), and C-2′/C-6′ (*δ*_C_ 131.1) revealed the connection of the phenyl to C-3 via C-10. Comparison of the ^1^H and ^13^C NMR spectra of **1** with those of phomopchalasin A (**4**), which was previously isolated from the endophytic fungus *Phomopsis* sp. shj2 associated with *Isodon eriocalyx* var. laxif lora. [12], indicated that these two compounds possessed the same 5/6/5/8-fused tetracyclic cytochalasan ring system (rings A-D). These two compounds are different, in that a new five-membered epoxy unit (ring E) was formed by the dehydration reaction between 7-OH and 14-OH. To confirm this observation, evidence was obtained from the chemical shifts of C-8 (*δ*_C_ 41.9) and C-13 (*δ*_C_ 42.5) in **1**, which were significantly shifted upfield compared with C-8 (*δ*_C_ 51.3) and C-13 (*δ*_C_ 51.7) in **4**, along with the HRESIMS data. Thus, the connection from C-7 to C-14 via an oxygen atom was deduced from the above evidence.

**Figure 3:**
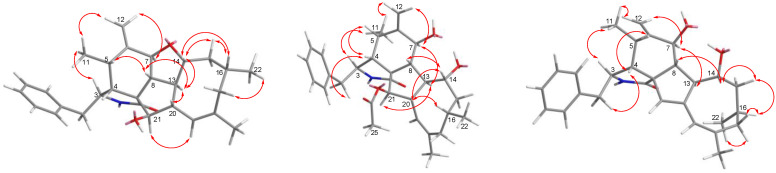
Key NOESY correlations of Compounds **1**–**3**.

The relative configuration of **1** was subsequently established by analyzing the NOESY spectrum. The NOE crosspeaks (Figure 3) between H-8/H-14, H-14/H-20, and H-20/H-16 indicated that these protons have *β* orientations. H-7/H-13 and H-13/H-21 were observed, suggesting that these protons are cofacial and have α-orientations. Considering the above evidence, we assumed that the stereochemistry is the same as that reported for **4,** based on the similarity in the 2D NMR resonances and the shared biogenetic origin.

**Figure 4:**
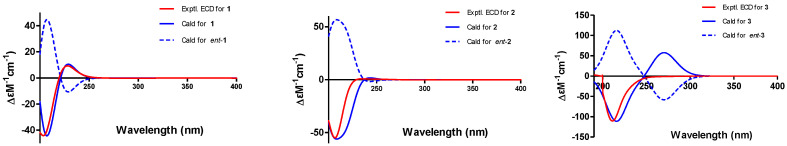
Experimental and calculated electronic circular dichroism (ECD) spectra of **1**–**3**.

The absolute configuration of **1** was determined by comparing experimental and calculated ECD spectra using time-dependent density-functional theory (TDDFT). Two feasible configurations, 3*S*, 4*R*, 5*S*, 7*S*, 8*R*, 9*R*, 13*R*, 14*R*, 16*R*, 20*S*, 21*R* and 3*R*, 4*S*, 5*R*, 7*R*, 8*S*, 9*S*, 13*S*, 14*S*, 16*S*, 20*R*, 21*S* (**1** and *ent*-**1**, respectively), were calculated at the B3LYP/6-31+G(d,p) level with a PCM solvent model for MeOH. The calculated ECD spectrum of **1** showed an excellent fit with the experimental spectrum (Figure 4), which indicated the absolute configuration to 3*S*, 4*R*, 5*S*, 7*S*, 8*R*, 9*R*, 13*R*, 14*R*, 16*R*, 20*S*, 21*R*. Thus, the complete structure of **1** was established.

Phomoparagin B (**2**) was obtained as colorless needles and has a molecular formula of C_30_H_39_NO_5_ based on HRESIMS (*m/z* 516.2727, calcd for [M+Na]^+^ 516.2726), implying 12 degrees of unsaturation. The ^1^H and ^13^C NMR spectra (Table 1 and 2) of **2** were similar to those of phomopchalasin A (**4**) [12], except that the hydroxyl group at C-21 of the latter was replaced by the acetoxy group of **2**. This was confirmed by the molecular weight difference of 42 amu observed between compounds **2** and **4**, along with the strong HMBC correlation (Figure 2) from the protons of the methyl ester group (*δ* 1.99, 3H, s) and H-21 (*δ* 3.71, 1H, *d*, 5.0) to C-24 (*δ* 173.1), which supports the presence of an acetoxy group at C-21 in **2**. The relative configuration of **2** was determined by interpreting the NOESY data (Figure 3). As expected, the experimental ECD spectrum (Figure 4) of **2** matched exactly with the calculated spectrum. Accordingly, the absolute configuration of **2** was determined to be 3*S*, 4*R*, 5*S*, 7*S*, 8*R*, 9*R*, 13*R*, 14*R*, 16*R*, 20*S*, 21*R,* and it was named phomoparagin B.

Phomoparagin C (**3**), a colorless amorphous powder, has a molecular formula of C_28_H_35_NO_3_, as established by HRESIMS (*m/z* 434.2678, calcd for [M+H]^+^ 434.2695), corresponding to 12 degrees of unsaturation. The ^1^H and ^13^C NMR data (Table 1 and 2) of **3** were similar to those of **2,** except for the signals of the -OCOCH_3_ substituent and an aliphatic methine proton within **2,** which were absent in **3**. Instead, signals of an olefinic double bond at (*δ*_C_ 137.9, s, C-20; *δ*_H_ 5.53, 1H, s; *δ*_C_ 120.1, d, CH-21) appeared, which accounts for the molecular weight difference of 60 amu that was observed between the compounds. Conformational evidence was obtained from the HMBC correlations of H-21 to C-8(*δ*_C_ 38.2)/C-9(*δ*_C_ 52.7)/C-13(*δ*_C_ 47.5) (Figure 2). The relative stereochemistry of compound **3** was determined using the key NOSEY cross peaks (Figure 3) of H-3 with H_3_-11; H-8 with H-4 and H-20; and H-14 with H-16 and H-20. The absolute configuration could be determined as 3*S*, 4*R*, 5*S*, 7*S*, 8*R*, 9*R*, 13*R*, 14*R*, 16*R,* by comparing the experimental and calculated ECD spectra using TDDFT (Figure 4). Thus, the structure of **3** was determined, and it was named phomoparagin C.

**Figure 5:**
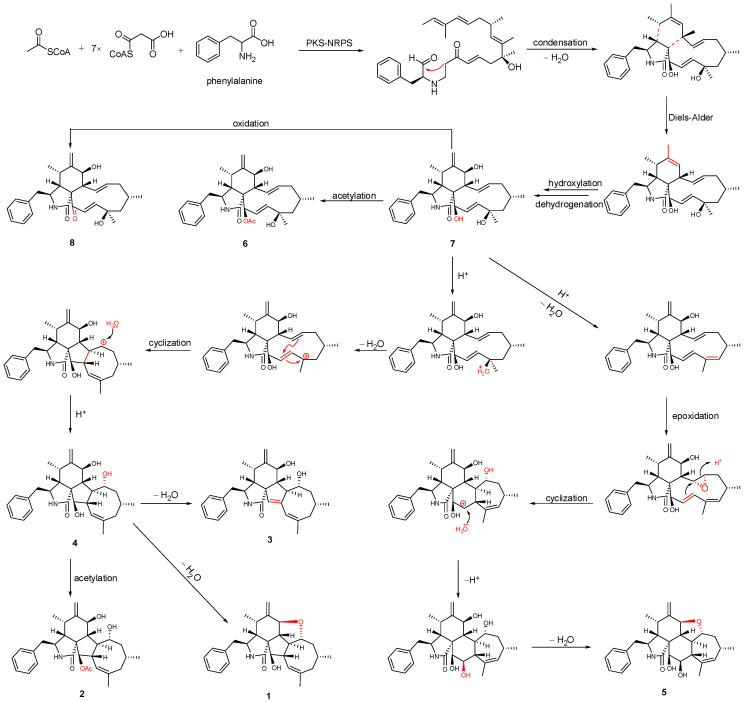
Plausible biogenetic relationship of isolated compounds.

Previous isotope labeling experiments revealed that cyctochalasans might rationally share the same biosynthetic pathway, and it most likely originates from a polyketide synthase (PKS)/nonribosomal peptide synthetase (NRPS) hybrid machinery [31,32]. The stepwise assembly is realized from one activated acetyl-CoA starter, seven malonyl-CoA extender units and phenylalanine. An intramolecular aldol condensation generates pyrrolinone, which reacts via a [4 + 2]-cycloaddition, hydroxylation, and dehydrogenation to generate the same biosynthetic precursor **7**. Subsequent acetylation and oxidation of 7 formed **6** and **8**, respectively. Compound **5**, which contains a 5/6/6/7/5-fused pentacyclic ring system, might originate by dehydration, epoxidation, intramolecular nucleophilic addition, hydroxylation, and dehydration reactions. In another pathway, **7** undergoes dehydration, intramolecular rearrangement, and hydroxylation, to produce the 5/6/5/8-fused tetracyclic intermediate **4**. The subsequent intramolecular dehydration and acetylation led to the formation of **1**–**3** (Figure 5). Of these, **1** possesses an unprecedented 5/6/5/8/5-fused pentacyclic ring system.

The isolated compounds (**1**–**8**) were evaluated for their immunosuppressive activities against the proliferation of ConA-induced T and LPS-induced B murine splenic lymphocytes, according to previously described protocols [33,34,35]. The results showed that Compounds **2** and **4**–**6** remarkably inhibited the proliferation against splenic lymphocyte growth, with IC_50_ values ranging from 11.2 ± 0.3 μM to 154.4 ± 0.4 μM, of which **2** and **6** displayed the most promising inhibitory effects (Table 3). The cytotoxicity of immunosuppressive Compounds **2** and **4**–**6** was tested in murine splenocyte cultures for 72 h using the tetrazolium salt-based CCK-8 assay. The results (Table 4) showed that even **6** exhibited better suppression of the overproduction of the cell stimulated by ConA compared with that of **2**, but **2** exhibited relatively lower toxicity for the survival of normal splenocytes (IC_50_ = 111.7 ± 1.1 μM) than that of **6** (IC_50_ = 42.2 ± 1.7 μM) and it was approximately 11-fold lower in comparison with that of CsA (IC_50_ = 10.9 ± 0.8 μM) and cytochalasin D (IC_50_ = 1.0 ± 0.0 μM), indicating that the compound has a relatively low toxicity toward the survival of normal splenic cells. Thus, we selected this particular cytochalasin for the mechanism of action studies.

**Figure 6:**
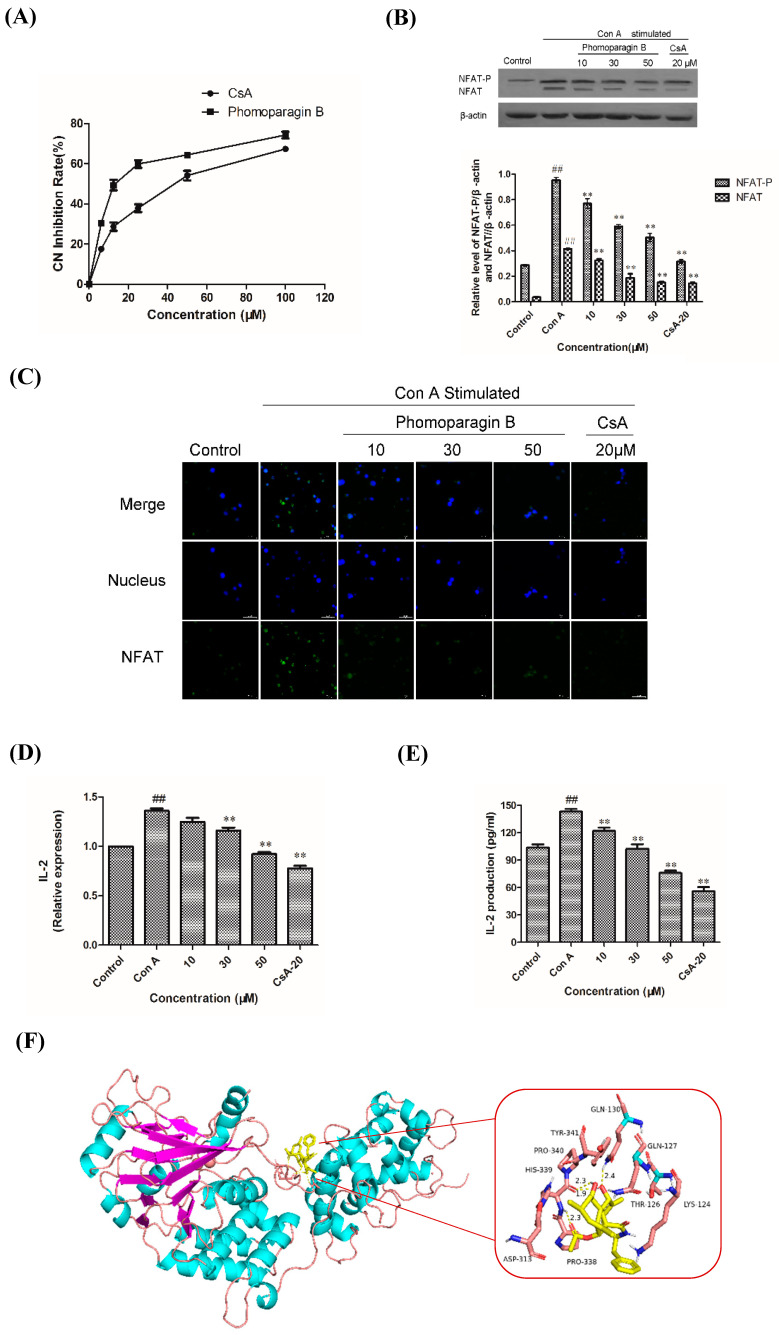
The effect of **2** on calcineurin activity (**A**). Effects of **2** on the expression of ConA-induced NFAT1 protein and analyzed by Western blot (**B**). Effects of **2** on the expression of ConA-induced NFAT protein and analyzed by Western blot (**C**). Effect of **2** in ConA-induced mouse T lymphocytes on IL-2 mRNA expression by q-PCR (**D**) and IL-2 secretion by ELISA (**E**). Molecular docking analysis of the binding of **2** to calcineurin (**F**). ** *p* < 0.01 compared to the stimulated group. ## *p* < 0.01 compared to the control group.

**Figure 7:**
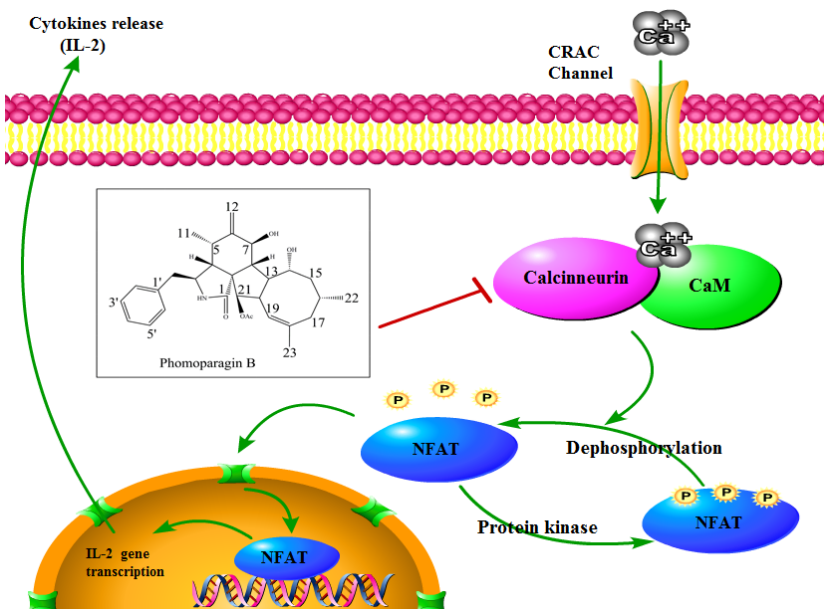
Schematic view of **2** acting on the CN/NFAT signaling pathway.

To directly examine whether **2** specifically inhibits the CaN/NFAT pathway, we first investigated the CaN inhibition rate of **2**. As expected, **2** was found to be significantly active and inhibited CN in a dose-dependent manner with an IC_50_ value of 17.89 ± 0.40 μM, which has a greater potency than that of the clinically used immunosuppressant cyclosporine A (CsA, IC_50_ of 31.7 ± 0.7 μM) (Figure 6A). The effect of **2** on ConA-stimulated NFAT1 and NFAT-P expression was determined by Western blotting. In unstimulated cells, NFAT was found exclusively in the phosphorylated form, reflecting that calcineurin is inactive under resting conditions. In contrast, in the ConA-stimulated cells in the absence of **2,** the dephosphorylated form of NFAT1 was detected, as well as the phosphorylated form. The presence of 50 μM **2** strongly inhibited the dephosphorylation of NFAT (Figure 6B). Correspondingly, immunofluorescence analysis demonstrated that NFAT1 protein was diffusely distributed in the cytoplasm and was absent from the nucleus. After 48 h of stimulation with 5 μg/mL Con A, the fluorescent NFAT1 translocated to the nucleus. In contrast, the presence of **2** blocked the Con A-stimulated translocation of NFAT1 from the cytoplasm to the nucleus in a dose-dependent manner (Figure 6C). With respect to the effect of **2** on the expression levels of IL-2 mRNA, as determined by real-time quantitative PCR (q-PCR), the transcription level of Con A-stimulated IL-2 mRNA decreased with increasing concentrations of **2** (Figure 6D). ELISA experiments further confirmed the effects of **2** at the IL-2 protein level (Figure 6E). Molecular docking was then performed to further understand the possible binding modes and binding affinities of highly active **2** with the active sites of CN using AutoDock 4.2. As shown in Figure 6F, **2** formed four key hydrogen bonds with residues GLN-130 and HIS-339 with a binding energy of -5.92 kcal·mol^−1^. In addition, **2** formed hydrophobic interactions with residues ILE-436, ILE-474, ARG-477, VAL-490, and VAL-498 and intermolecular interactions with residues THR-126, GLN-127, ASP-313, and PRO-340. Therefore, the immunosuppressive activity of **2** is possibly, at least in part, mediated via CaN/NFAT signaling pathway-regulated Con A-stimulated activation of splenocytes (Figure 7), highlighting its potential for use as an effective noncytotoxic natural immunosuppressant.

## 3. Materials and Methods

### 3.1. General Experimental Procedures

A WYA-2S digital Abbe refractometer (Shanghai Physico-optical Instrument Factory) was used to measure optical rotations. Circular dichroism (CD) spectra were measured on a JASCO J-715 spectra polarimeter. An LTQ Orbitrap XL instrument (Thermo Fisher Scientific, Bremen, Germany) was used to record HRESIMS data. 1D and 2D NMR spectra were recorded on a Bruker AV-400 spectrometer for ^1^H nuclei and 100 MHz for ^13^C nuclei in CD_3_OD. An Agilent 1100 instrument was applied for HPLC analysis and semipreparative HPLC separation. Sephadex LH-20 (18−110 µm, Merck, Darmstadt, Germany), RP-18 gel (25–40 μm, Daiso Inc., Osaka, Japan), or silica gel (200–300 mesh, Qingdao Marine Chemical Inc., Qingdao, China) were employed for column chromatography. Thin-layer chromatography was performed over F_254_ glass plates (200–400 mesh, Qingdao Marine Chemical Inc., Qingdao, China) and analyzed under UV light (254 and 366 nm). The purity of the isolated compounds was determined by high-performance liquid chromatography (HPLC), which was performed on an Agilent 1200 instrument and a reverse-phase column (4.6 × 150 mm, 5 μm). The UV wavelength for detection was 210 nm. All compounds were eluted with a flow rate of 0.7 mL·min^−1^ over a 15-min gradient, as follows: T = 0, 95% B; T = 15, 100% B (A, H_2_O; B, MeOH) and the purity of tested compounds were proven to exceed 95% (Figure S43).

### 3.2. Fungal Material

The strain DHS-8 of *Phomopsis asparagi* was isolated from a healthy tree root of the mangrove plant *Rhizophora mangle*, which was collected in the Dong Zhai Gang-Mangrove Garden (110°320′–110°37′ E, 19°51′–20°01′ N) in Hainan Province in October 2015. The strain was isolated under sterile conditions from the inner tissue of the root, following an isolation protocol described previously [36] and identified using a molecular biological protocol by DNA amplification and sequencing of the ITS region (GenBank Accession no. MT126606). A voucher strain was preserved on potato dextrose agar slants stored at 4 °C at one of the authors’ laboratory (J. X.).

### 3.3. Fermentation and Extraction

The fungus was fermented onto autoclaved rice solid-substrate medium (thirty 1000 mL Erlenmeyer flasks, each containing 100 g of rice and 100 mL of 0.3% saline water) and incubated for 28 days at 28 °C. In total, 140 flasks of culture were extracted three times with EtOAc, and the filtrate was evaporated under reduced pressure to yield crude extract (65 g).

### 3.4. Purification and Identification

The crude extract was partitioned with petroleum ether (PE), dichloromethane, ethyl acetate (EA), and *n*-butyl alcohol (BA). The dichloromethane fraction and ethyl acetate fraction were combined (30 g) and then subjected to silica gel column chromatography using gradient elution with CH_2_Cl_2_–MeOH mixtures of increasing polarity (100:0–0:100, *v*/*v*) to afford 8 fractions (Fr. 1–Fr. 8). Fr. 2 was subjected to open silica gel CC using gradient elution with CH_2_Cl_2_-EtOAc (4:1–1:1, *v*/*v*) to yield fractions Fr. 2.1–2.6. Then Fr. 2.3 and Fr. 2.4 were purified by semipreparative reversed-phase HPLC using MeOH–H_2_O (50:50, *v*/*v*) to afford **3** (5.0 mg) (Figures S19–S27). Purification of Fr. 2.5 was conducted by CC over RP-18 with a MeOH–H_2_O gradient (50:50–90:10, *v*/*v*) to yield **2** (8.0 mg) (Figures S10–S18) and **6** (119.2 mg) (Figures S34–S36). Fr. 2.6 was separated using Sephadex LH-20 CC with CHCl_3_–MeOH (1:1, *v*/*v*) to afford **1** (105 mg) (Figures S1–S9). Fr. 4 was subjected to CH_2_Cl_2_-EtOAc (1:1, *v*/*v*) and further fractionated by RP C-18 CC eluted with CH_3_OH-H_2_O (70:30, *v*/*v*) to produce the major component **7** (818.3 mg) (Figures S37–S39). Fr. 5 was subjected to silica gel CC using CH_2_Cl_2_-CH_3_OH as the eluent (100:4–100:6, *v*/*v*). The promising subfraction Fr. 5.1 was separated by semipreparative reversed-phase HPLC using MeOH–H_2_O (60:40, *v*/*v*) to obtain **8** (2.7 mg) (Figures S40–S42). Separations of Fr. 5.4 following a procedure similar to that used for Fr. 5.1 gave **4** (semiprep. HPLC, MeOH–H_2_O = 60:40, *v*/*v*, 120 mg) (Figures S28–S30). Fr. 6.2, collected from Fr. 6 was subjected to silica gel CC with CH_2_Cl_2_-EtOAc (100:10, *v*/*v*), followed by Sephadex LH-20 CC using CHCl_3_–MeOH (1:1, *v*/*v*) as the eluent to yield **5** (6.1 mg) (Figures S31–S33).

**Phomoparagin** **A** (**1**): colorless amorphous residue (MeOH); [α]^20^_D_ -13 (*c* 0.001, MeOH); UV (MeOH) λ_max_ 215 nm; ^1^H and ^13^C NMR data, see Table 1; HRESIMS *m/z* 434.2644 [M + H]^+^ (calcd for C28H36NO3, 434.2695).
**Phomoparagin** **B** (**2**): colorless amorphous residue (MeOH); [α]^20^_D_ +54 (*c* 0.001, MeOH); UV (MeOH) λ_max_ 212 nm; ^1^H NMR data, see Table 1; HRESIMS *m/z* 516.2733 [M + Na]^+^ (calcd for C_30_H_39_NO_5_Na, 516.2726).
**Phomoparagin** **C** (**3**): colorless amorphous residue (MeOH); [α]^20^_D_ -14 (*c* 0.001, MeOH); UV (MeOH) λ_max_ 212 nm; ^1^H and ^13^C NMR data, see Table 1; HRESIMS *m/z* 434.2678 [M + H]^+^ (calcd for C_28_H_36_NO_3_, 434.2695).

### 3.5. Electron Circular Dichroism Calculation

Monte Carlo conformational searches were run by employing Spartan’s 14 software using the Merck Molecular Force Field (MMFF). Conformers with a Boltzmann population of over 0.4% were chosen for ECD calculations (Tables S1 and S2). Then, the conformers were initially optimized at the B3LYP/6-31 g level in gas using the PCM polarizable conductor calculation model. The theoretical calculation of ECD was conducted in MeOH using time-dependent density functional theory (TD-DFT) at the B3LYP/6-31+g (d, p) level for all conformers of Compounds **1**–**3**. Rotatory strengths for a total of 30 excited states were calculated. ECD spectra were generated using the program SpecDis 1.6 (University of Würzburg, Würzburg, Germany) and GraphPad Prism 5 (University of California San Diego, USA) from dipole-length rotational strengths by applying Gaussian band shapes with sigma = 0.3 eV.

### 3.6. Splenocyte Proliferation Assay

Spleen cells were collected from BALB/c mice under aseptic conditions, plated in a 96-well plate at a density of 1.5 × 10^6^ cells/well and activated by Con A (5 μg/mL) or LPS (10 μg/mL) in the presence of various concentrations of compounds or cyclosporine A (CsA) at 37 °C and 5% CO_2_ for 48 h. Then, 20 μL CCK-8 was added to each well, 4 h before the end of the incubation. Absorbance at OD_450_ was measured on an ELISA reader, and the IC_50_ value was calculated from the correlation curve between the compound concentration and the OD_450_.

### 3.7. Cell Viability Assessment

Cell viability was evaluated using the CCK-8 method. Spleen cells were plated in a 96-well plate at a density of 1.5 × 10^6^ cells/well. Then, the cells were incubated with various concentrations of compounds or 0.1% DMSO at 37 °C and 5% CO_2_ for 72 h. Proliferation was measured using the CCK-8 assay, as described above. Cyclosporin A (CsA) and cytochalasin D were used as positive controls.

### 3.8. CN Phosphatase Assay

A spectrophotometric assay was used to determine the activities of CN with 20 mM *p*-nitrophenyl phosphate (*p*-NPP) as the substrate in 1 mM CaCl_2_, 0.5 mM MnCl_2_, 2 mM CaM, 2 mM CNB, 1 mM DTT, 0.1 mg/mL bovine serum albumin (BSA), and 50 mM Tris-HCl (pH 7.4) at 4 °C. The reaction was initiated by the addition of CNA, various concentrations of compounds (6.25, 12.5, 25, 50 or 100 μM) were added, and the solution was preincubated for 10 min at 4 °C. The OD_410_ value was measured, and the inhibitory concentration (IC_50_) was calculated. DMSO (2%) was used as a vehicle control. CsA was used as a positive control.

### 3.9. Western Blot Analysis

Splenocytes were washed with PBS and lysed in PMSF lysate. After centrifugation at 12,000 rpm for 5 min, the protein concentration was determined using a BSA Protein Assay Kit. Protein lysates were separated using 10% sodium dodecyl sulfate–polyacrylamide gel electrophoresis (SDS–PAGE) and transferred to polyvinylidene difluoride (PVDF) membranes. After blocking, the membranes were incubated overnight with the respective primary antibodies against NFAT1, NFAT-P, and β-actin in 5% BSA, and the secondary antibody was horseradish peroxidase (HRP)-conjugated goat anti-rabbit IgG. Immunoreactive bands were visualized by incubation with luminescent liquid and exposed to light-sensitive film.

### 3.10. Immunofluorescence

Splenocytes were seeded in a 24-well plate at a density of 5 × 10^6^ cells/well, incubated with various concentrations of Compound **2** or CsA, and then activated by culturing with Con A (5 μg/mL) for 48 h. After treatment, the cells were fixed with 4% paraformaldehyde for 30 min, permeabilized in 0.5% Triton X-100 for 15 min, blocked with 5% BSA for 1 h, and then incubated overnight in the respective primary antibody; NFAT1 (D43B1) XP^®^ Rabbit antibody was used at a 1:100 dilution in PBS. The corresponding fluorescent anti-rabbit IgG (H+L) secondary antibody was added for 1 h. Nuclear staining was performed with the addition of 300 μL DAPI for 5 min. Stained cells were washed with PBS and visualized using confocal microscopy.

### 3.11. Real-Time Quantitative PCR

Total cellular RNA was isolated from splenocytes (5 × 10^6^ cells/well) treated with the indicated concentrations of compounds or cyclosporin A for 48 h and extracted using RNA isolate. The obtained total RNA (2 µg) was used for cDNA synthesis with a Servicebio^®^RT First Strand cDNA Synthesis Kit with random primers, according to the manufacturer’s instructions. qPCR was performed ith SYBR Green qPCR Master Mix (High ROX) using ABI StepOne Plus Real-time Detection System and *β*-actin as internal control. The primers were as follows: IL-2 forward, 5′-TGTCACAAACAGTGCACCTACTTC-3′; IL-2 reverse, 5′-TGTGGCCTTCTTGGGCATGT-3′; *β*-actin forward, 5′-GTGACAGCAGTCGGTTGGAG-3′; *β*-actin reverse, 5′-AGTGGGGTGGCTTTTAGGAT-3′. The expression levels of genes were normalized to the expression of *β*-actin mRNA and analyzed using the delta-delta CT method (2^−ΔΔCT^).

### 3.12. IL-2 ELISA Assay

Splenocytes were treated as described in the RT–qPCR section, and then the IL-2 levels in the obtained culture supernatants were measured using an enzyme-linked immunosorbent assay (ELISA) kit, according to the manufacturer’s instructions. Cytokine standard curves were used to calculate the amount of IL-2, and the absorbance of each well was read at OD_450_ using an ELISA reader.

### 3.13. Molecular Docking Analysis

Molecular docking studies were performed to investigate the binding mode of Compound **2** with calcineurin using AutoDock 4.2 software. The crystallographic structure of calcineurin (PDB ID: 1AUI) was obtained from the Research Collaboratory for Structural Bioinformatics Protein Data Bank (RCSB PDB, http://www.rcsb.org, accessed on 17 December 2021). The structure file 1AUI was protonated, and water was deleted at pH 7 using the Clean Protein tool. The 3D structure of the small molecule was built using ChemBioDraw Ultra 14.0 (Cambridgesoft Corp., Cambridge, MA, USA) and optimized using MM2 and the steepest gradient method in Chem3D Ultra 14.0 (Cambridgesoft Corp., Cambridge, MA, USA). The Lamarckian genetic algorithm method was used in AutoDock 4.2 (Scripps Research, San Diego, CA, USA), and a docking site was defined as all residues within an RMS tolerance of 1.0 Å. The default parameters were used if no other parameters are mentioned. The obtained results were analyzed and visualized with PyMOL software (Schrödinger, New York, USA).

### 3.14. Statistical Analysis

All the results are presented as the mean ± SD, and the difference was considered significant at the *p* < 0.05 level. GraphPad Prism v5.0 (University of California San Diego, USA) was employed to analyze the data and draw plots. One-way analysis of variance (ANOVA) was used to determine the statistical significance of differences between means in SPSS13.0 (Chicago, IL, USA).

## 4. Conclusions

In summary, our chemical exploratory investigations on the mangrove-derived endophytic fungus *Phomopsis asparagi* DHS-48 led to the characterization of three new cytochalasins and five known analogs. Phomoparagin A (**1**) represents the first example of a cytochalasan that features an unprecedented 5/6/5/8/5-fused pentacyclic skeleton, and phomoparagin B (**2**) of the CN-NFAT signaling pathway was discovered and is a promising immunosuppressant, as it directly inhibited calcineurin and did not require a matchmaker protein, such as the clinical immunosuppressants CsA and FK506. The potent immunosuppressive activity and low toxicity of **2** collectively suggested that it is an attractive option for immunosuppressive drug development.

## Figures and Tables

**Table 1:** ^1^H NMR data (δ) for **1**–**3** in CD_3_OD (400 MHz) (δ in ppm, J in Hz).

No.	1	2	3
1			
3	3.30, m	3.43, q (5.0)	3.32, m
4	2.64, m	2.76, d (6.9)	2.67, m
5	2.65, m	2.53, m	2.70, m
6			
7	4.13, d (9.8)	4.19, d (8.3)	3.90, d (11.1)
8	2.19, t (10.0)	1.98, m	2.87, m
9			
10	2.76, dd (13.2, 5.7) 2.69, m	2.84, dd (12.6, 4.9) 2.62, m	2.83, m 2.68, m
11	0.73, d (6.1)	0.59, d (6.9)	0.65, d (6.3)
12	5.13, s 4.96, s	4.97, s 4.83, s	5.09, s 4.95, s
13	1.52, m	3.19, q (10.9)	3.19, brs
14	4.19, t (9.7)	4.65, t (9.0)	3.34, d (2.4)
15	2.36, d (13.5) 1.03, qd (13.5, 2.4)	1.87, dt (14.2, 8.7) 1.46, dd (14.2, 2.9)	2.31, dd (12.5, 6.9) 1.73, dd (12.5, 9.9)
16	1.49, m	2.13, m	1.95, m
17	1.95, m	2.58, m 1.99, m	2.86, m 1.48, dd (13.7, 3.2)
18			
19	5.28, brs	5.55, d (7.1)	5.18, d (6.0)
20	2.68, m	2.50, m	
21	2.99, d (2.4)	3.71, d (5.0)	5.53, s
22	0.95, d (6.7)	1.01, d (7.0)	0.94, d (6.8)
23	1.74, s	1.78, s	1.80, s
24			
25		1.99, s	
1′			
2′, 6′	7.22, d (7.1)	7.28, d (7.2)	7.23, d (7.2)
3′, 5′	7.30, t (7.2)	7.30, t (7.6)	7.25, d (7.2)
4′	7.24, t (6.4)	7.21, t (6.8)	7.24, d (6.4)

**Table 2:** ^13^C NMR data (δ) for **1**–**3** in CD_3_OD (100 MHz) (δ in ppm, J in Hz).

No.	1	2	3
1	179.1, C	181.9, C	179.1, C
3	54.5, CH	54.5, CH	54.0, CH
4	47.6, CH	44.4, CH	47.1, CH
5	33.4, CH	31.5, CH	33.1, CH
6	150.9, C	153.4, C	152.5, C
7	76.0, CH	74.1, CH	72.8, CH
8	41.9, CH	53.4, CH	38.2, CH
9	53.8, C	58.0, C	52.7, C
10	44.9, CH_2_	43.9, CH_2_	45.0, CH_2_
11	13.1, CH_3_	13.4, CH_3_	13.0, CH_3_
12	113.4, CH_2_	111.8, CH_2_	113.7, CH_2_
13	42.5, CH	47.7, CH	47.5, CH
14	76.8, CH	80.7, CH	73.2, CH
15	44.4, CH_2_	41.3, CH_2_	42.3, CH_2_
16	33.5, CH	32.0, CH	35.2, CH
17	43.4, CH_2_	38.9, CH_2_	37.5, CH_2_
18	138.9, C	136.0, C	138.1, C
19	128.5, CH	129.1, CH	123.2, CH
20	43.4, CH	54.4, CH	137.9, C
21	75.6, CH	83.1, CH	120.1, CH
22	25.4, CH_3_	24.8, CH_3_	22.1, CH_3_
23	27.7, CH_3_	27.2, CH_3_	28.2, CH_3_
24		173.1, C	
25		21.9, CH_3_	
1′	138.7, C	139.4, C	138.9, C
2′,6′	131.1, CH	130.8, CH	131.0, CH
3′,5′	129.5, CH	129.4, CH	129.5, CH
4′	127.7, CH	127.5, CH	127.7, CH

**Table 3:** Immunosuppressive activities of isolated compounds ^a^.

Compound	IC_50_ (μM) ^b^	
	ConA-Induced T-Cell Proliferation	LPS-Induced B-Cell Proliferation
2	21.6 ± 1.7	78.5 ± 1.3
4	32.8 ± 2.4	144.9 ± 2.2
5	31.2 ± 2.5	154.4 ± 0.4
6	11.2 ± 0.3	102.8 ± 1.1
cyclosporin A ^c^	4.4 ± 0.0	25.1 ± 0.4

^a^ Compound **1**, **3**, **7**–**8** were inactive (IC_50_ > 200 μM). ^b^ Data are presented as mean ± SD from three separate experiments. ^c^ Positive control.

**Table 4:** Cytotoxicity data of immunosuppressive compounds ^a^.

Cell Line	Compound					
	2	4	5	6	CsA	Cytochalasin D
murine splenocytes	111.7 ± 1.1	373.7 ± 3.3	84.4 ± 0.3	42.17 ± 1.7	10.9 ± 0.8	1.0 ± 0.0

^a^ Results are expressed as IC_50_ values of mean ± SD (*n* = 7) in μM.

## Supplementary Materials

The following supporting information can be downloaded at: https://www.mdpi.com/article/10.3390/md20080526/s1.
