# Supplementary material for: Immunosuppressive Cytochalasins from the Mangrove Endophytic Fungus Phomopsis asparagi DHS-48
Source: Mar Drugs. 2022 Aug 18;20(8):526. doi: 10.3390/md20080526 (PMC9409992; doi:10.3390/md20080526)
Supplement: Supplementary file 1 [file marinedrugs-20-00526-s001.zip › marinedrugs-1833593-SI.pdf]

Electronic Supplementary Information

Immunosuppressive Cytochalasins from the Mangrove Endophytic Fungus *Phomopsis asparagi* DHS-48

Zhao Feng, [†] Xuexia Zhang, [†] Jingwan Wu, Chengwen Wei, Ting Feng, Dongdong Zhou, Zhenchang Wen, Jing Xu[*]

School of Chemical Engineering and Technology, Hainan University,

Haikou 570228, China.

† These authors have contributed equally to this work.

[*] To whom correspondence should be addressed.

Jing Xu, Tel.: ++86–898–6627–9226, Fax: ++86–898–6627–9010, E–mail: *happyjing3@163.com*

1. Supporting tables and Figures

Table S1. Gibbs free energies^a and equilibrium populations^b of low-energy conformers of phomoparagin A (**1**)

Conformers	In MeOH	
	G^a	P (%)^b
**1-1**	-858394.65264306	2.27
**1-2**	-858396.16243212	29.12
**1-3**	-858394.17071538	1.01
**1-4**	-858396.64059474	65.32
**1-5**	-858394.65264306	2.27

^{a}B3LYP/6-31G(d,p), in kcal/mol. ^{b}From G values at 298.15K.

Table S2. Cartesian coordinates for the low-energy reoptimized MMFF conformers of phomoparagin A (**1**) at B3LYP/6-31G(d,p) level of theory in gas

Conformer **1-1**

**1-1**			Standard Orientation (Ångstroms)		
Center number	Atomic number	Atomic Type	X	Y	Z
1.	6.	0.	-1.031434	2.851506	0.200449
2.	6.	0.	-1.343497	1.348961	0.700998
3.	6.	0.	-0.287170	0.340826	0.168161
4.	6.	0.	1.028295	1.036982	0.215486
5.	6.	0.	1.348774	2.309720	-0.485917
6.	6.	0.	0.454333	3.295677	0.236665
7.	6.	0.	-2.681416	0.801336	0.142090
8.	7.	0.	-2.230084	0.147541	-1.087424
9.	6.	0.	-0.884572	-0.084423	-1.178749
10.	6.	0.	0.147734	-1.002428	0.928013
11.	6.	0.	1.746298	-1.069016	0.857540
12.	6.	0.	2.128473	0.095722	-0.076519
13.	6.	0.	-3.417752	-0.157486	1.110651
14.	6.	0.	-4.650069	-0.780823	0.493183
15.	6.	0.	-5.886672	-0.120639	0.536602
16.	6.	0.	-7.017173	-0.671291	-0.067149
17.	6.	0.	-6.930539	-1.897119	-0.729591
18.	6.	0.	-5.707280	-2.566696	-0.780481
19.	6.	0.	-4.578930	-2.012563	-0.173465
20.	6.	0.	0.886546	4.376859	0.883964
21.	6.	0.	-1.576625	3.203985	-1.208667
22.	8.	0.	-0.305028	-0.600318	-2.124804
23.	8.	0.	-0.324661	-0.934121	2.269029
24.	1.	0.	1.138579	1.291557	1.284740
25.	1.	0.	-1.350100	1.358004	1.793433

26.	6.	0.	2.381052	-2.363168	0.411210
27.	6.	0.	3.711771	-2.539098	0.328129
28.	6.	0.	4.706360	-1.511962	0.862352
29.	6.	0.	5.412217	-0.458885	-0.060575
30.	6.	0.	4.537918	0.653011	-0.713087
31.	6.	0.	3.314435	1.052113	0.099923
32.	8.	0.	2.789429	2.370796	-0.332094
33.	6.	0.	6.296826	-1.107713	-1.133918
34.	6.	0.	4.286196	-3.833967	-0.195486
35.	1.	0.	2.075095	-0.813220	1.876096
36.	1.	0.	2.078505	-0.247416	-1.115159
37.	1.	0.	-3.376638	1.609027	-0.109935
38.	1.	0.	-1.564491	3.498170	0.907950
39.	1.	0.	1.113164	2.275706	-1.558928
40.	1.	0.	-2.858122	-0.169783	-1.812160
41.	1.	0.	-0.285010	-1.875143	0.418909
42.	1.	0.	-2.719944	-0.926875	1.454326
43.	1.	0.	-3.701133	0.427211	1.994371
44.	1.	0.	-5.964694	0.831420	1.056784
45.	1.	0.	-7.966587	-0.145780	-0.016260
46.	1.	0.	-7.810276	-2.329023	-1.197505
47.	1.	0.	-5.630955	-3.523937	-1.288384
48.	1.	0.	-3.631275	-2.543401	-0.210161
49.	1.	0.	0.215812	5.006078	1.462967
50.	1.	0.	1.935179	4.654918	0.853493
51.	1.	0.	-1.359103	4.256193	-1.412699
52.	1.	0.	-1.117554	2.611782	-2.003704
53.	1.	0.	-2.659069	3.074057	-1.283607
54.	1.	0.	-0.026023	-1.729218	2.730962
55.	1.	0.	1.721505	-3.156676	0.060199
56.	1.	0.	4.233625	-0.961461	1.682623
57.	1.	0.	5.526783	-2.074996	1.328779
58.	1.	0.	6.084403	0.059169	0.638654
59.	1.	0.	5.172933	1.531896	-0.871894
60.	1.	0.	4.200004	0.333732	-1.706722
61.	1.	0.	3.578090	1.152189	1.163365
62.	1.	0.	6.885429	-0.348648	-1.660780
63.	1.	0.	5.692673	-1.628887	-1.884178
64.	1.	0.	6.996212	-1.830049	-0.699131
65.	1.	0.	3.499009	-4.530210	-0.498385
66.	1.	0.	4.938435	-3.668367	-1.060630
67.	1.	0.	4.903613	-4.328178	0.566626

Conformer **1-2**

**1-2**			Standard Orientation (Ångstroms)		
Center number	Atom number	Type	X	Y	Z
1.	6.	0.	-0.996105	2.879993	0.165026
2.	6.	0.	-1.316512	1.390647	0.692238
3.	6.	0.	-0.250920	0.372488	0.199227
4.	6.	0.	1.067283	1.063004	0.253362
5.	6.	0.	1.404815	2.332547	-0.445759
6.	6.	0.	0.486270	3.324431	0.238583
7.	6.	0.	-2.645020	0.831992	0.120565
8.	7.	0.	-2.172011	0.148340	-1.084402
9.	6.	0.	-0.824404	-0.078523	-1.149633
10.	6.	0.	0.166178	-0.949357	0.999325
11.	6.	0.	1.777105	-0.988419	1.001717
12.	6.	0.	2.175394	0.109032	0.001982
13.	6.	0.	-3.404829	-0.100996	1.095799
14.	6.	0.	-4.626565	-0.734699	0.467958
15.	6.	0.	-5.862129	-0.071228	0.472700
16.	6.	0.	-6.982199	-0.633116	-0.140134
17.	6.	0.	-6.885771	-1.873771	-0.772997
18.	6.	0.	-5.663379	-2.546766	-0.785254
19.	6.	0.	-4.545566	-1.981362	-0.169251
20.	6.	0.	0.900363	4.413168	0.885098
21.	6.	0.	-1.496429	3.193438	-1.269398
22.	8.	0.	-0.225802	-0.602469	-2.079417
23.	8.	0.	-0.367083	-0.878556	2.316550
24.	1.	0.	1.159246	1.334639	1.320074
25.	1.	0.	-1.343118	1.422813	1.783965
26.	6.	0.	2.386090	-2.351667	0.836087
27.	6.	0.	3.460377	-2.729274	0.121072
28.	6.	0.	4.315050	-1.855133	-0.801216
29.	6.	0.	5.149371	-0.683438	-0.170311
30.	6.	0.	4.619720	0.722180	-0.551345
31.	6.	0.	3.346783	1.080576	0.198368
32.	8.	0.	2.835092	2.401871	-0.230879
33.	6.	0.	6.627613	-0.809999	-0.574285
34.	6.	0.	3.910992	-4.172792	0.178115
35.	1.	0.	2.033226	-0.622475	2.009871
36.	1.	0.	2.120964	-0.258874	-1.025942
37.	1.	0.	-3.331947	1.636088	-0.164302
38.	1.	0.	-1.552971	3.542412	0.838913
39.	1.	0.	1.211008	2.293434	-1.527123
40.	1.	0.	-2.786036	-0.177415	-1.817305

41.	1.	0.	-0.220175	-1.836670	0.480215
42.	1.	0.	-2.716760	-0.863900	1.471390
43.	1.	0.	-3.704239	0.505493	1.959382
44.	1.	0.	-5.947671	0.892358	0.969985
45.	1.	0.	-7.931189	-0.104787	-0.119282
46.	1.	0.	-7.757430	-2.314406	-1.247857
47.	1.	0.	-5.579681	-3.515390	-1.269893
48.	1.	0.	-3.598646	-2.514715	-0.175680
49.	1.	0.	0.212783	5.050234	1.435045
50.	1.	0.	1.949909	4.689242	0.884163
51.	1.	0.	-1.280466	4.242199	-1.492270
52.	1.	0.	-1.005349	2.585910	-2.033100
53.	1.	0.	-2.574437	3.051455	-1.377632
54.	1.	0.	-0.112629	-1.683986	2.786884
55.	1.	0.	1.910913	-3.130129	1.437809
56.	1.	0.	5.022119	-2.527970	-1.298307
57.	1.	0.	3.694401	-1.460354	-1.611649
58.	1.	0.	5.097992	-0.779250	0.923230
59.	1.	0.	5.384455	1.476703	-0.332946
60.	1.	0.	4.431421	0.769718	-1.631698
61.	1.	0.	3.574069	1.155602	1.273486
62.	1.	0.	7.236808	-0.029029	-0.107076
63.	1.	0.	6.745652	-0.717830	-1.660687
64.	1.	0.	7.038347	-1.780702	-0.276727
65.	1.	0.	3.283154	-4.778168	0.837777
66.	1.	0.	3.893532	-4.628938	-0.820507
67.	1.	0.	4.947730	-4.246203	0.532751

Conformer **1-3**

**1-3**			Standard Orientation (Ångstroms)		
Center number	Atom number	Type	X	Y	Z
1.	6.	0.	1.640620	2.202217	0.072521
2.	6.	0.	1.689472	0.595412	0.209306
3.	6.	0.	0.302715	0.015342	0.607423
4.	6.	0.	-0.717930	0.810817	-0.130633
5.	6.	0.	-0.891526	2.285596	-0.030617
6.	6.	0.	0.397530	2.793998	-0.641953
7.	6.	0.	2.620124	0.123454	1.354375
8.	7.	0.	1.678884	0.064998	2.475568
9.	6.	0.	0.351984	0.065406	2.139780
10.	6.	0.	-0.224846	-1.445091	0.208619

11.	6.	0.	-1.737224	-1.264104	-0.285180
12.	6.	0.	-2.059040	0.210793	0.021369
13.	6.	0.	3.315353	-1.239367	1.104117
14.	6.	0.	4.414140	-1.163277	0.065570
15.	6.	0.	4.172419	-1.527595	-1.266580
16.	6.	0.	5.180504	-1.431120	-2.227357
17.	6.	0.	6.448052	-0.968374	-1.871974
18.	6.	0.	6.702252	-0.604447	-0.548293
19.	6.	0.	5.693102	-0.702616	0.409599
20.	6.	0.	0.468438	3.597177	-1.702471
21.	6.	0.	1.817906	2.984478	1.400002
22.	8.	0.	-0.587894	0.037108	2.922409
23.	8.	0.	0.607863	-1.967593	-0.824246
24.	1.	0.	-0.443632	0.649991	-1.188383
25.	1.	0.	2.018894	0.198464	-0.752239
26.	6.	0.	-2.785763	-2.183555	0.291283
27.	6.	0.	-4.081559	-2.134910	-0.065511
28.	6.	0.	-4.576838	-1.280788	-1.228844
29.	6.	0.	-5.221448	0.133817	-1.024621
30.	6.	0.	-4.322222	1.282649	-0.480656
31.	6.	0.	-2.853765	1.173873	-0.869182
32.	8.	0.	-2.157430	2.474493	-0.713573
33.	6.	0.	-6.525475	0.079644	-0.216781
34.	6.	0.	-5.097667	-3.029448	0.604535
35.	1.	0.	-1.682078	-1.383979	-1.377563
36.	1.	0.	-2.403919	0.294363	1.057383
37.	1.	0.	3.402650	0.860079	1.563613
38.	1.	0.	2.506372	2.453273	-0.551762
39.	1.	0.	-1.000791	2.632432	1.006817
40.	1.	0.	1.959062	-0.001094	3.444046
41.	1.	0.	-0.192362	-2.105751	1.085951
42.	1.	0.	3.736185	-1.576372	2.060104
43.	1.	0.	2.563584	-1.973399	0.801734
44.	1.	0.	3.185827	-1.888962	-1.542829
45.	1.	0.	4.975471	-1.721011	-3.254133
46.	1.	0.	7.233451	-0.895963	-2.618665
47.	1.	0.	7.687930	-0.249666	-0.260511
48.	1.	0.	5.903000	-0.427207	1.440979
49.	1.	0.	1.418609	3.863371	-2.157684
50.	1.	0.	-0.433765	4.004914	-2.147166
51.	1.	0.	1.827549	4.054713	1.175158
52.	1.	0.	1.009339	2.803518	2.112009
53.	1.	0.	2.759704	2.748257	1.901804
54.	1.	0.	0.259329	-2.833647	-1.075587

55.	1.	0.	-2.486186	-2.852653	1.097747
56.	1.	0.	-5.343323	-1.868478	-1.753276
57.	1.	0.	-3.761271	-1.165204	-1.950492
58.	1.	0.	-5.492505	0.423551	-2.050234
59.	1.	0.	-4.727745	2.231440	-0.849943
60.	1.	0.	-4.385034	1.326310	0.613750
61.	1.	0.	-2.758884	0.891315	-1.928456
62.	1.	0.	-7.026395	1.053882	-0.224500
63.	1.	0.	-6.334250	-0.179161	0.830105
64.	1.	0.	-7.223585	-0.658266	-0.626881
65.	1.	0.	-4.639871	-3.642260	1.386132
66.	1.	0.	-5.908170	-2.451303	1.062953
67.	1.	0.	-5.571537	-3.702425	-0.122747

Conformer **1-4**

**1 -4**			Standard Orientation (Ångstroms)		
Center number	Atom number	Type	X	Y	Z
1.	6.	0.	-0.999046	2.881860	0.204991
2.	6.	0.	-1.319527	1.383179	0.704652
3.	6.	0.	-0.259447	0.373133	0.187286
4.	6.	0.	1.060182	1.057888	0.244133
5.	6.	0.	1.398593	2.339806	-0.432623
6.	6.	0.	0.485134	3.320245	0.276026
7.	6.	0.	-2.654526	0.836223	0.135082
8.	7.	0.	-2.190810	0.170923	-1.082747
9.	6.	0.	-0.842844	-0.051378	-1.162538
10.	6.	0.	0.171182	-0.988194	0.955779
11.	6.	0.	1.777447	-0.991282	0.985848
12.	6.	0.	2.170528	0.106920	-0.012536
13.	6.	0.	-3.410776	-0.107177	1.102955
14.	6.	0.	-4.630073	-0.738758	0.467969
15.	6.	0.	-5.860998	-0.066910	0.451325
16.	6.	0.	-6.977427	-0.627943	-0.168829
17.	6.	0.	-6.881445	-1.875933	-0.787286
18.	6.	0.	-5.663576	-2.557073	-0.777720
19.	6.	0.	-4.549147	-1.992504	-0.154817
20.	6.	0.	0.906989	4.394510	0.941746
21.	6.	0.	-1.508590	3.220677	-1.220437
22.	8.	0.	-0.250258	-0.560898	-2.104255
23.	8.	0.	-0.394207	-1.160537	2.241182
24.	1.	0.	1.159807	1.321273	1.314903

25.	1.	0.	-1.348678	1.402912	1.798960
26.	6.	0.	2.408608	-2.347046	0.855515
27.	6.	0.	3.488674	-2.719507	0.146859
28.	6.	0.	4.323003	-1.851509	-0.799919
29.	6.	0.	5.161723	-0.669194	-0.195057
30.	6.	0.	4.612166	0.730431	-0.570155
31.	6.	0.	3.342124	1.077474	0.188990
32.	8.	0.	2.828447	2.404492	-0.221041
33.	6.	0.	6.630803	-0.783722	-0.634137
34.	6.	0.	3.968699	-4.151675	0.240430
35.	1.	0.	2.027595	-0.612987	1.994051
36.	1.	0.	2.114507	-0.258269	-1.041340
37.	1.	0.	-3.339708	1.647754	-0.132543
38.	1.	0.	-1.549624	3.534060	0.893921
39.	1.	0.	1.199366	2.321148	-1.513361
40.	1.	0.	-2.811967	-0.152019	-1.810834
41.	1.	0.	-0.181106	-1.845512	0.377420
42.	1.	0.	-2.722363	-0.872243	1.473774
43.	1.	0.	-3.715108	0.493459	1.969506
44.	1.	0.	-5.946361	0.902237	0.937893
45.	1.	0.	-7.923196	-0.093478	-0.164727
46.	1.	0.	-7.750369	-2.316050	-1.267614
47.	1.	0.	-5.580734	-3.531652	-1.250321
48.	1.	0.	-3.606134	-2.532406	-0.142550
49.	1.	0.	0.224947	5.023578	1.507646
50.	1.	0.	1.957194	4.668015	0.938032
51.	1.	0.	-1.287739	4.271471	-1.428097
52.	1.	0.	-1.027495	2.622094	-1.997368
53.	1.	0.	-2.588221	3.087037	-1.321726
54.	1.	0.	-0.026950	-0.483702	2.826886
55.	1.	0.	1.940616	-3.107993	1.481273
56.	1.	0.	5.025601	-2.526390	-1.300503
57.	1.	0.	3.688829	-1.468108	-1.605401
58.	1.	0.	5.137499	-0.760072	0.899910
59.	1.	0.	5.370285	1.493133	-0.357176
60.	1.	0.	4.414825	0.776502	-1.648994
61.	1.	0.	3.574838	1.140285	1.263981
62.	1.	0.	7.243489	0.006917	-0.188167
63.	1.	0.	6.721160	-0.699354	-1.723824
64.	1.	0.	7.058222	-1.747883	-0.339190
65.	1.	0.	3.356513	-4.749861	0.920665
66.	1.	0.	3.955032	-4.635743	-0.745079
67.	1.	0.	5.008649	-4.194336	0.590946

Conformer **1-5**

**1-5**			Standard Orientation (Ångstroms)		
Center number	Atom number	Type	X	Y	Z
1.	6.	0.	-1.031365	2.851759	0.200489
2.	6.	0.	-1.343507	1.349243	0.701083
3.	6.	0.	-0.287240	0.341062	0.168163
4.	6.	0.	1.028222	1.037155	0.215437
5.	6.	0.	1.348871	2.309858	-0.486034
6.	6.	0.	0.454419	3.295870	0.236575
7.	6.	0.	-2.681490	0.801637	0.142222
8.	7.	0.	-2.230236	0.147845	-1.087284
9.	6.	0.	-0.884757	-0.084355	-1.178619
10.	6.	0.	0.147746	-1.002276	0.928073
11.	6.	0.	1.746272	-1.068792	0.857583
12.	6.	0.	2.128342	0.095846	-0.076631
13.	6.	0.	-3.417848	-0.157109	1.110808
14.	6.	0.	-4.649958	-0.780780	0.493166
15.	6.	0.	-5.886773	-0.120989	0.536574
16.	6.	0.	-7.017055	-0.671956	-0.067278
17.	6.	0.	-6.929998	-1.897720	-0.729803
18.	6.	0.	-5.706537	-2.566919	-0.780647
19.	6.	0.	-4.578396	-2.012477	-0.173504
20.	6.	0.	0.886676	4.377080	0.883788
21.	6.	0.	-1.576676	3.204120	-1.208573
22.	8.	0.	-0.305404	-0.600544	-2.124635
23.	8.	0.	-0.324797	-0.934055	2.269004
24.	1.	0.	1.138586	1.291743	1.284652
25.	1.	0.	-1.350075	1.358264	1.793513
26.	6.	0.	2.380992	-2.363017	0.411465
27.	6.	0.	3.711675	-2.539133	0.328374
28.	6.	0.	4.706616	-1.512142	0.862293
29.	6.	0.	5.412192	-0.459074	-0.060913
30.	6.	0.	4.537944	0.652973	-0.713157
31.	6.	0.	3.314367	1.052120	0.099764
32.	8.	0.	2.789378	2.370931	-0.332079
33.	6.	0.	6.296343	-1.108278	-1.134473
34.	6.	0.	4.285748	-3.834348	-0.194862
35.	1.	0.	2.075214	-0.812715	1.876018
36.	1.	0.	2.078229	-0.247358	-1.115240
37.	1.	0.	-3.376661	1.609374	-0.109822
38.	1.	0.	-1.564290	3.498519	0.908003
39.	1.	0.	1.113114	2.275847	-1.559037

40.	1.	0.	-2.858358	-0.169939	-1.811761
41.	1.	0.	-0.284915	-1.874939	0.418806
42.	1.	0.	-2.720045	-0.926392	1.454764
43.	1.	0.	-3.701527	0.427596	1.994399
44.	1.	0.	-5.965099	0.831033	1.056773
45.	1.	0.	-7.966650	-0.146769	-0.016406
46.	1.	0.	-7.809578	-2.329877	-1.197776
47.	1.	0.	-5.629868	-3.524113	-1.288586
48.	1.	0.	-3.630603	-2.543085	-0.210077
49.	1.	0.	0.216024	5.006299	1.462896
50.	1.	0.	1.935303	4.655154	0.853221
51.	1.	0.	-1.359079	4.256277	-1.412792
52.	1.	0.	-1.117813	2.611737	-2.003593
53.	1.	0.	-2.659156	3.074305	-1.283259
54.	1.	0.	-0.024249	-1.728085	2.731536
55.	1.	0.	1.721398	-3.156519	0.060535
56.	1.	0.	4.234210	-0.961613	1.682740
57.	1.	0.	5.527144	-2.075280	1.328367
58.	1.	0.	6.084812	0.058798	0.638020
59.	1.	0.	5.172929	1.531918	-0.871783
60.	1.	0.	4.200115	0.333728	-1.706833
61.	1.	0.	3.578105	1.152194	1.163177
62.	1.	0.	6.885061	-0.349451	-1.661554
63.	1.	0.	5.691983	-1.629474	-1.884546
64.	1.	0.	6.995637	-1.830667	-0.699615
65.	1.	0.	3.498313	-4.530811	-0.496619
66.	1.	0.	4.937255	-3.669377	-1.060678
67.	1.	0.	4.903806	-4.328043	0.567060

Table S3. Gibbs free energies^{a} and equilibrium populations^{b} of low-energy conformers of phomoparagin B (**2**)

Conformers	In MeOH	
	G^{a}	P (%)^{b}
**2-1**	-1002150.34750896	84.59
**2-2**	-1002148.90486347	7.39
**2-3**	-1002148.50074703	3.74
**2-4**	-1002147.36369891	0.55
**2-5**	-1002148.50011952	3.73

^{a}B3LYP/6-31G(d,p), in kcal/mol. ^{b}From G values at 298.15K.

Table S4. Cartesian coordinates for the low-energy reoptimized MMFF conformers of phomoparagin B (**2**) at B3LYP/6-31G(d,p) level of theory in gas

Conformer **2-1**

**2-1**			Standard Orientation (Ångstroms)		
Center number	Atomic number	Atomic Type	X	Y	Z
1.	6.	0.	-1.314667	2.370031	1.391352
2.	6.	0.	-1.404632	0.857806	1.007383
3.	6.	0.	-0.157924	0.429497	0.176252
4.	6.	0.	0.995281	1.466400	0.209702
5.	6.	0.	0.548215	2.858611	-0.291488
6.	6.	0.	-0.881669	3.151298	0.168013
7.	6.	0.	-2.649573	0.527658	0.124597
8.	7.	0.	-2.070098	0.205018	-1.167370
9.	6.	0.	-0.718385	0.197298	-1.248415
10.	6.	0.	0.561423	-0.897094	0.565722
11.	6.	0.	2.060247	-0.700434	0.207202
12.	6.	0.	2.175725	0.711922	-0.454391
13.	6.	0.	-3.524426	-0.618011	0.685272
14.	6.	0.	-4.684006	-0.970551	-0.221026
15.	6.	0.	-5.878497	-0.236522	-0.180800
16.	6.	0.	-6.932515	-0.532344	-1.044885
17.	6.	0.	-6.809629	-1.571254	-1.969735
18.	6.	0.	-5.627983	-2.311453	-2.020123
19.	6.	0.	-4.575469	-2.013202	-1.152503
20.	6.	0.	-1.640905	4.032049	-0.485904
21.	6.	0.	-2.560960	2.890668	2.112938
22.	8.	0.	-0.072088	-0.024211	-2.267325
23.	8.	0.	0.488356	-1.138608	1.996286
24.	6.	0.	-0.270275	-2.173677	2.434180
25.	8.	0.	-0.960427	-2.868988	1.719559
26.	6.	0.	-0.124671	-2.339937	3.927805
27.	8.	0.	1.360164	3.892572	0.249747
28.	1.	0.	1.259982	1.607294	1.268211
29.	1.	0.	-1.459950	0.284618	1.936984
30.	6.	0.	2.637268	-1.848833	-0.576890
31.	6.	0.	3.899063	-2.295162	-0.479322
32.	6.	0.	4.902469	-1.611137	0.427674
33.	6.	0.	5.624844	-0.375933	-0.192318
34.	6.	0.	4.724072	0.576715	-1.008866
35.	6.	0.	3.575073	1.335468	-0.297884
36.	8.	0.	3.568945	2.692882	-0.808335
37.	6.	0.	6.821510	-0.796133	-1.063544
38.	6.	0.	4.376653	-3.476577	-1.286744
39.	1.	0.	2.570537	-0.631443	1.174992

40.	1.	0.	1.964716	0.609822	-1.524753
41.	1.	0.	-3.281841	1.415639	0.010838
42.	1.	0.	-0.492102	2.445907	2.115309
43.	1.	0.	0.573117	2.874979	-1.393910
44.	1.	0.	-2.630116	0.016926	-1.987132
45.	1.	0.	0.118685	-1.760907	0.069626
46.	1.	0.	-2.892483	-1.495932	0.854719
47.	1.	0.	-3.902097	-0.294765	1.663388
48.	1.	0.	-5.985219	0.570384	0.540712
49.	1.	0.	-7.851291	0.045050	-0.993436
50.	1.	0.	-7.630451	-1.804889	-2.641399
51.	1.	0.	-5.525480	-3.126411	-2.731052
52.	1.	0.	-3.661900	-2.600784	-1.188157
53.	1.	0.	-2.637756	4.304322	-0.153792
54.	1.	0.	-1.272280	4.535859	-1.374886
55.	1.	0.	-2.405496	3.923542	2.437576
56.	1.	0.	-3.458088	2.876840	1.486989
57.	1.	0.	-2.769764	2.285497	3.002012
58.	1.	0.	-0.321648	-1.393086	4.437842
59.	1.	0.	0.902696	-2.631045	4.167041
60.	1.	0.	-0.814505	-3.107474	4.276566
61.	1.	0.	2.270366	3.681317	-0.033988
62.	1.	0.	1.959966	-2.329158	-1.281948
63.	1.	0.	4.399559	-1.285960	1.344695
64.	1.	0.	5.669272	-2.327846	0.746698
65.	1.	0.	6.028431	0.199152	0.653592
66.	1.	0.	5.383571	1.360874	-1.397203
67.	1.	0.	4.323541	0.041441	-1.878517
68.	1.	0.	3.809892	1.462681	0.766802
69.	1.	0.	3.370651	2.643930	-1.755097
70.	1.	0.	7.398433	0.077061	-1.386355
71.	1.	0.	6.495299	-1.323895	-1.966296
72.	1.	0.	7.497360	-1.459447	-0.513998
73.	1.	0.	4.743118	-4.275125	-0.628111
74.	1.	0.	3.576750	-3.889708	-1.907413
75.	1.	0.	5.213675	-3.212541	-1.944548

Conformer **2-2**

**2-2**			Standard Orientation (Ångstroms)		
Center number	Atom number	Type	X	Y	Z
1.	6.	0.	-1.331739	2.426426	1.302290

2.	6.	0.	-1.412987	0.899897	0.978459
3.	6.	0.	-0.157963	0.442239	0.176062
4.	6.	0.	0.992162	1.484997	0.177004
5.	6.	0.	0.540481	2.852485	-0.386135
6.	6.	0.	-0.893289	3.157849	0.050500
7.	6.	0.	-2.649581	0.531912	0.098537
8.	7.	0.	-2.058213	0.160536	-1.174395
9.	6.	0.	-0.705272	0.139892	-1.240476
10.	6.	0.	0.561872	-0.861551	0.625677
11.	6.	0.	2.052719	-0.692154	0.223171
12.	6.	0.	2.178714	0.712518	-0.456010
13.	6.	0.	-3.524943	-0.594587	0.696814
14.	6.	0.	-4.671852	-0.992316	-0.206719
15.	6.	0.	-4.551172	-2.080771	-1.082593
16.	6.	0.	-5.591765	-2.423024	-1.948362
17.	6.	0.	-6.773643	-1.681511	-1.951806
18.	6.	0.	-6.908660	-0.597049	-1.082600
19.	6.	0.	-5.866560	-0.257546	-0.220183
20.	6.	0.	-1.652115	4.006885	-0.644640
21.	6.	0.	-2.585379	2.970048	1.993284
22.	8.	0.	-0.050198	-0.144194	-2.237310
23.	8.	0.	0.519339	-1.012595	2.071033
24.	6.	0.	-0.244119	-2.005701	2.588789
25.	8.	0.	-0.958764	-2.734771	1.934301
26.	6.	0.	-0.069637	-2.079730	4.086964
27.	8.	0.	1.344945	3.910498	0.121596
28.	1.	0.	1.247015	1.667599	1.231618
29.	1.	0.	-1.475733	0.364148	1.929739
30.	6.	0.	2.587301	-1.851845	-0.574154
31.	6.	0.	3.838716	-2.329703	-0.496391
32.	6.	0.	4.870141	-1.677297	0.403228
33.	6.	0.	5.606384	-0.447003	-0.211428
34.	6.	0.	4.711852	0.525236	-1.010707
35.	6.	0.	3.588070	1.303324	-0.276144
36.	8.	0.	3.542856	2.639521	-0.827666
37.	6.	0.	6.786682	-0.880830	-1.098586
38.	6.	0.	4.278120	-3.515456	-1.319142
39.	1.	0.	2.590632	-0.631062	1.175950
40.	1.	0.	1.992550	0.607433	-1.528073
41.	1.	0.	-3.283720	1.412755	-0.053972
42.	1.	0.	-0.514507	2.535023	2.028210
43.	1.	0.	0.581594	2.825039	-1.486109
44.	1.	0.	-2.610531	-0.072240	-1.987871
45.	1.	0.	0.105277	-1.752761	0.194924

46.	1.	0.	-2.890323	-1.460695	0.911030
47.	1.	0.	-3.915532	-0.232999	1.656298
48.	1.	0.	-3.637268	-2.668953	-1.076070
49.	1.	0.	-5.479683	-3.272815	-2.615726
50.	1.	0.	-7.585227	-1.948948	-2.622102
51.	1.	0.	-7.827738	-0.017891	-1.073347
52.	1.	0.	-5.982674	0.584999	0.457804
53.	1.	0.	-2.652509	4.288043	-0.330724
54.	1.	0.	-1.279811	4.473926	-1.551938
55.	1.	0.	-2.436396	4.015715	2.277607
56.	1.	0.	-3.477424	2.928026	1.361249
57.	1.	0.	-2.799372	2.399711	2.903983
58.	1.	0.	-0.242941	-1.099709	4.539705
59.	1.	0.	0.958286	-2.370854	4.323828
60.	1.	0.	-0.762684	-2.813186	4.497309
61.	1.	0.	2.251469	3.683671	-0.158444
62.	1.	0.	1.888575	-2.305452	-1.275518
63.	1.	0.	4.389048	-1.356168	1.333134
64.	1.	0.	5.628327	-2.412836	0.698914
65.	1.	0.	6.028919	0.111121	0.637490
66.	1.	0.	5.387529	1.282218	-1.431154
67.	1.	0.	4.282699	0.007632	-1.875118
68.	1.	0.	3.819782	1.373078	0.800525
69.	1.	0.	4.427978	3.021435	-0.760603
70.	1.	0.	7.376017	-0.016373	-1.423458
71.	1.	0.	6.440979	-1.397111	-2.000516
72.	1.	0.	7.456953	-1.559048	-0.560680
73.	1.	0.	4.637840	-4.326586	-0.672178
74.	1.	0.	3.459688	-3.906431	-1.929800
75.	1.	0.	5.109779	-3.264673	-1.988996

Conformer **2-3**

**2-3**			Standard Orientation (Ångstroms)		
Center number	Atom number	Type	X	Y	Z
1.	6.	0.	1.798375	1.830153	-0.904979
2.	6.	0.	1.615329	0.547838	-0.028125
3.	6.	0.	0.118811	0.384078	0.387362
4.	6.	0.	-0.852390	1.311082	-0.396756
5.	6.	0.	-0.477308	2.805177	-0.272145
6.	6.	0.	1.042610	2.971335	-0.256789
7.	6.	0.	2.430736	0.589933	1.303589

8.	7.	0.	1.402540	0.786542	2.312243
9.	6.	0.	0.115171	0.675333	1.907684
10.	6.	0.	-0.544729	-1.010060	0.205572
11.	6.	0.	-2.066101	-0.745703	0.026071
12.	6.	0.	-2.253118	0.806911	0.034758
13.	6.	0.	3.261559	-0.682533	1.607513
14.	6.	0.	4.478447	-0.856532	0.723538
15.	6.	0.	4.533214	-1.876660	-0.235804
16.	6.	0.	5.654421	-2.028008	-1.055219
17.	6.	0.	6.740365	-1.161962	-0.927718
18.	6.	0.	6.703020	-0.148305	0.032413
19.	6.	0.	5.583502	-0.001192	0.850281
20.	6.	0.	1.604496	4.051781	0.287855
21.	6.	0.	3.254363	2.119858	-1.279284
22.	8.	0.	-0.862177	0.749578	2.645974
23.	8.	0.	-0.083704	-1.637425	-1.024352
24.	6.	0.	0.755603	-2.693230	-0.939448
25.	8.	0.	1.209044	-3.129292	0.099582
26.	6.	0.	1.080284	-3.231380	-2.312342
27.	8.	0.	-0.949074	3.553524	-1.385343
28.	1.	0.	-0.728343	1.062256	-1.460749
29.	1.	0.	1.950791	-0.303460	-0.624845
30.	6.	0.	-2.934128	-1.497873	0.997806
31.	6.	0.	-4.143092	-2.003043	0.708246
32.	6.	0.	-4.754923	-1.825308	-0.668135
33.	6.	0.	-5.502776	-0.477052	-0.906145
34.	6.	0.	-4.839415	0.780176	-0.298424
35.	6.	0.	-3.457069	1.258612	-0.811958
36.	8.	0.	-3.498376	2.706274	-0.880794
37.	6.	0.	-6.959532	-0.548711	-0.414070
38.	6.	0.	-4.950702	-2.747785	1.741928
39.	1.	0.	-2.287564	-1.084465	-0.991275
40.	1.	0.	-2.429722	1.123404	1.068495
41.	1.	0.	3.102099	1.455410	1.308553
42.	1.	0.	1.281646	1.615437	-1.850233
43.	1.	0.	-0.884996	3.206150	0.671516
44.	1.	0.	1.606224	0.916671	3.293529
45.	1.	0.	-0.321375	-1.692943	1.025627
46.	1.	0.	3.585516	-0.618728	2.655393
47.	1.	0.	2.608999	-1.557295	1.528756
48.	1.	0.	3.698923	-2.567498	-0.319704
49.	1.	0.	5.681413	-2.829320	-1.788541
50.	1.	0.	7.612909	-1.280066	-1.563568
51.	1.	0.	7.548836	0.523556	0.148048

52.	1.	0.	5.570300	0.783100	1.603871
53.	1.	0.	2.675209	4.228136	0.263257
54.	1.	0.	0.997530	4.819358	0.759772
55.	1.	0.	3.705285	1.251605	-1.769688
56.	1.	0.	3.300273	2.966060	-1.971167
57.	1.	0.	3.882388	2.364301	-0.418213
58.	1.	0.	1.456145	-4.250419	-2.220922
59.	1.	0.	1.859582	-2.608160	-2.764294
60.	1.	0.	0.206237	-3.199287	-2.965449
61.	1.	0.	-1.914096	3.405871	-1.408894
62.	1.	0.	-2.542610	-1.593905	2.009229
63.	1.	0.	-3.970209	-1.919108	-1.426313
64.	1.	0.	-5.458744	-2.641998	-0.870359
65.	1.	0.	-5.538913	-0.333927	-1.995781
66.	1.	0.	-5.519349	1.612243	-0.513407
67.	1.	0.	-4.802448	0.677616	0.793426
68.	1.	0.	-3.316583	0.955050	-1.857285
69.	1.	0.	-3.649373	3.033225	0.018362
70.	1.	0.	-7.514616	0.353639	-0.691880
71.	1.	0.	-7.011847	-0.641576	0.676381
72.	1.	0.	-7.480815	-1.408570	-0.847307
73.	1.	0.	-5.174338	-3.767687	1.401945
74.	1.	0.	-4.419110	-2.816527	2.695036
75.	1.	0.	-5.918889	-2.266793	1.928792

Conformer **2-4**

**2 -4**			Standard Orientation (Ångstroms)		
Center number	Atom number	Type	X	Y	Z
1.	6.	0.	1.810072	1.833219	-0.883926
2.	6.	0.	1.616869	0.542264	-0.022864
3.	6.	0.	0.117137	0.379124	0.381742
4.	6.	0.	-0.845915	1.319558	-0.398417
5.	6.	0.	-0.463326	2.809552	-0.243418
6.	6.	0.	1.057370	2.968892	-0.223270
7.	6.	0.	2.424406	0.568596	1.314052
8.	7.	0.	1.391078	0.755437	2.318762
9.	6.	0.	0.105283	0.647677	1.906293
10.	6.	0.	-0.549256	-1.008831	0.182303
11.	6.	0.	-2.071000	-0.737128	0.023699
12.	6.	0.	-2.252816	0.817580	0.018895
13.	6.	0.	3.251289	-0.709249	1.607005

14.	6.	0.	4.475129	-0.871575	0.730470
15.	6.	0.	4.533708	-1.872231	-0.248980
16.	6.	0.	5.661280	-2.011661	-1.061768
17.	6.	0.	6.749729	-1.153078	-0.907607
18.	6.	0.	6.708184	-0.158605	0.072210
19.	6.	0.	5.582352	-0.023332	0.883403
20.	6.	0.	1.622760	4.040218	0.335755
21.	6.	0.	3.268927	2.119526	-1.249409
22.	8.	0.	-0.875305	0.704421	2.640739
23.	8.	0.	-0.101683	-1.612898	-1.065215
24.	6.	0.	0.742742	-2.666006	-1.009192
25.	8.	0.	1.208477	-3.120946	0.016333
26.	6.	0.	1.056020	-3.176229	-2.395557
27.	8.	0.	-0.929428	3.579289	-1.345306
28.	1.	0.	-0.713034	1.084296	-1.464488
29.	1.	0.	1.953843	-0.304151	-0.625783
30.	6.	0.	-2.927668	-1.474051	1.017519
31.	6.	0.	-4.138588	-1.986073	0.748987
32.	6.	0.	-4.765316	-1.831513	-0.623577
33.	6.	0.	-5.510767	-0.484848	-0.877286
34.	6.	0.	-4.829793	0.777961	-0.302820
35.	6.	0.	-3.451122	1.233774	-0.851410
36.	8.	0.	-3.449245	2.679011	-0.886311
37.	6.	0.	-6.959881	-0.540228	-0.360960
38.	6.	0.	-4.934248	-2.714890	1.803189
39.	1.	0.	-2.309627	-1.088470	-0.985249
40.	1.	0.	-2.450272	1.153566	1.039952
41.	1.	0.	3.097604	1.432522	1.332051
42.	1.	0.	1.295691	1.632697	-1.833610
43.	1.	0.	-0.876753	3.194086	0.702450
44.	1.	0.	1.589239	0.870241	3.303063
45.	1.	0.	-0.318890	-1.708559	0.985984
46.	1.	0.	3.566734	-0.661064	2.658277
47.	1.	0.	2.598636	-1.582065	1.509638
48.	1.	0.	3.696755	-2.556895	-0.354776
49.	1.	0.	5.691121	-2.797950	-1.811101
50.	1.	0.	7.627201	-1.262091	-1.538313
51.	1.	0.	7.555565	0.507450	0.208321
52.	1.	0.	5.565524	0.746092	1.652108
53.	1.	0.	2.694306	4.212566	0.315195
54.	1.	0.	1.017572	4.803615	0.816554
55.	1.	0.	3.716519	1.255877	-1.751039
56.	1.	0.	3.322041	2.975564	-1.928640
57.	1.	0.	3.895663	2.347783	-0.382973

58.	1.	0.	1.440763	-4.193757	-2.327336
59.	1.	0.	1.824920	-2.538519	-2.845050
60.	1.	0.	0.174177	-3.139157	-3.037900
61.	1.	0.	-1.894909	3.440651	-1.352521
62.	1.	0.	-2.526023	-1.549889	2.026378
63.	1.	0.	-3.989852	-1.941734	-1.388897
64.	1.	0.	-5.474638	-2.648738	-0.803245
65.	1.	0.	-5.563193	-0.365768	-1.969846
66.	1.	0.	-5.523852	1.606382	-0.499003
67.	1.	0.	-4.770399	0.702349	0.788295
68.	1.	0.	-3.308343	0.853890	-1.877566
69.	1.	0.	-4.209552	2.967957	-1.408045
70.	1.	0.	-7.517624	0.357955	-0.647945
71.	1.	0.	-6.994203	-0.609539	0.731735
72.	1.	0.	-7.491187	-1.406853	-0.767577
73.	1.	0.	-5.158689	-3.741176	1.483306
74.	1.	0.	-4.392599	-2.765622	2.751693
75.	1.	0.	-5.901681	-2.233120	1.992280

Conformer **2-5**

**2-5**			Standard Orientation (Ångstroms)		
Center number	Atom number	Type	X	Y	Z
1.	6.	0.	1.798469	1.829715	-0.905035
2.	6.	0.	1.615324	0.547492	-0.028101
3.	6.	0.	0.118779	0.383827	0.387383
4.	6.	0.	-0.852340	1.310959	-0.396738
5.	6.	0.	-0.477119	2.805003	-0.272107
6.	6.	0.	1.042820	2.971009	-0.256880
7.	6.	0.	2.430709	0.589656	1.303638
8.	7.	0.	1.402492	0.786135	2.312285
9.	6.	0.	0.115129	0.675108	1.907680
10.	6.	0.	-0.544889	-1.010226	0.205498
11.	6.	0.	-2.066239	-0.745733	0.025966
12.	6.	0.	-2.253103	0.806905	0.034733
13.	6.	0.	3.261687	-0.682727	1.607589
14.	6.	0.	4.478621	-0.856497	0.723659
15.	6.	0.	5.583686	-0.001224	0.850795
16.	6.	0.	6.703235	-0.148010	0.032914
17.	6.	0.	6.740598	-1.161270	-0.927635
18.	6.	0.	5.654649	-2.027257	-1.055525
19.	6.	0.	4.533414	-1.876239	-0.236090

20.	6.	0.	1.604865	4.051405	0.287701
21.	6.	0.	3.254479	2.119262	-1.279390
22.	8.	0.	-0.862237	0.749550	2.645934
23.	8.	0.	-0.083921	-1.637595	-1.024446
24.	6.	0.	0.755517	-2.693322	-0.939556
25.	8.	0.	1.209150	-3.129218	0.099449
26.	6.	0.	1.079905	-3.231626	-2.312460
27.	8.	0.	-0.948975	3.553428	-1.385196
28.	1.	0.	-0.728275	1.062136	-1.460731
29.	1.	0.	1.950756	-0.303894	-0.624708
30.	6.	0.	-2.934338	-1.497968	0.997585
31.	6.	0.	-4.143376	-2.002956	0.707995
32.	6.	0.	-4.755320	-1.824835	-0.668287
33.	6.	0.	-5.503117	-0.476480	-0.905866
34.	6.	0.	-4.839379	0.780589	-0.298214
35.	6.	0.	-3.457022	1.258720	-0.811977
36.	8.	0.	-3.498084	2.706385	-0.881040
37.	6.	0.	-6.959705	-0.548007	-0.413295
38.	6.	0.	-4.950946	-2.747897	1.741566
39.	1.	0.	-2.287671	-1.084393	-0.991422
40.	1.	0.	-2.429691	1.123367	1.068481
41.	1.	0.	3.102001	1.455192	1.308629
42.	1.	0.	1.281710	1.614997	-1.850272
43.	1.	0.	-0.884645	3.205962	0.671638
44.	1.	0.	1.606165	0.916702	3.293513
45.	1.	0.	-0.321604	-1.693166	1.025526
46.	1.	0.	3.585576	-0.618841	2.655481
47.	1.	0.	2.609232	-1.557565	1.528800
48.	1.	0.	5.570439	0.782753	1.604715
49.	1.	0.	7.549060	0.523783	0.148868
50.	1.	0.	7.613155	-1.279126	-1.563511
51.	1.	0.	5.681666	-2.828265	-1.789179
52.	1.	0.	3.699143	-2.567069	-0.320253
53.	1.	0.	2.675599	4.227620	0.263029
54.	1.	0.	0.998024	4.819065	0.759643
55.	1.	0.	3.300457	2.965464	-1.971267
56.	1.	0.	3.882580	2.363598	-0.418346
57.	1.	0.	3.705272	1.250956	-1.769823
58.	1.	0.	1.858419	-2.607880	-2.765043
59.	1.	0.	0.205463	-3.200370	-2.965083
60.	1.	0.	1.456609	-4.250339	-2.220962
61.	1.	0.	-1.914006	3.405658	-1.408718
62.	1.	0.	-2.542777	-1.594288	2.008965
63.	1.	0.	-3.970682	-1.918449	-1.426566

64.	1.	0.	-5.459217	-2.641421	-0.870670
65.	1.	0.	-5.539599	-0.333227	-1.995475
66.	1.	0.	-5.519200	1.612794	-0.513022
67.	1.	0.	-4.802221	0.677945	0.793621
68.	1.	0.	-3.316659	0.954984	-1.857270
69.	1.	0.	-3.649237	3.033505	0.018029
70.	1.	0.	-7.514777	0.354428	-0.690851
71.	1.	0.	-7.011647	-0.640936	0.677167
72.	1.	0.	-7.481242	-1.407774	-0.846408
73.	1.	0.	-5.174660	-3.767705	1.401349
74.	1.	0.	-4.419275	-2.816903	2.694610
75.	1.	0.	-5.919098	-2.266917	1.928630

Table S5. Gibbs free energies[a] and equilibrium populations[b] of low-energy conformers of phomoparagin C (**3**)

Conformers	In MeOH	
	G[a]	*P* (%)[b]
**3.-1**	-858418.17485541	83.29
**3.-2**	-858417.04910247	12.43
**3.-3**	-858416.25341979	3.24
**3.-4**	-858415.08938874	0.45
**3.-5**	-858415.23371604	0.58

[a]B3LYP/6-31G(d,p), in kcal/mol. [b]From *G* values at 298.15K.

Table S6. Cartesian coordinates for the low-energy reoptimized MMFF conformers of phomoparagin C (**3**) at B3LYP/6-31G(d,p) level of theory in gas

Conformer **3-1**

**3-1**			Standard Orientation (Ångstroms)		
Center number	Atomic number	Atomic Type	X	Y	Z
1.	6.	0.	1.331162	2.535225	-1.188791
2.	6.	0.	1.367422	0.959495	-1.165531
3.	6.	0.	0.127122	0.400797	-0.387851
4.	6.	0.	-1.039566	1.422022	-0.422052
5.	6.	0.	-0.727413	2.710286	0.330457
6.	6.	0.	0.748735	3.060031	0.113917
7.	6.	0.	2.610211	0.364852	-0.442840
8.	7.	0.	2.078593	-0.020621	0.853728
9.	6.	0.	0.732748	0.011458	0.986786
10.	6.	0.	-0.587509	-0.827417	-0.902422

11.	6.	0.	-1.933915	-0.745198	-0.796470
12.	6.	0.	-2.370857	0.626716	-0.263717
13.	6.	0.	3.254176	-0.824413	-1.197680
14.	6.	0.	4.418628	-1.437551	-0.450952
15.	6.	0.	5.704887	-0.889039	-0.559290
16.	6.	0.	6.774226	-1.420426	0.161181
17.	6.	0.	6.575061	-2.513534	1.006745
18.	6.	0.	5.301353	-3.070928	1.123122
19.	6.	0.	4.233343	-2.536597	0.399764
20.	6.	0.	1.419140	3.770756	1.025343
21.	6.	0.	2.647375	3.181745	-1.632164
22.	8.	0.	0.136234	-0.319396	2.010099
23.	8.	0.	-1.573997	3.714882	-0.258180
24.	1.	0.	-1.065612	1.726633	-1.474965
25.	1.	0.	1.355238	0.615381	-2.205416
26.	6.	0.	-2.795312	-1.836941	-1.242933
27.	6.	0.	-4.141355	-1.919054	-1.325794
28.	6.	0.	-5.100126	-0.823602	-0.900620
29.	6.	0.	-5.309742	-0.673905	0.632930
30.	6.	0.	-4.012906	-0.506183	1.443847
31.	6.	0.	-3.098069	0.693221	1.127770
32.	8.	0.	-2.230846	0.904698	2.226266
33.	6.	0.	-6.128319	-1.841783	1.206348
34.	6.	0.	-4.779698	-3.168519	-1.882101
35.	1.	0.	-3.081902	1.061158	-0.972159
36.	1.	0.	3.386932	1.122005	-0.297665
37.	1.	0.	0.598371	2.792405	-1.964760
38.	1.	0.	-0.942809	2.606596	1.397596
39.	1.	0.	2.658758	-0.352826	1.611511
40.	1.	0.	-0.060390	-1.709392	-1.256259
41.	1.	0.	2.485911	-1.582471	-1.387787
42.	1.	0.	3.590197	-0.454520	-2.173905
43.	1.	0.	5.870270	-0.041851	-1.220993
44.	1.	0.	7.763838	-0.984370	0.058864
45.	1.	0.	7.407349	-2.930333	1.566042
46.	1.	0.	5.137537	-3.925257	1.773551
47.	1.	0.	3.245629	-2.981490	0.489602
48.	1.	0.	2.449863	4.082375	0.887987
49.	1.	0.	0.946551	4.070286	1.957692
50.	1.	0.	2.500125	4.251730	-1.806314
51.	1.	0.	3.448183	3.081849	-0.893704
52.	1.	0.	3.001862	2.736012	-2.568135
53.	1.	0.	-1.422172	4.534042	0.231578
54.	1.	0.	-2.235886	-2.708311	-1.584815

55.	1.	0.	-4.773705	0.136747	-1.307467
56.	1.	0.	-6.079551	-1.016713	-1.355213
57.	1.	0.	-5.905558	0.241765	0.763862
58.	1.	0.	-4.290446	-0.380903	2.496471
59.	1.	0.	-3.421111	-1.428820	1.394048
60.	1.	0.	-3.718932	1.598676	1.089250
61.	1.	0.	-1.477591	0.277514	2.186471
62.	1.	0.	-5.573851	-2.784639	1.139653
63.	1.	0.	-7.076543	-1.969114	0.672110
64.	1.	0.	-6.359626	-1.674503	2.263492
65.	1.	0.	-4.039538	-3.932526	-2.135665
66.	1.	0.	-5.491175	-3.604164	-1.169536
67.	1.	0.	-5.356325	-2.939208	-2.788605

Conformer **3-2**

**3-2**			Standard Orientation (Ångstroms)		
Center number	Atom number	Type	X	Y	Z
1.	6.	0.	1.913531	1.602897	-1.108047
2.	6.	0.	1.685309	0.350221	-0.175943
3.	6.	0.	0.188240	0.283828	0.280626
4.	6.	0.	-0.724966	0.982795	-0.760786
5.	6.	0.	-0.481260	2.484215	-0.858413
6.	6.	0.	1.015483	2.752169	-0.677123
7.	6.	0.	2.513115	0.374188	1.139821
8.	7.	0.	1.542843	0.844229	2.116858
9.	6.	0.	0.248382	0.860641	1.719588
10.	6.	0.	-0.507826	-1.054328	0.381589
11.	6.	0.	-1.781993	-1.029907	-0.072828
12.	6.	0.	-2.143257	0.344899	-0.651843
13.	6.	0.	3.111062	-0.998371	1.547931
14.	6.	0.	4.240295	-1.458342	0.652193
15.	6.	0.	4.041993	-2.452750	-0.314795
16.	6.	0.	5.081862	-2.855349	-1.154970
17.	6.	0.	6.341475	-2.267157	-1.041160
18.	6.	0.	6.555254	-1.277965	-0.078880
19.	6.	0.	5.514188	-0.880607	0.759348
20.	6.	0.	1.439602	3.919550	-0.184189
21.	6.	0.	3.387767	1.962304	-1.318268
22.	8.	0.	-0.686460	1.191352	2.446494
23.	8.	0.	-0.902687	2.856818	-2.183718
24.	1.	0.	-0.353480	0.587355	-1.713665

25.	1.	0.	1.953808	-0.537231	-0.756412
26.	6.	0.	-2.614216	-2.230362	-0.060447
27.	6.	0.	-3.853341	-2.448294	-0.553398
28.	6.	0.	-4.708781	-1.396681	-1.233351
29.	6.	0.	-5.382215	-0.364354	-0.284803
30.	6.	0.	-4.412480	0.350531	0.670654
31.	6.	0.	-3.265218	1.182166	0.063548
32.	8.	0.	-2.783691	2.077294	1.048592
33.	6.	0.	-6.527665	-1.000120	0.519191
34.	6.	0.	-4.466872	-3.823216	-0.447515
35.	1.	0.	-2.505839	0.204468	-1.674143
36.	1.	0.	3.337146	1.090484	1.073869
37.	1.	0.	1.539169	1.293229	-2.092767
38.	1.	0.	-1.060592	3.026843	-0.106372
39.	1.	0.	1.781161	1.103400	3.064111
40.	1.	0.	-0.046067	-1.925572	0.838190
41.	1.	0.	3.474925	-0.909814	2.579859
42.	1.	0.	2.309645	-1.744737	1.563804
43.	1.	0.	3.065820	-2.922788	-0.404468
44.	1.	0.	4.907067	-3.631049	-1.894903
45.	1.	0.	7.152266	-2.580267	-1.692157
46.	1.	0.	7.535012	-0.820166	0.023128
47.	1.	0.	5.694284	-0.117484	1.513145
48.	1.	0.	2.491102	4.170869	-0.086997
49.	1.	0.	0.731606	4.677231	0.142950
50.	1.	0.	3.483153	2.694307	-2.125819
51.	1.	0.	3.854873	2.398623	-0.430254
52.	1.	0.	3.968002	1.075577	-1.592297
53.	1.	0.	-0.786900	3.813442	-2.257461
54.	1.	0.	-2.122718	-3.084673	0.406237
55.	1.	0.	-4.116122	-0.856251	-1.975222
56.	1.	0.	-5.502039	-1.899008	-1.800434
57.	1.	0.	-5.825734	0.398399	-0.942300
58.	1.	0.	-4.992971	1.062857	1.267580
59.	1.	0.	-3.992036	-0.371247	1.381944
60.	1.	0.	-3.686881	1.831881	-0.715634
61.	1.	0.	-2.161410	1.616569	1.650804
62.	1.	0.	-6.150800	-1.749970	1.223944
63.	1.	0.	-7.255409	-1.490315	-0.137321
64.	1.	0.	-7.061063	-0.243426	1.104063
65.	1.	0.	-3.818232	-4.524870	0.084178
66.	1.	0.	-5.433293	-3.789991	0.070884
67.	1.	0.	-4.669449	-4.235590	-1.445393

Conformer **3-3**

**3-3**			Standard Orientation (Ångstroms)		
Center number	Atom number	Type	X	Y	Z
1.	6.	0.	-1.351586	2.378688	1.327099
2.	6.	0.	-1.354941	0.816655	1.242237
3.	6.	0.	-0.060298	0.275590	0.544455
4.	6.	0.	0.970683	1.410820	0.324331
5.	6.	0.	0.359535	2.427734	-0.645086
6.	6.	0.	-0.868641	3.053533	0.039110
7.	6.	0.	-2.564342	0.239993	0.440567
8.	7.	0.	-1.940496	-0.412095	-0.699544
9.	6.	0.	-0.593876	-0.359927	-0.765604
10.	6.	0.	0.806526	-0.755875	1.219392
11.	6.	0.	2.125217	-0.564377	0.987210
12.	6.	0.	2.378512	0.734725	0.201947
13.	6.	0.	-3.445441	-0.731457	1.258340
14.	6.	0.	-4.574014	-1.335007	0.450103
15.	6.	0.	-5.778530	-0.639028	0.271778
16.	6.	0.	-6.803403	-1.169093	-0.511541
17.	6.	0.	-6.641066	-2.408979	-1.132932
18.	6.	0.	-5.449245	-3.113803	-0.962484
19.	6.	0.	-4.425534	-2.580151	-0.177181
20.	6.	0.	-1.427419	4.148529	-0.481309
21.	6.	0.	-2.662228	2.939987	1.891625
22.	8.	0.	0.072654	-0.801804	-1.703462
23.	8.	0.	1.267648	3.425371	-1.084116
24.	1.	0.	1.046324	1.913628	1.293133
25.	1.	0.	-1.408275	0.440727	2.270039
26.	6.	0.	3.124369	-1.471740	1.544864
27.	6.	0.	4.471525	-1.473016	1.450299
28.	6.	0.	5.281204	-0.482403	0.637027
29.	6.	0.	5.248488	-0.709957	-0.899050
30.	6.	0.	3.838790	-0.636800	-1.511833
31.	6.	0.	3.044787	0.649605	-1.225085
32.	8.	0.	2.125202	0.893581	-2.283958
33.	6.	0.	5.934117	-2.023340	-1.302419
34.	6.	0.	5.271154	-2.524983	2.179865
35.	1.	0.	3.066816	1.345804	0.795292
36.	1.	0.	-3.203002	1.051362	0.072641
37.	1.	0.	-0.591115	2.610863	2.086027
38.	1.	0.	-0.007477	1.887474	-1.526523
39.	1.	0.	-2.474255	-0.813826	-1.458445

40.	1.	0.	0.401278	-1.606062	1.762151
41.	1.	0.	-2.805217	-1.523381	1.664341
42.	1.	0.	-3.850907	-0.174426	2.111343
43.	1.	0.	-5.915781	0.324598	0.756891
44.	1.	0.	-7.730396	-0.616229	-0.633471
45.	1.	0.	-7.439320	-2.823832	-1.741112
46.	1.	0.	-5.315704	-4.081752	-1.436882
47.	1.	0.	-3.502685	-3.138685	-0.042881
48.	1.	0.	-2.301475	4.625430	-0.050995
49.	1.	0.	-0.991968	4.617814	-1.357245
50.	1.	0.	-2.549575	3.996907	2.148079
51.	1.	0.	-3.494079	2.864380	1.184397
52.	1.	0.	-2.943901	2.407791	2.806531
53.	1.	0.	1.770178	2.972340	-1.781896
54.	1.	0.	2.683318	-2.267205	2.146228
55.	1.	0.	4.953275	0.539914	0.846879
56.	1.	0.	6.327543	-0.528937	0.962398
57.	1.	0.	5.833519	0.115383	-1.332042
58.	1.	0.	3.944824	-0.683330	-2.601392
59.	1.	0.	3.246860	-1.511971	-1.219294
60.	1.	0.	3.742524	1.495011	-1.283493
61.	1.	0.	1.429575	0.203485	-2.226696
62.	1.	0.	5.384626	-2.891183	-0.921495
63.	1.	0.	6.958843	-2.075726	-0.917765
64.	1.	0.	5.981551	-2.119108	-2.392247
65.	1.	0.	4.631601	-3.234118	2.712595
66.	1.	0.	5.908695	-3.090667	1.489040
67.	1.	0.	5.947159	-2.060542	2.910622

Conformer **3-4**

**3 -4**			Standard Orientation (Ångstroms)		
Center number	Atom number	Type	X	Y	Z
1.	6.	0.	1.924907	1.388655	-1.190035
2.	6.	0.	1.666522	0.209618	-0.186419
3.	6.	0.	0.156754	0.133017	0.220216
4.	6.	0.	-0.721665	0.990752	-0.724890
5.	6.	0.	-0.358249	2.462535	-0.508084
6.	6.	0.	1.119237	2.655410	-0.890610
7.	6.	0.	2.461234	0.301259	1.152270
8.	7.	0.	1.438113	0.677753	2.117729
9.	6.	0.	0.157341	0.617983	1.692079

10.	6.	0.	-0.578754	-1.181391	0.174807
11.	6.	0.	-1.854392	-1.057847	-0.256186
12.	6.	0.	-2.167220	0.384911	-0.686253
13.	6.	0.	3.160359	-1.018429	1.572267
14.	6.	0.	4.354264	-1.379734	0.716183
15.	6.	0.	4.269093	-2.371512	-0.269695
16.	6.	0.	5.367945	-2.680950	-1.073766
17.	6.	0.	6.574195	-2.001937	-0.902096
18.	6.	0.	6.675606	-1.015448	0.081263
19.	6.	0.	5.575909	-0.710470	0.882094
20.	6.	0.	1.615145	3.891204	-0.986279
21.	6.	0.	3.415807	1.630465	-1.454131
22.	8.	0.	-0.821538	0.871973	2.395821
23.	8.	0.	-1.196533	3.367744	-1.212929
24.	1.	0.	-0.387665	0.732552	-1.734600
25.	1.	0.	1.965848	-0.706072	-0.704874
26.	6.	0.	-2.721994	-2.227049	-0.373122
27.	6.	0.	-3.996056	-2.345048	-0.805069
28.	6.	0.	-4.871640	-1.185233	-1.236286
29.	6.	0.	-5.428400	-0.314735	-0.077685
30.	6.	0.	-4.341664	0.322972	0.805644
31.	6.	0.	-3.289678	1.182658	0.082072
32.	8.	0.	-2.771270	2.156233	0.980960
33.	6.	0.	-6.430659	-1.087134	0.792672
34.	6.	0.	-4.638711	-3.708997	-0.872983
35.	1.	0.	-2.513589	0.349232	-1.724337
36.	1.	0.	3.219155	1.089739	1.104732
37.	1.	0.	1.523651	1.013073	-2.142503
38.	1.	0.	-0.439596	2.686976	0.560527
39.	1.	0.	1.640561	0.924739	3.076954
40.	1.	0.	-0.143064	-2.115458	0.519993
41.	1.	0.	3.480690	-0.909919	2.616673
42.	1.	0.	2.420130	-1.826577	1.556430
43.	1.	0.	3.336265	-2.913733	-0.402265
44.	1.	0.	5.281329	-3.455819	-1.829968
45.	1.	0.	7.430752	-2.242810	-1.524564
46.	1.	0.	7.613307	-0.487278	0.228060
47.	1.	0.	5.667874	0.052812	1.651482
48.	1.	0.	2.649835	4.091914	-1.241296
49.	1.	0.	0.969538	4.750072	-0.834925
50.	1.	0.	3.549131	2.254954	-2.341934
51.	1.	0.	3.913260	2.138680	-0.622230
52.	1.	0.	3.935655	0.683865	-1.628179
53.	1.	0.	-1.975674	3.441719	-0.637916

54.	1.	0.	-2.227357	-3.151476	-0.073493
55.	1.	0.	-4.329447	-0.538249	-1.931729
56.	1.	0.	-5.724758	-1.577648	-1.803190
57.	1.	0.	-5.976638	0.507131	-0.562530
58.	1.	0.	-4.837995	0.998597	1.511091
59.	1.	0.	-3.842086	-0.443237	1.410663
60.	1.	0.	-3.810206	1.777852	-0.679762
61.	1.	0.	-2.187011	1.692423	1.619181
62.	1.	0.	-5.941767	-1.906767	1.330875
63.	1.	0.	-7.240228	-1.513568	0.189864
64.	1.	0.	-6.883325	-0.429048	1.541831
65.	1.	0.	-3.974949	-4.496473	-0.505690
66.	1.	0.	-5.566620	-3.742807	-0.288594
67.	1.	0.	-4.918502	-3.954426	-1.906573

Conformer **3-5**

**3-5**			Standard Orientation (Ångstroms)		
Center number	Atom number	Type	X	Y	Z
1.	6.	0.	-2.341322	-2.085303	-1.269268
2.	6.	0.	-2.185532	-0.654387	-0.631153
3.	6.	0.	-0.751981	-0.482065	-0.010553
4.	6.	0.	0.235541	-1.500934	-0.639579
5.	6.	0.	-0.117205	-2.951005	-0.334651
6.	6.	0.	-1.642177	-3.103472	-0.383504
7.	6.	0.	-3.195844	-0.404030	0.529593
8.	7.	0.	-2.351864	-0.398088	1.711452
9.	6.	0.	-1.021807	-0.558756	1.513333
10.	6.	0.	0.023295	0.797675	-0.235239
11.	6.	0.	1.342508	0.585570	-0.448783
12.	6.	0.	1.672080	-0.912241	-0.511903
13.	6.	0.	-4.077529	0.866974	0.396803
14.	6.	0.	-3.349868	2.181093	0.220916
15.	6.	0.	-2.780310	2.844765	1.317903
16.	6.	0.	-2.090750	4.045667	1.150592
17.	6.	0.	-1.963132	4.611187	-0.119877
18.	6.	0.	-2.536107	3.970059	-1.218755
19.	6.	0.	-3.224081	2.767975	-1.046253
20.	6.	0.	-2.256805	-4.057241	0.322782
21.	6.	0.	-3.776645	-2.431722	-1.679749
22.	8.	0.	-0.205537	-0.643359	2.428865
23.	8.	0.	0.496711	-3.725432	-1.381177

24.	1.	0.	0.035990	-1.397317	-1.712203
25.	1.	0.	-2.344333	0.084149	-1.422648
26.	6.	0.	2.258630	1.700883	-0.675137
27.	6.	0.	3.568741	1.711578	-1.005441
28.	6.	0.	4.423768	0.471262	-1.185728
29.	6.	0.	4.874932	-0.227810	0.127741
30.	6.	0.	3.726690	-0.581845	1.088173
31.	6.	0.	2.616810	-1.524586	0.584577
32.	8.	0.	1.934146	-2.063636	1.702000
33.	6.	0.	5.934959	0.599808	0.872109
34.	6.	0.	4.272819	3.028594	-1.226440
35.	1.	0.	2.178193	-1.114068	-1.460401
36.	1.	0.	-3.892177	-1.246150	0.607712
37.	1.	0.	-1.769207	-2.054945	-2.205001
38.	1.	0.	0.269529	-3.255194	0.642194
39.	1.	0.	-2.717637	-0.419457	2.653478
40.	1.	0.	-0.433881	1.778164	-0.153963
41.	1.	0.	-4.750142	0.701849	-0.452961
42.	1.	0.	-4.718114	0.917622	1.287163
43.	1.	0.	-2.876321	2.416791	2.311445
44.	1.	0.	-1.655864	4.541388	2.013576
45.	1.	0.	-1.429552	5.548050	-0.249926
46.	1.	0.	-2.452233	4.406276	-2.209912
47.	1.	0.	-3.677033	2.281563	-1.906792
48.	1.	0.	-3.327692	-4.228828	0.272168
49.	1.	0.	-1.695074	-4.714813	0.982060
50.	1.	0.	-3.789278	-3.368364	-2.244986
51.	1.	0.	-4.455462	-2.558003	-0.831050
52.	1.	0.	-4.192936	-1.647919	-2.322082
53.	1.	0.	0.317319	-4.655018	-1.187426
54.	1.	0.	1.775299	2.673465	-0.578857
55.	1.	0.	3.898764	-0.256604	-1.809247
56.	1.	0.	5.325817	0.742702	-1.748023
57.	1.	0.	5.351300	-1.169765	-0.183187
58.	1.	0.	4.160224	-1.091059	1.956325
59.	1.	0.	3.266744	0.337348	1.471781
60.	1.	0.	3.092064	-2.393279	0.108999
61.	1.	0.	1.296204	-1.404690	2.049348
62.	1.	0.	5.515912	1.544562	1.236217
63.	1.	0.	6.787728	0.835724	0.225601
64.	1.	0.	6.314983	0.054806	1.742513
65.	1.	0.	3.614493	3.883125	-1.046927
66.	1.	0.	5.148146	3.127725	-0.572136
67.	1.	0.	4.650644	3.100034	-2.255584

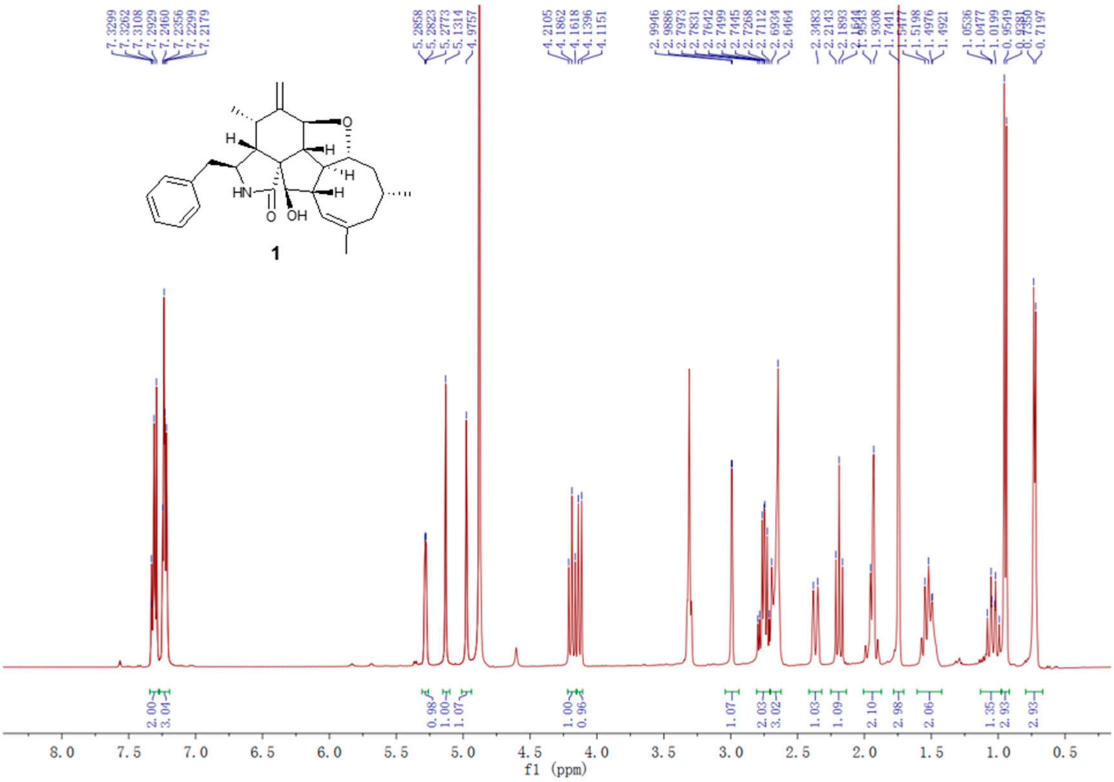

Figure S1. ^{1}H-NMR of phomoparagin A (**1**)

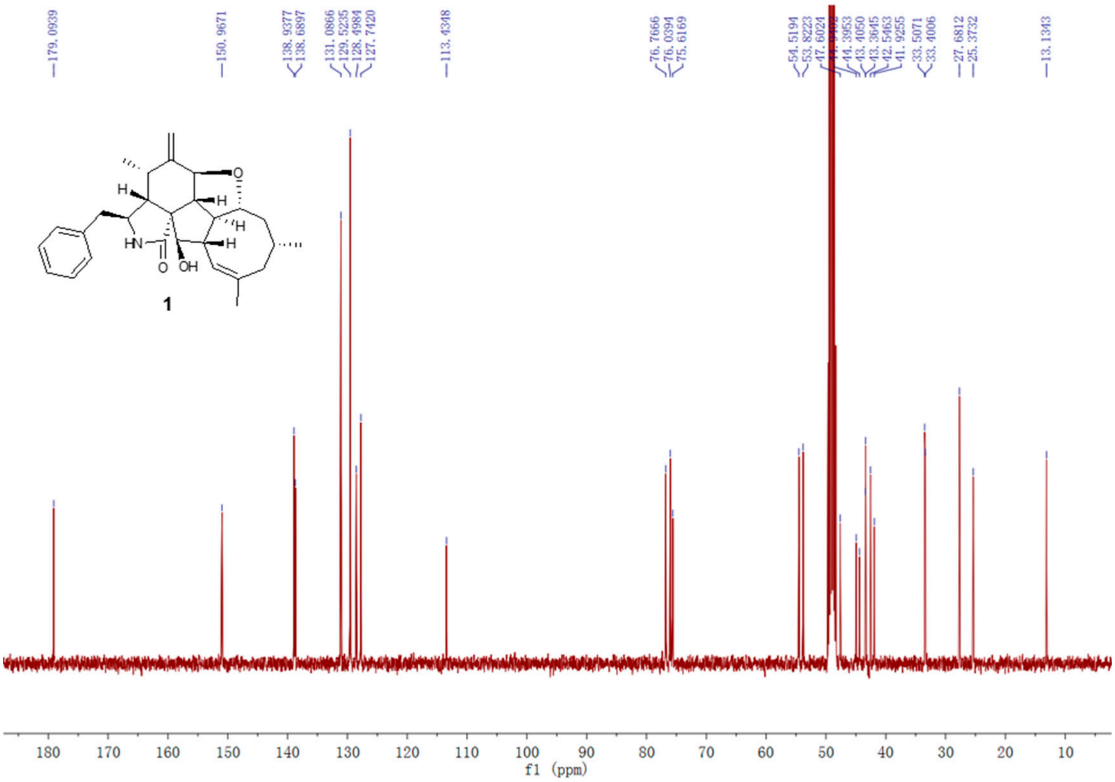

Figure S2. ^{13}C-NMR of phomoparagin A (**1**)

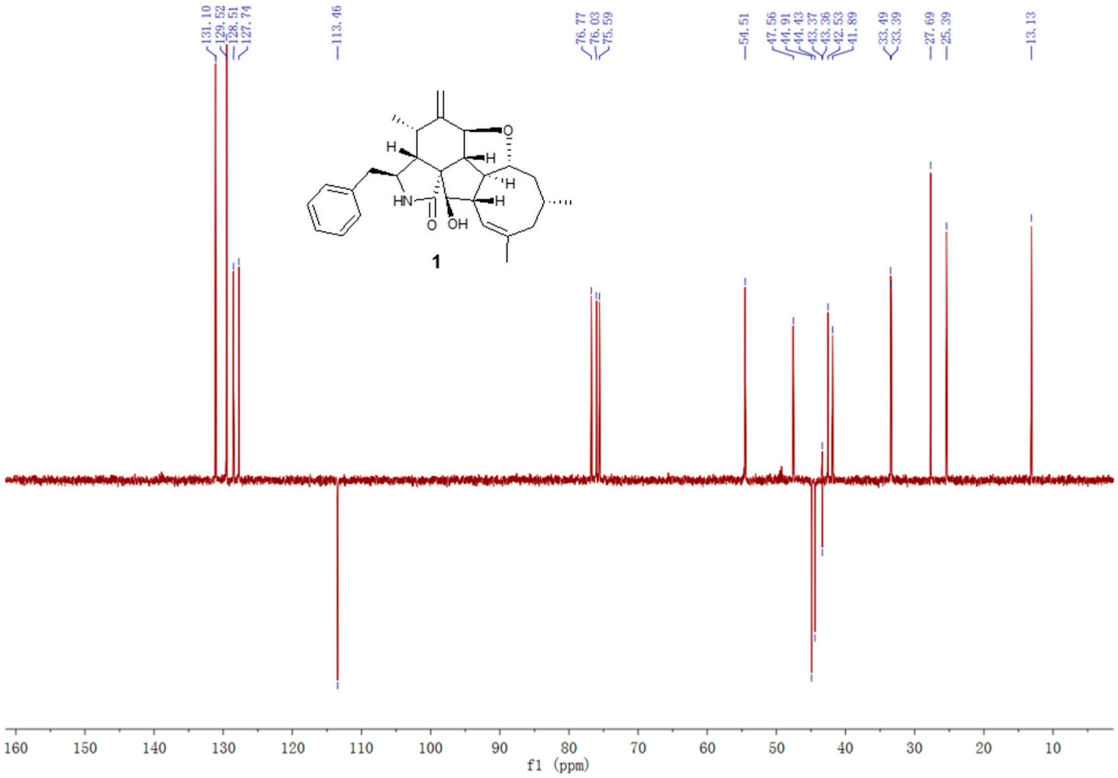

Figure S3. DEPT of phomoparagin A (**1**)

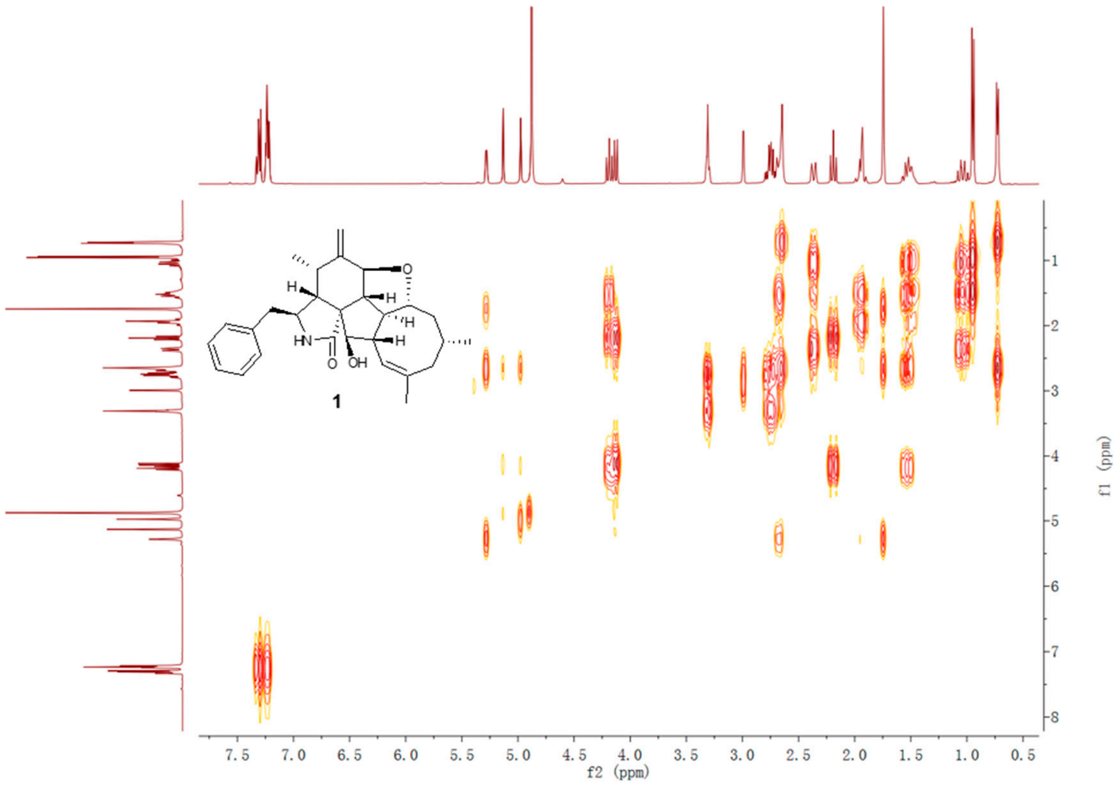

Figure S4. ^{1}H-^{1}H COSY of phomoparagin A (**1**)

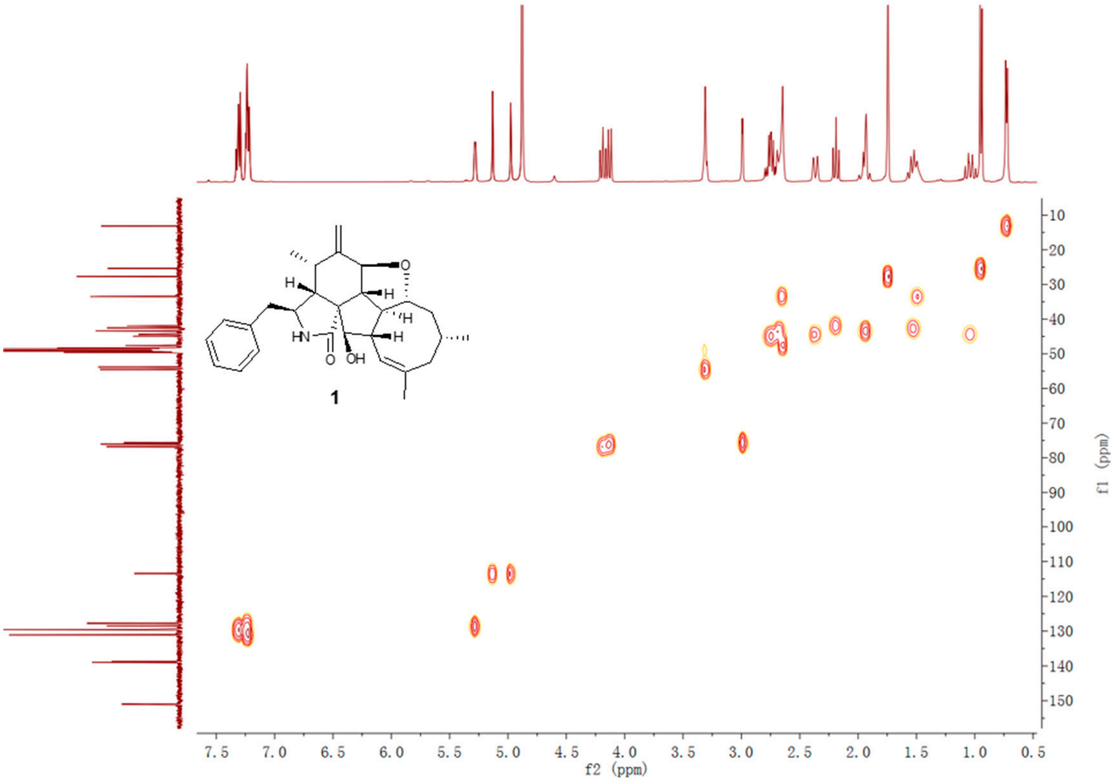

Figure S5. HMQC of phomoparagin A (**1**)

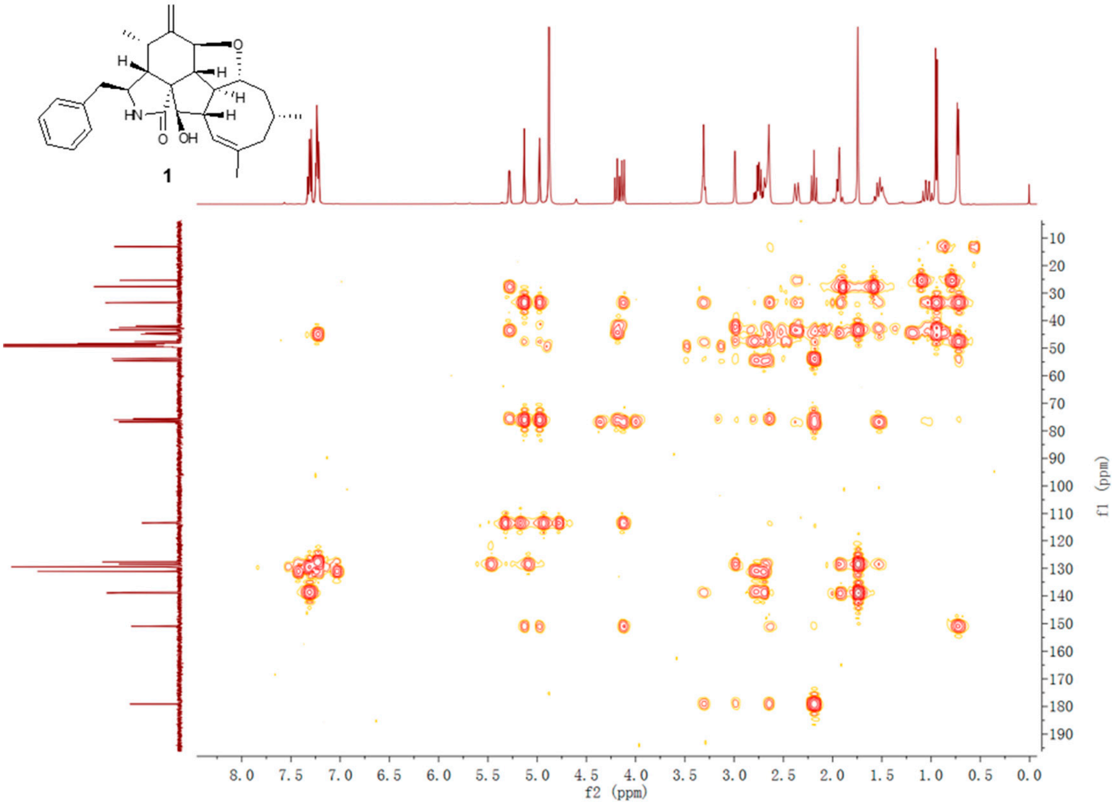

Figure S6. HMBC of phomoparagin A (**1**)

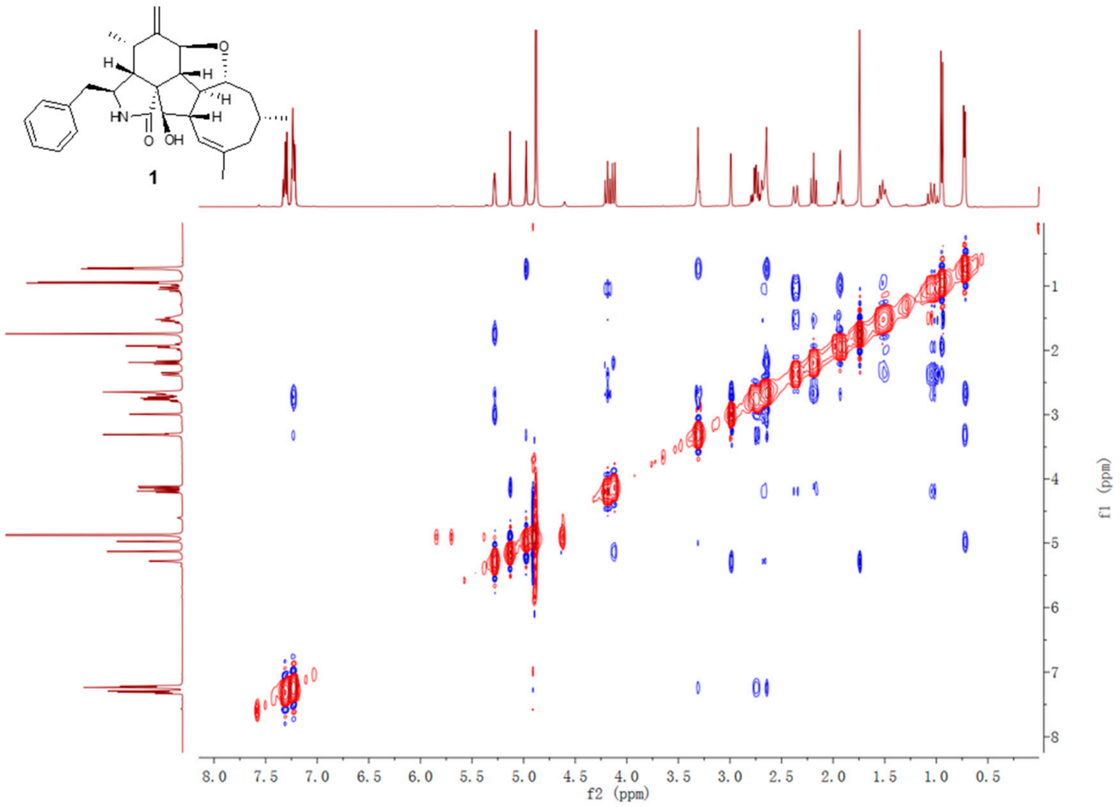

Figure S7. NOESY of phomoparagin A (**1**)

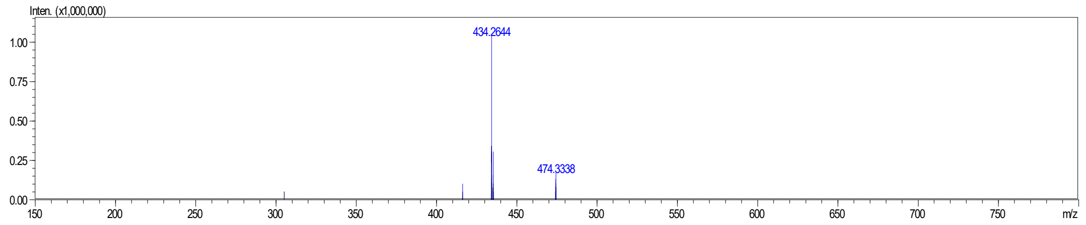

Figure S8. HR-ESI-MS of phomoparagin A (**1**)

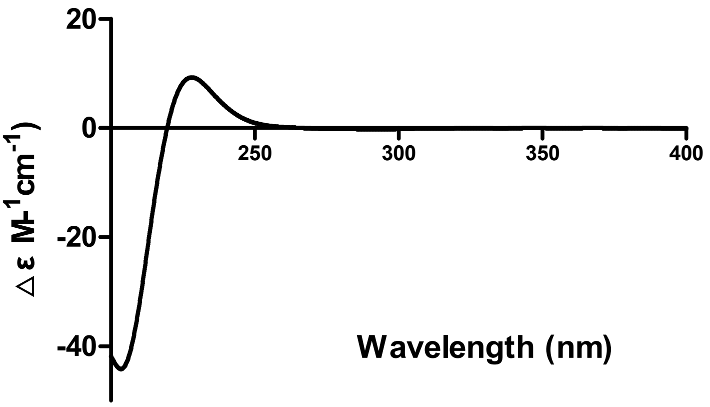

Figure S9 CD spectrum of phomoparagin A (**1**)

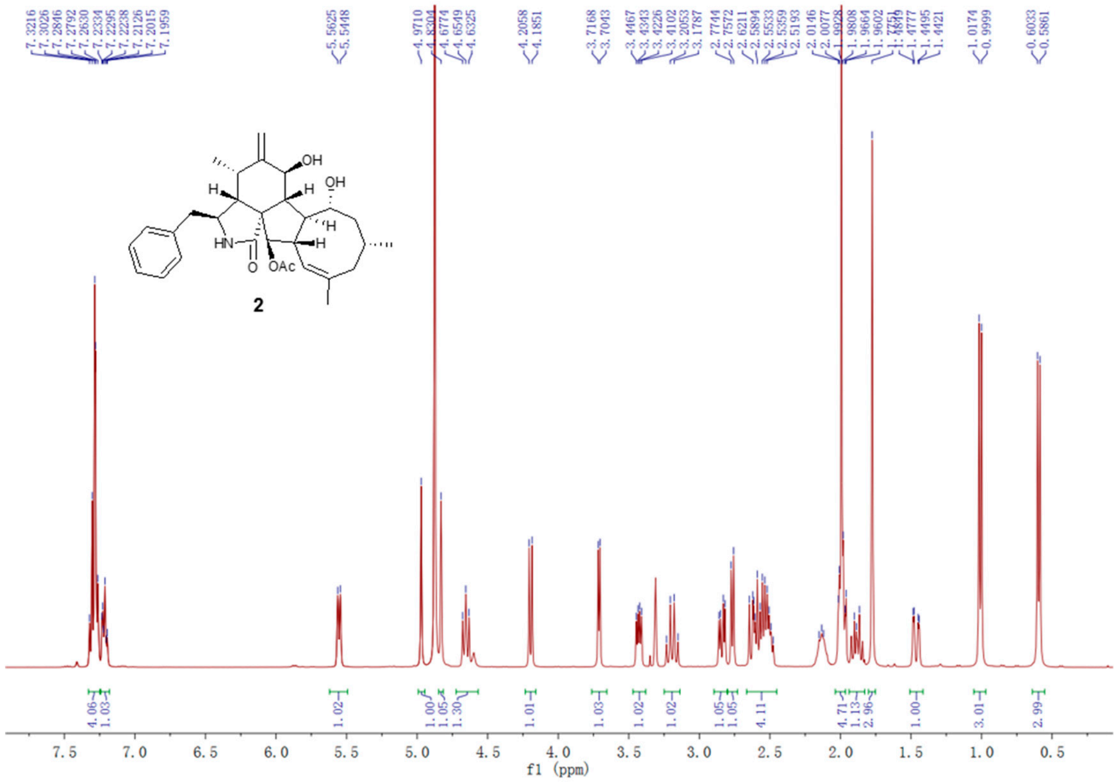

Figure S10. ^{1}H-NMR of phomoparagin B (**2**)

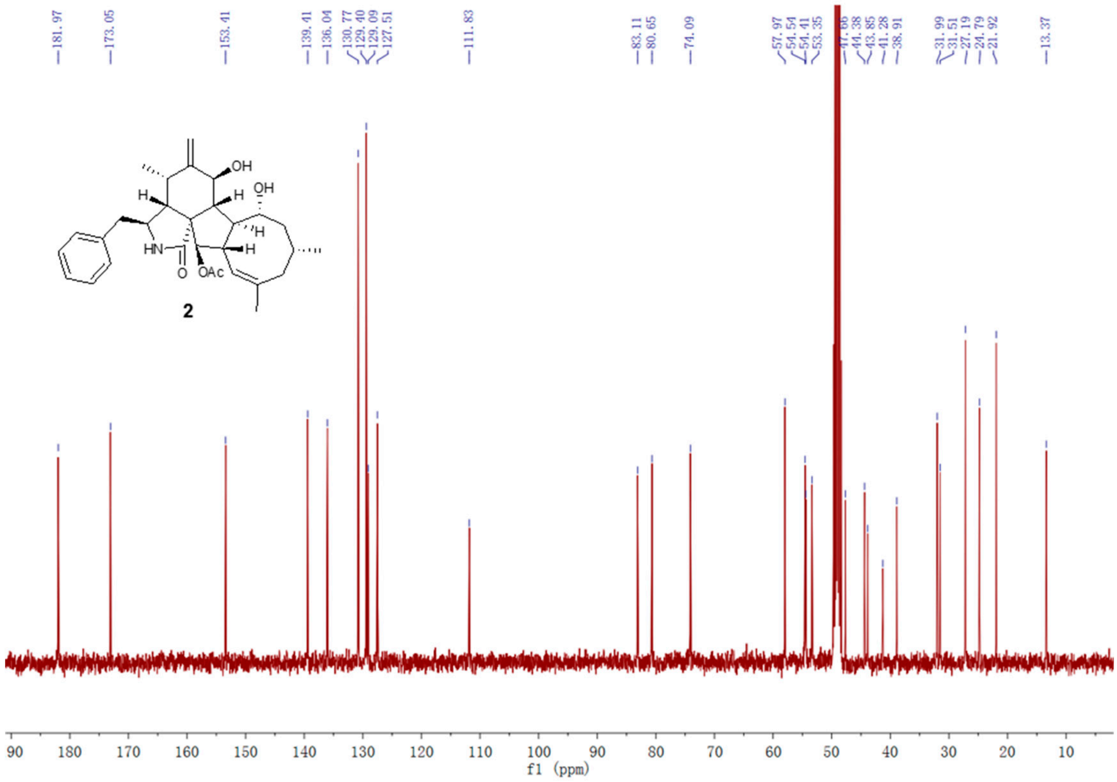

Figure S11. ^{13}C-NMR of phomoparagin B (**2**)

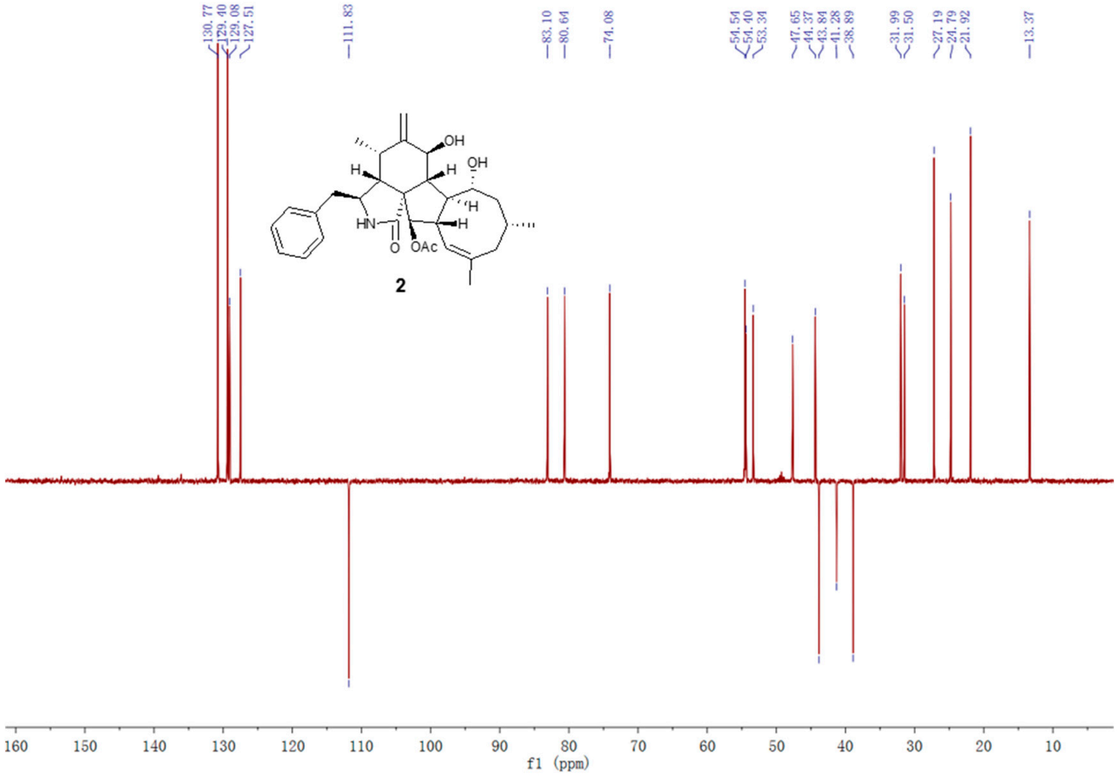

Figure S12. DEPT of phomoparagin B (**2**)

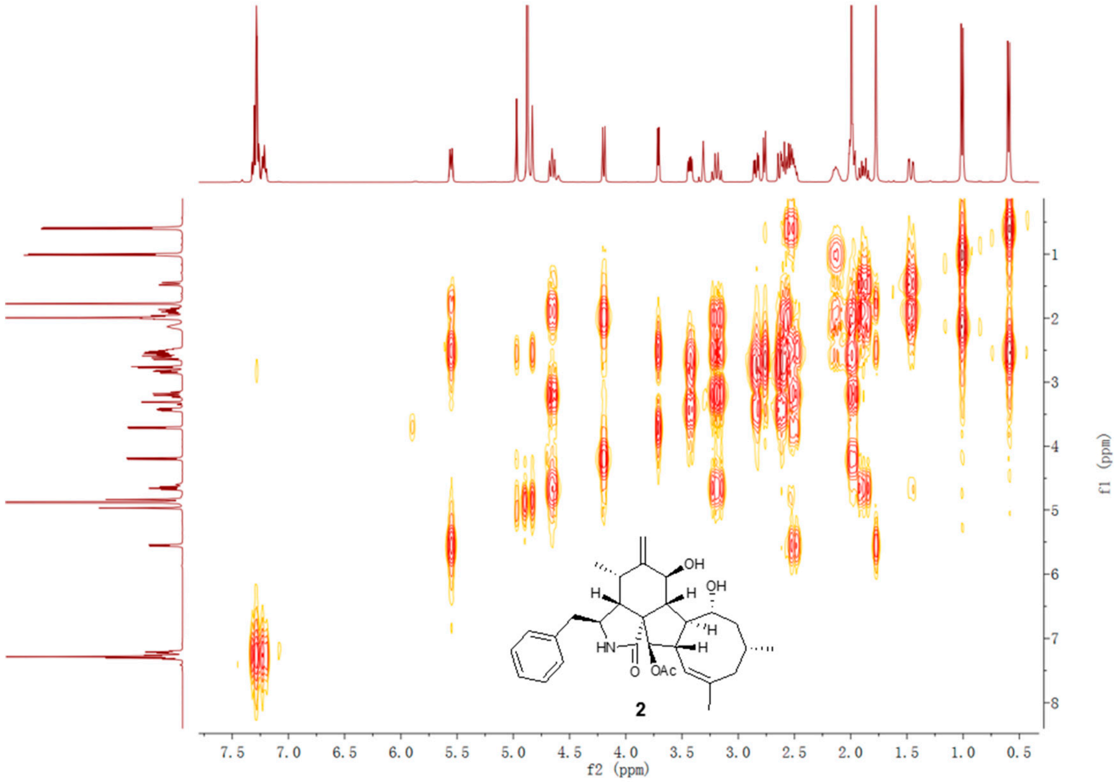

Figure S13. ^{1}H-^{1}H COSY of phomoparagin B (**2**)

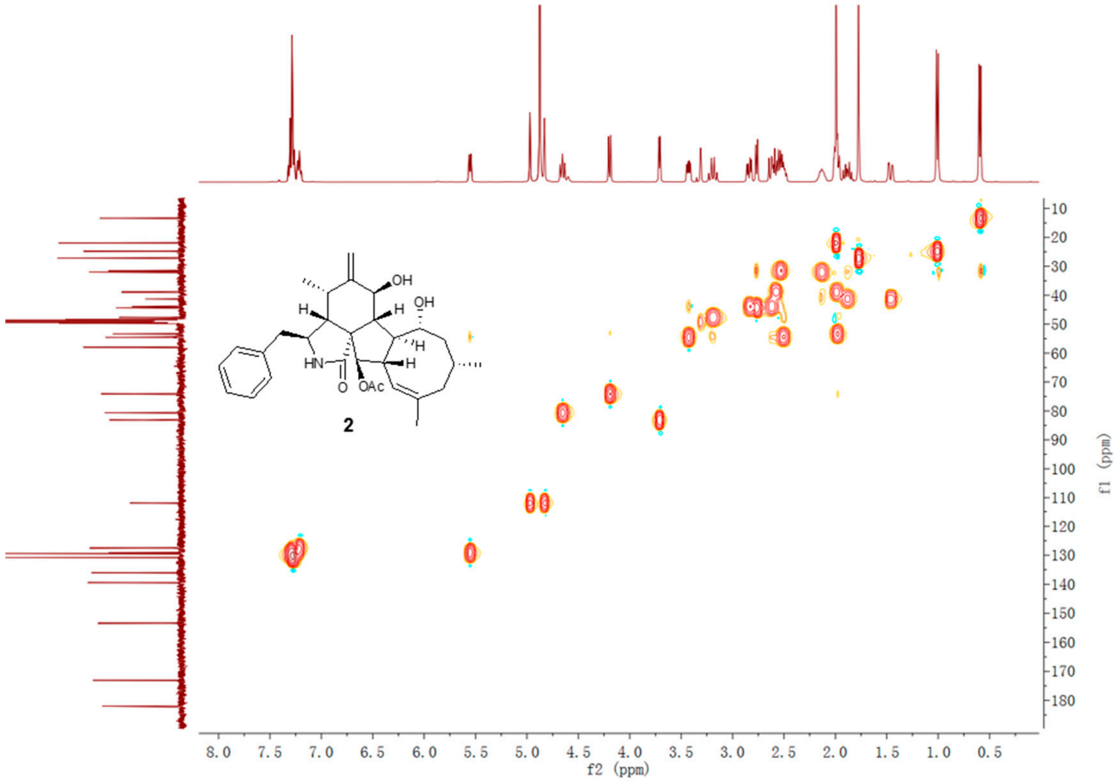

Figure S14. HMQC of phomoparagin B (**2**)

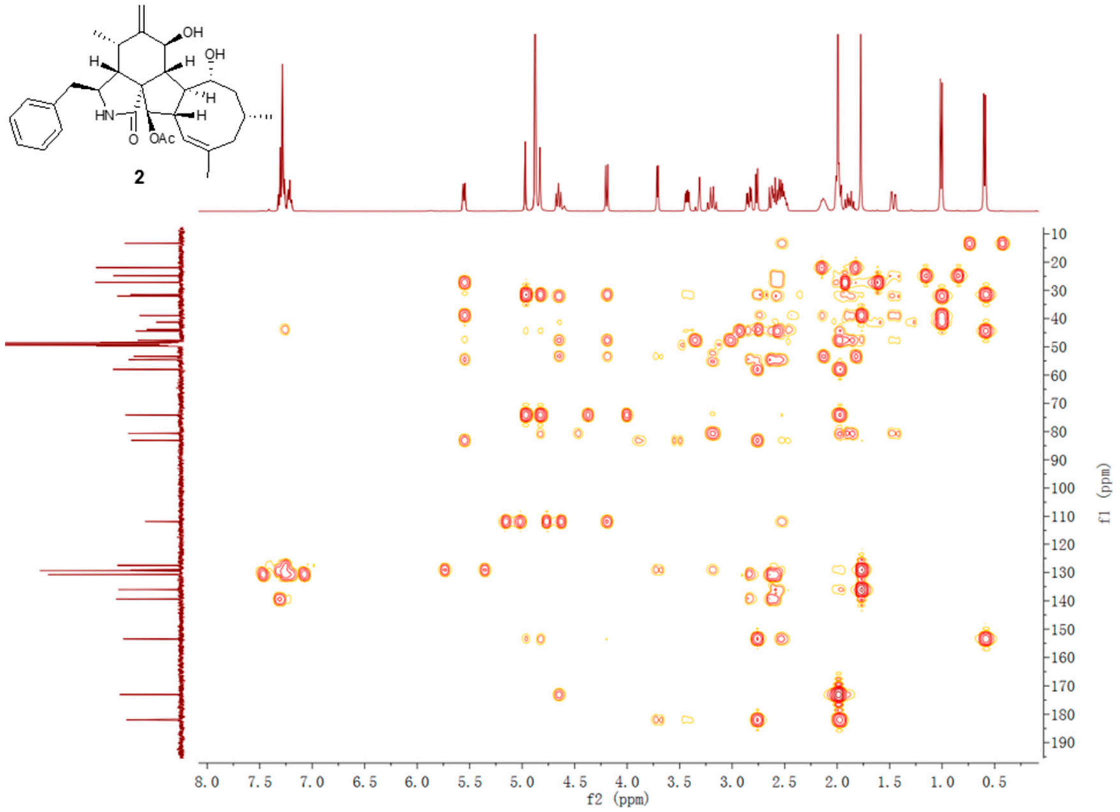

Figure S15. HMBC of phomoparagin B (**2**)

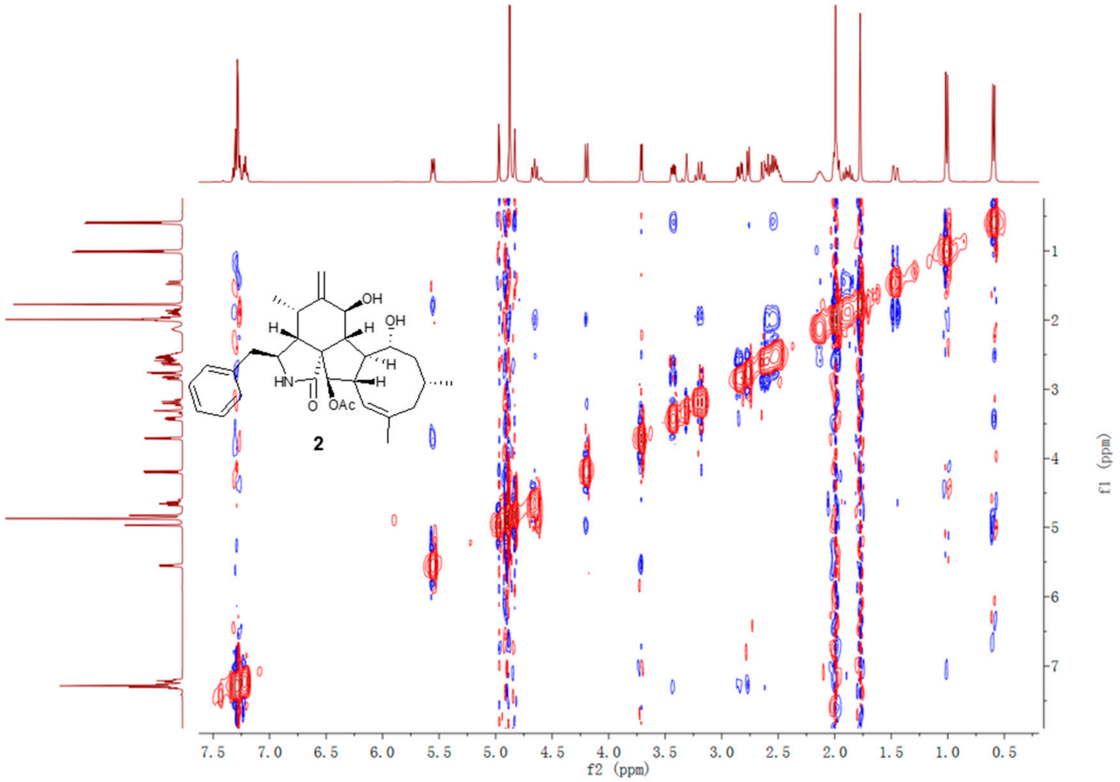

Figure S16. NOESY of phomoparagin B (**2**)

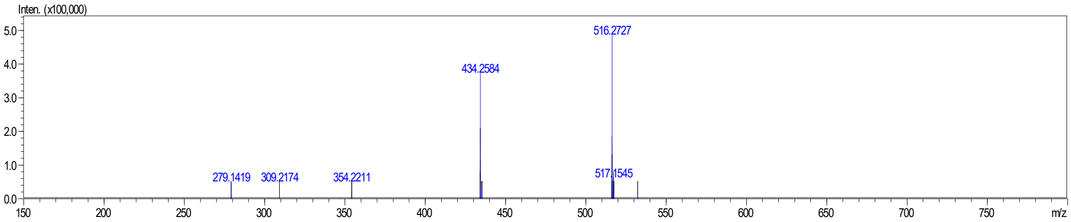

Figure S17. HR-ESI-MS of phomoparagin B (**2**)

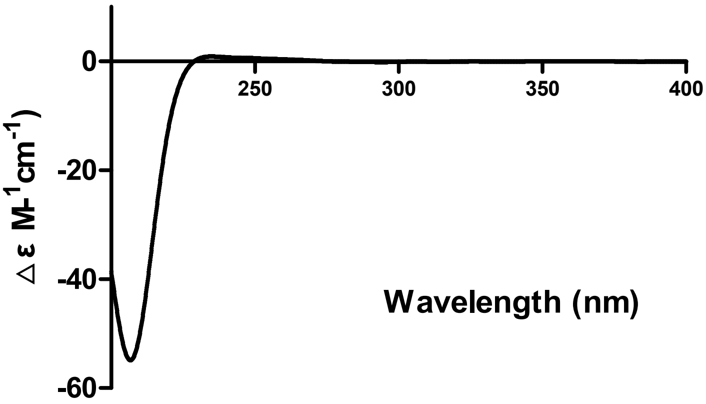

Figure S18. CD spectrum of phomoparagin B (**2**)

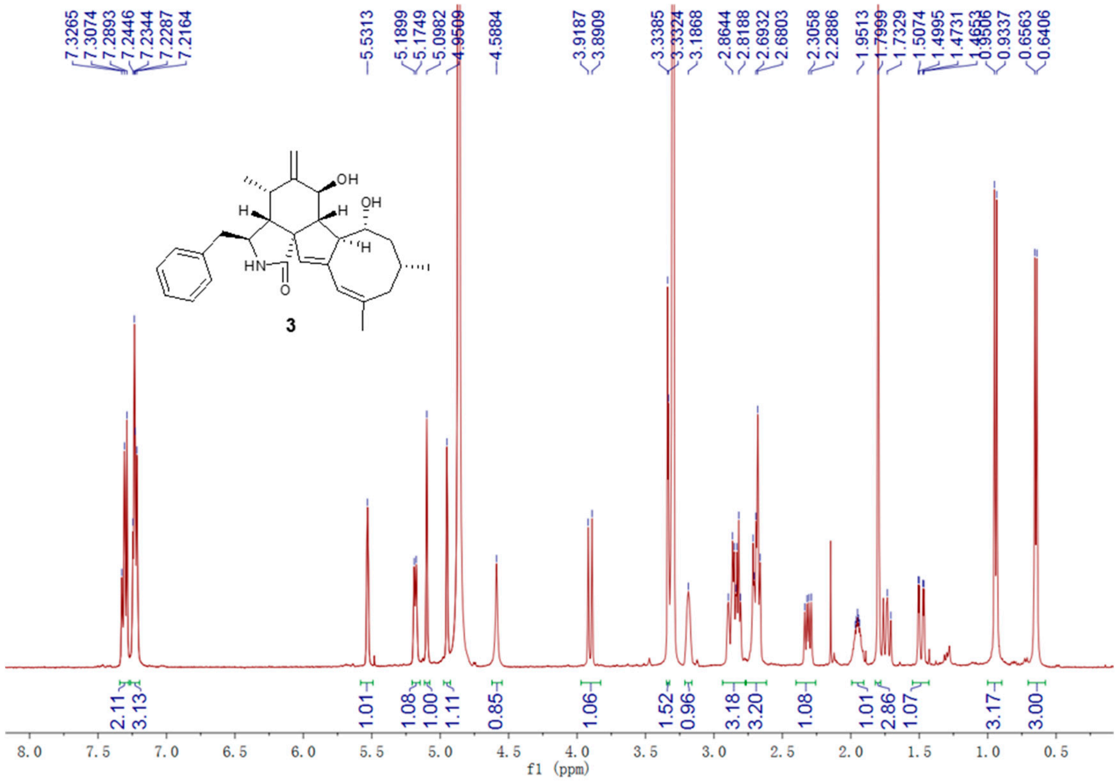

Figure S19. ^{1}H-NMR of phomoparagin C (**3**)

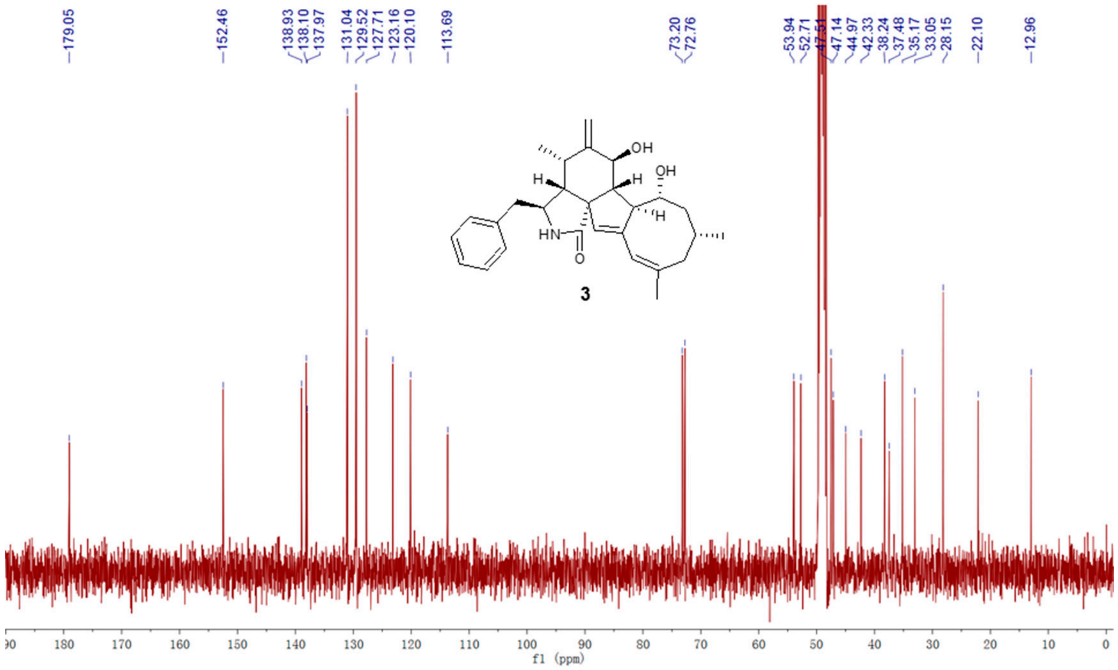

Figure S20. ^{13}C-NMR of phomoparagin C (**3**)

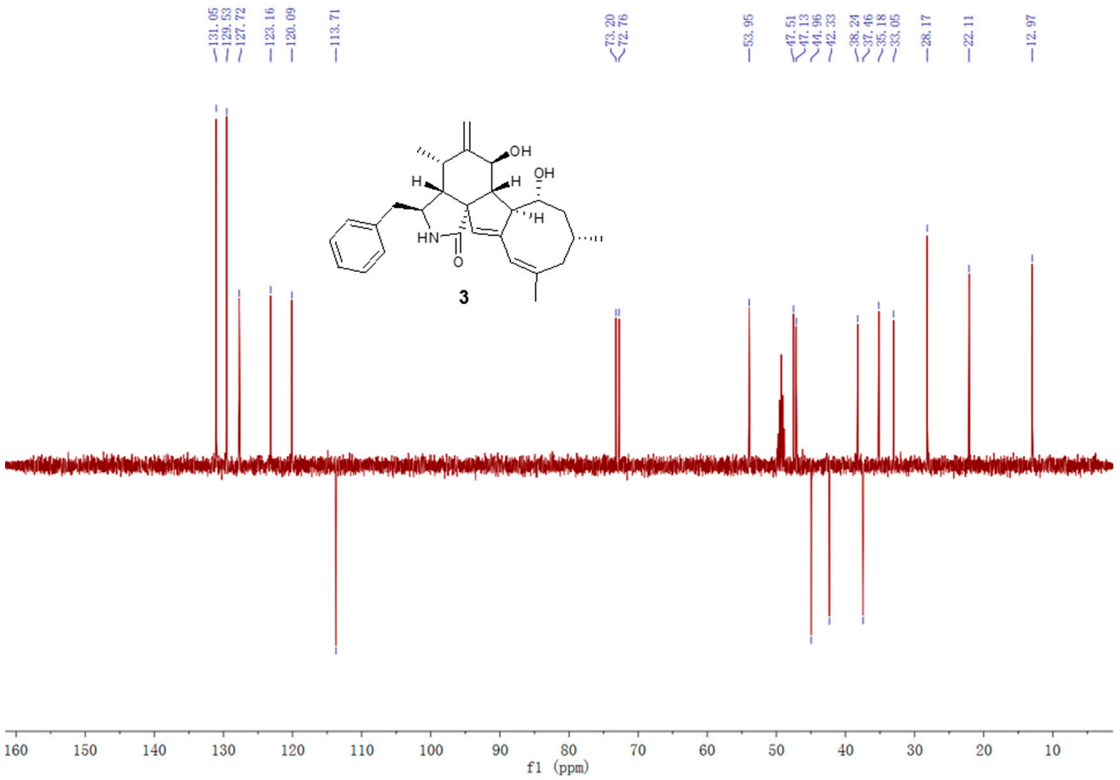

Figure S21. DEPT of phomoparagin C (**3**)

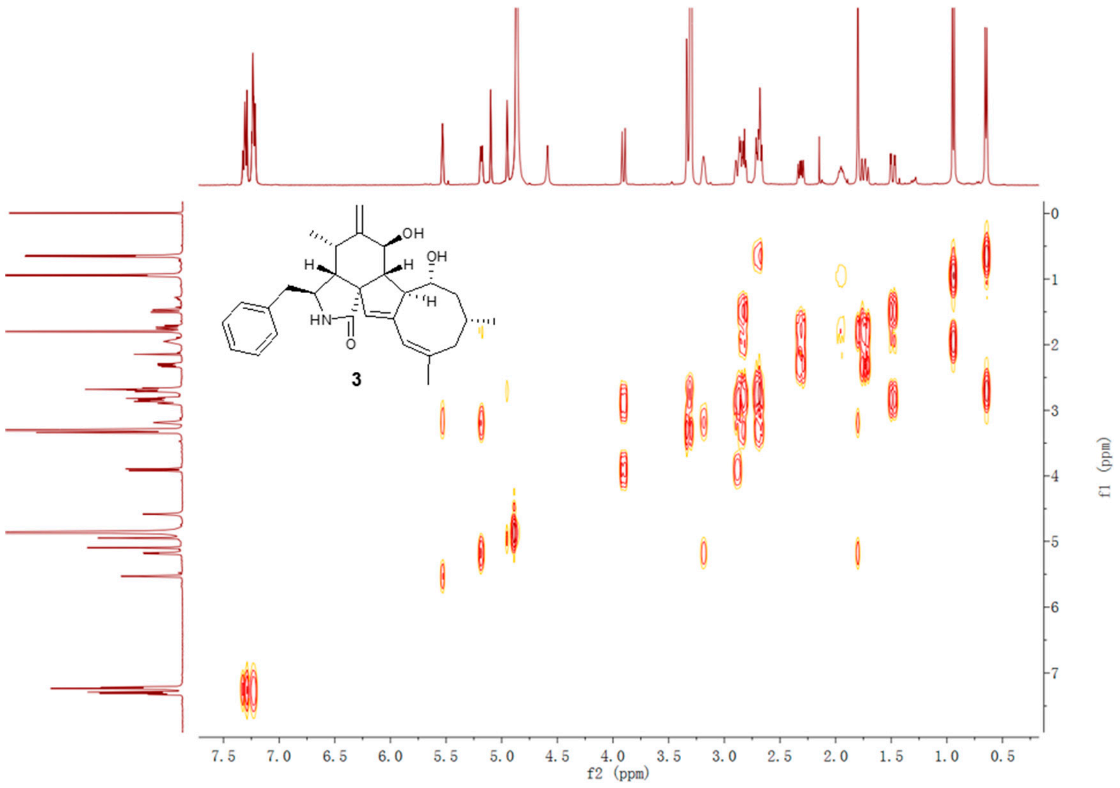

Figure S22. ^{1}H-^{1}H COSY of phomoparagin C (**3**)

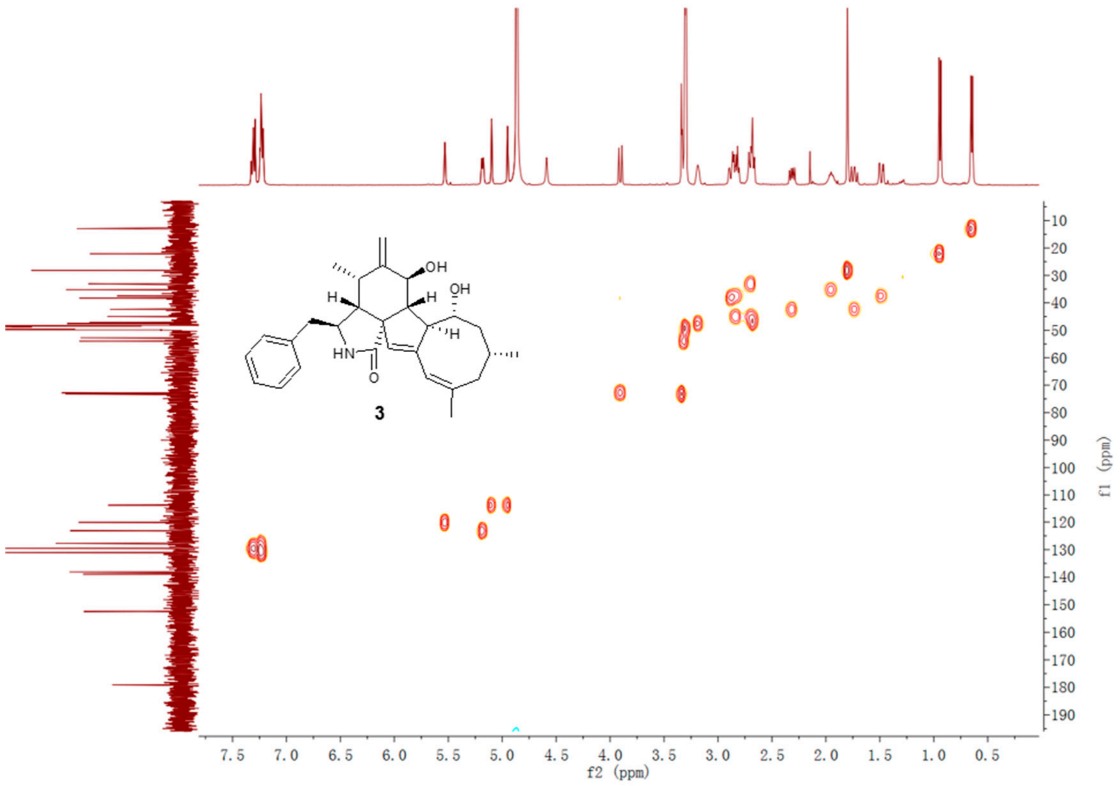

Figure S23. HMQC of phomoparagin C (**3**)

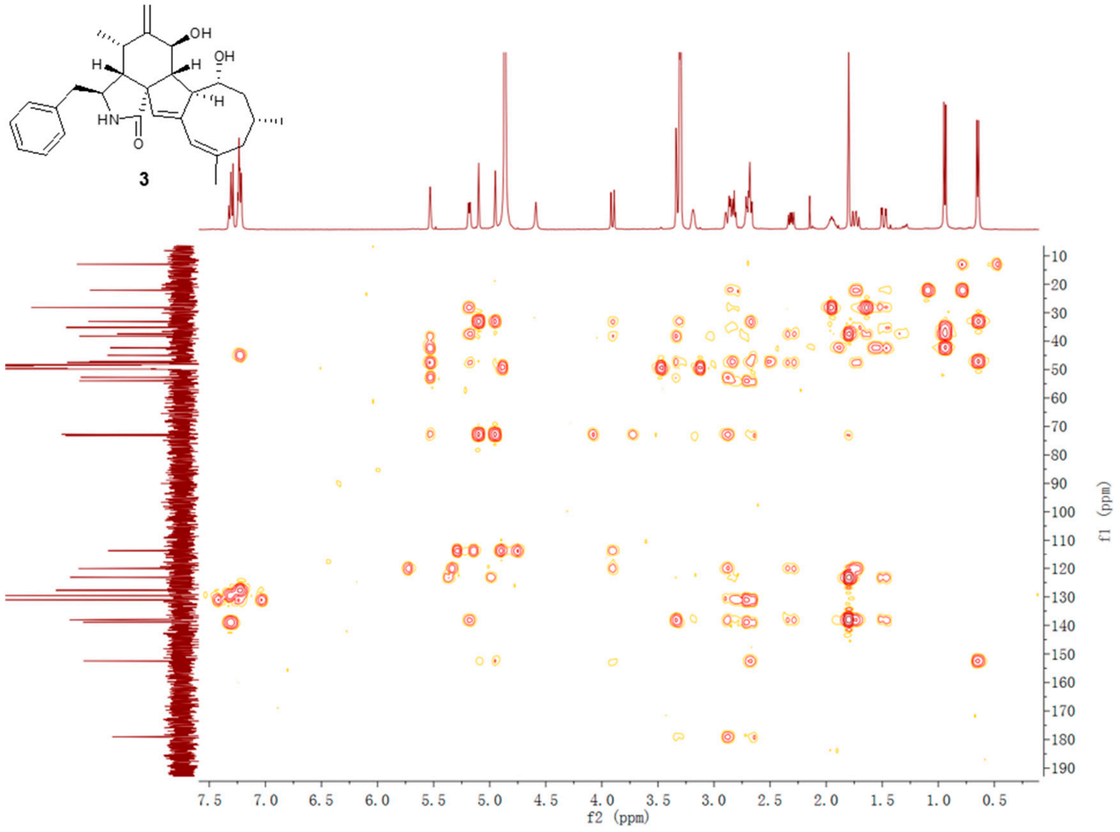

Figure S24. HMBC of phomoparagin C (**3**)

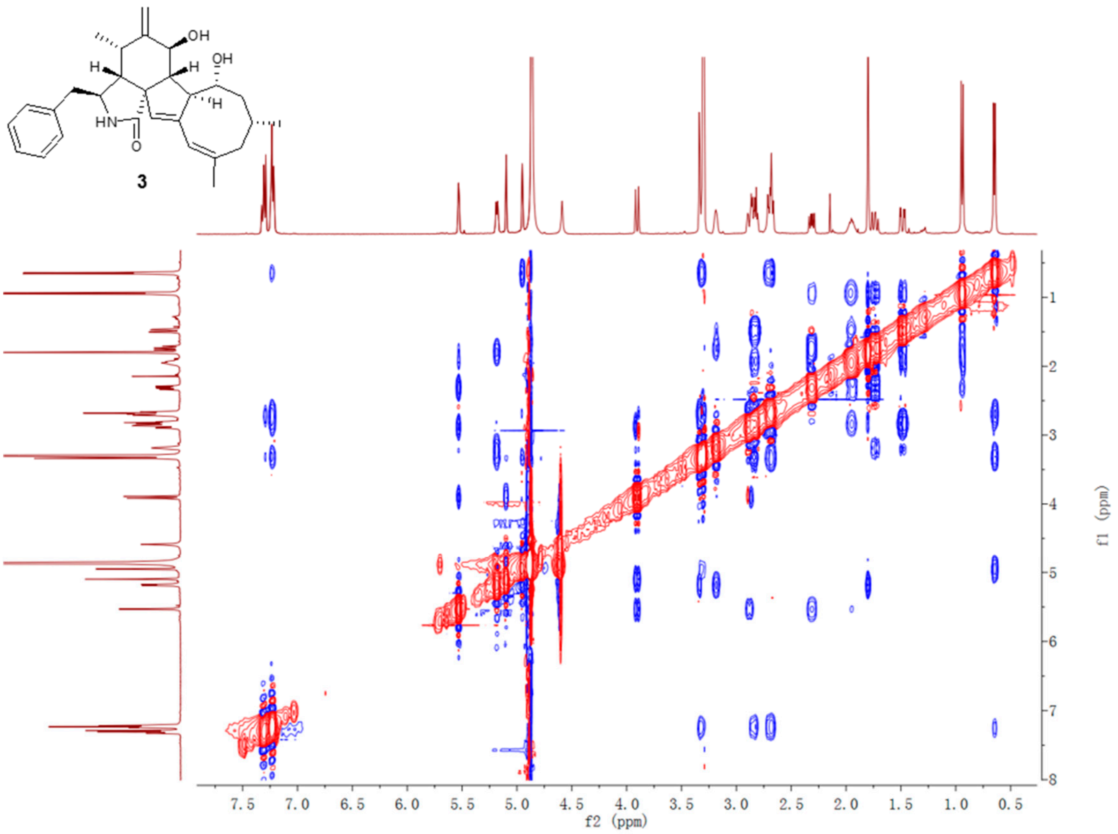

Figure S25. NOESY of phomoparagin C (**3**)

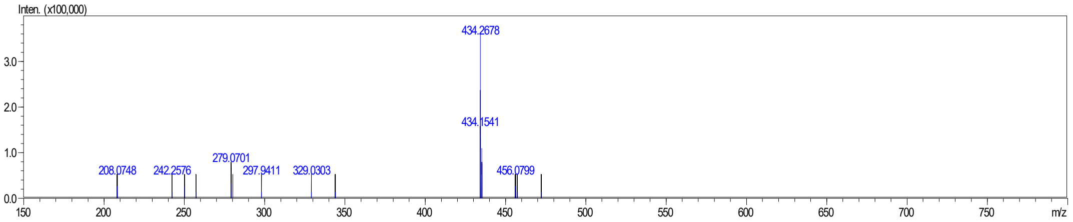

Figure S26. HR-ESI-MS of phomoparagin C (**3**)

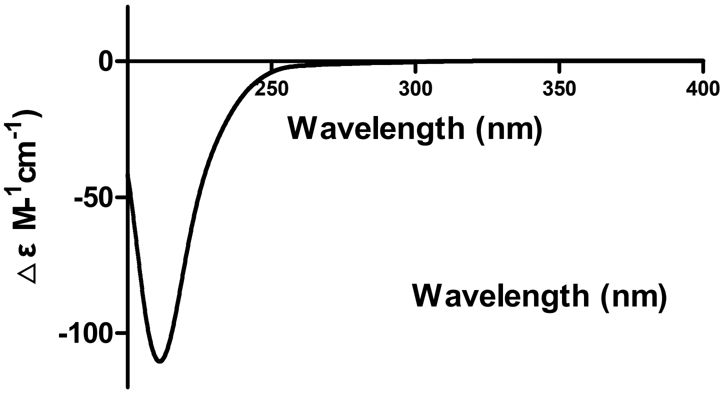

Figure S27. CD spectrum of phomoparagin C (**3**)

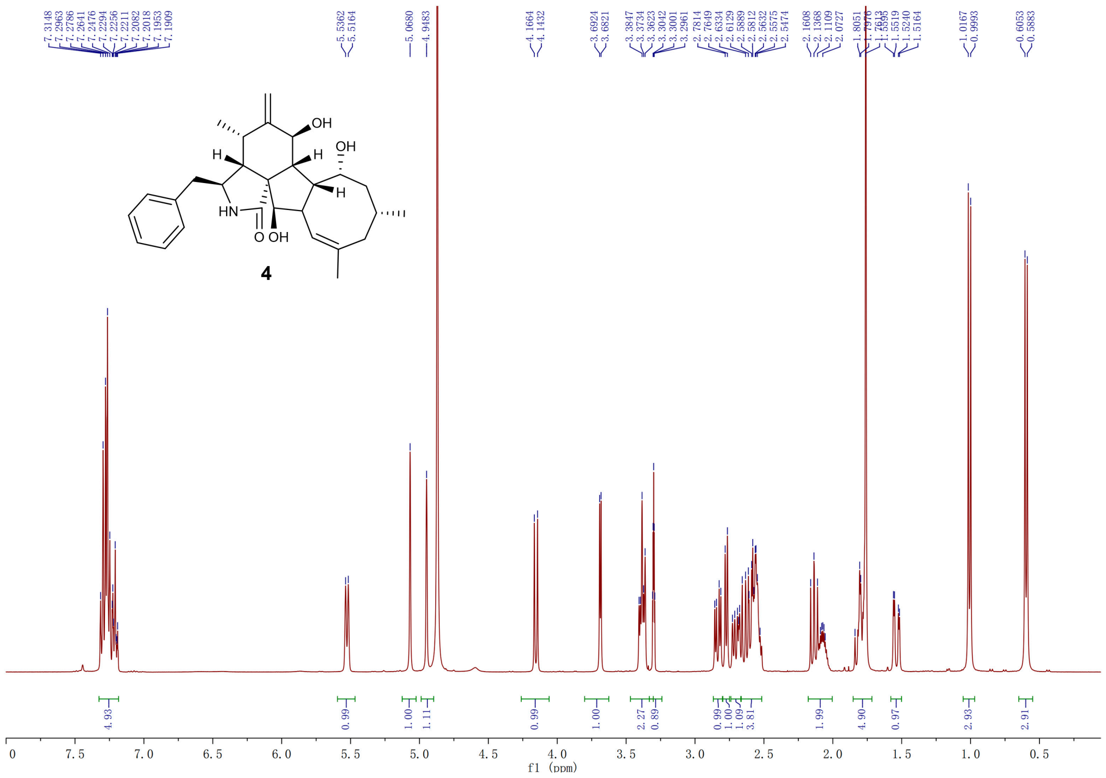

Figure S28. ^{1}H-NMR of phomopchalasin A (**4**)

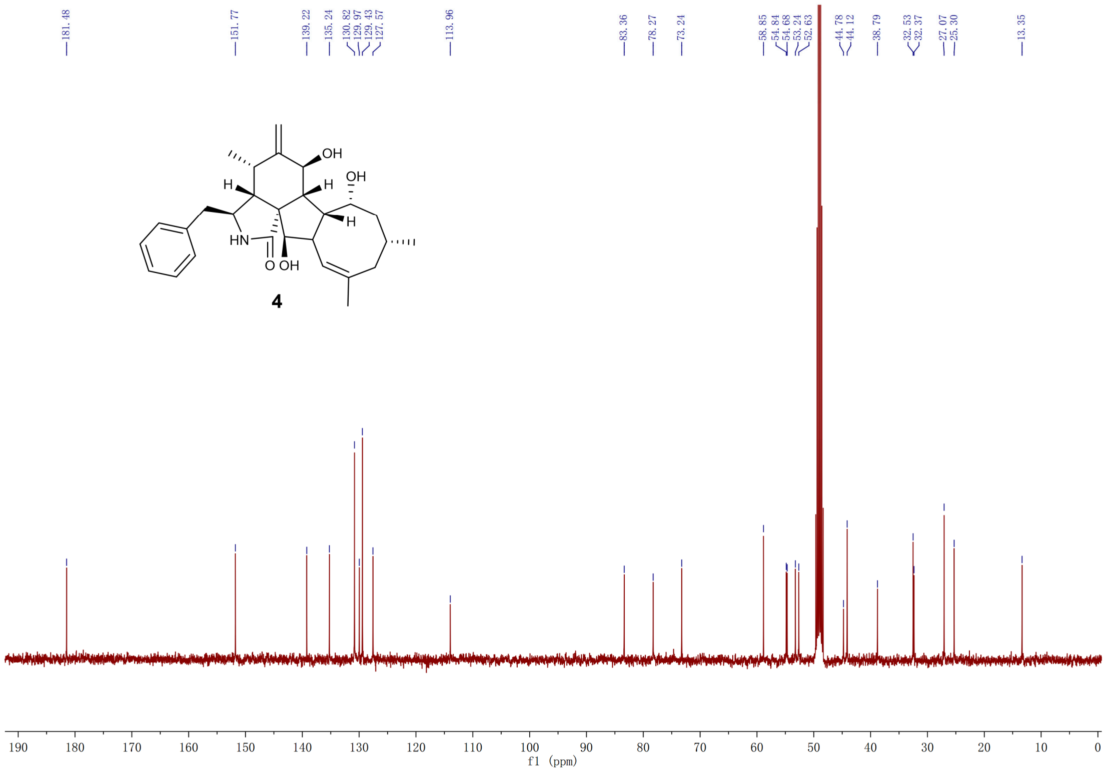

Figure S29. ^{13}C-NMR of phomopchalasin A (**4**)

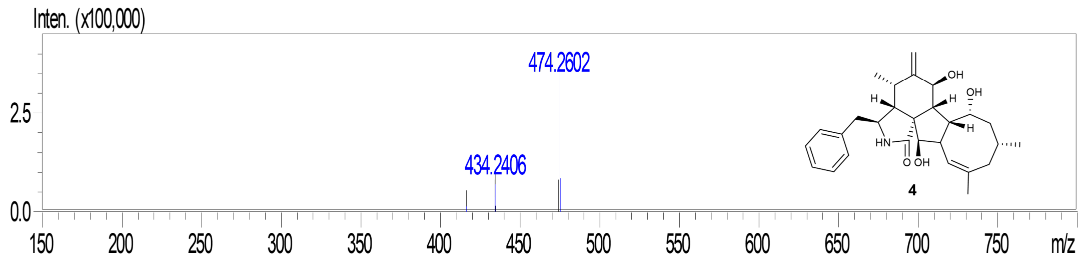

Figure S30. HR-ESI-MS of phomopchalasin A (**4**)

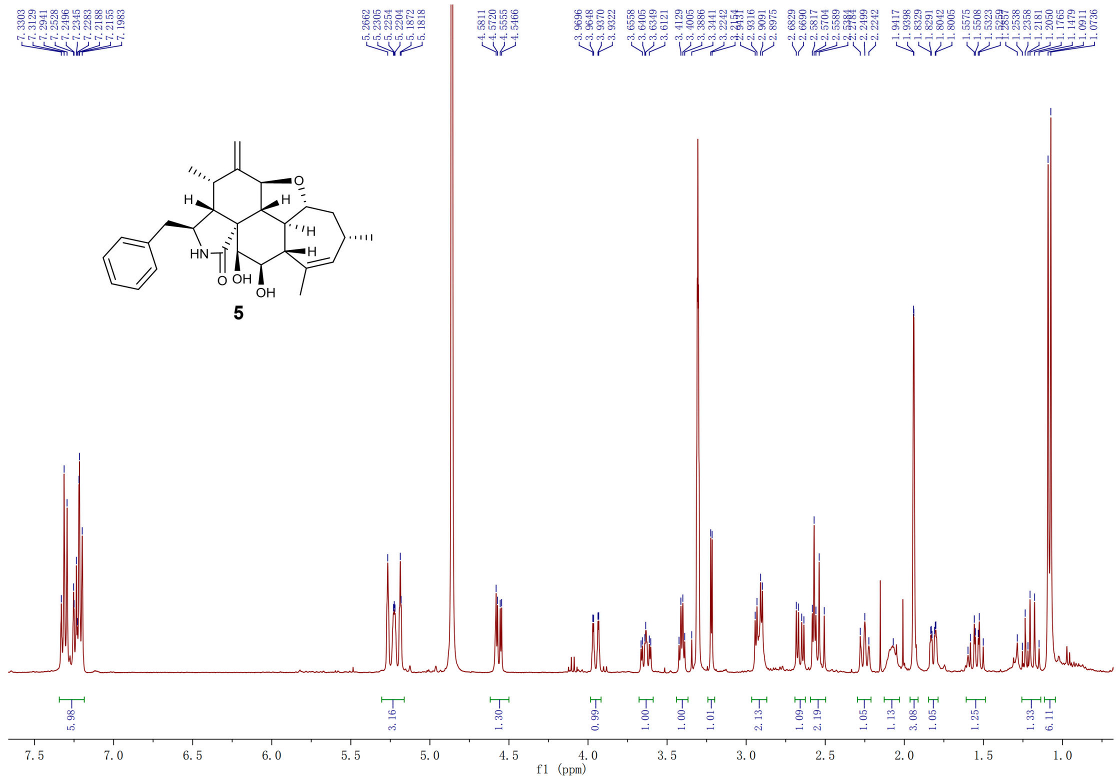

Figure S31. ^{1}H-NMR of phomopchalasin B (**5**)

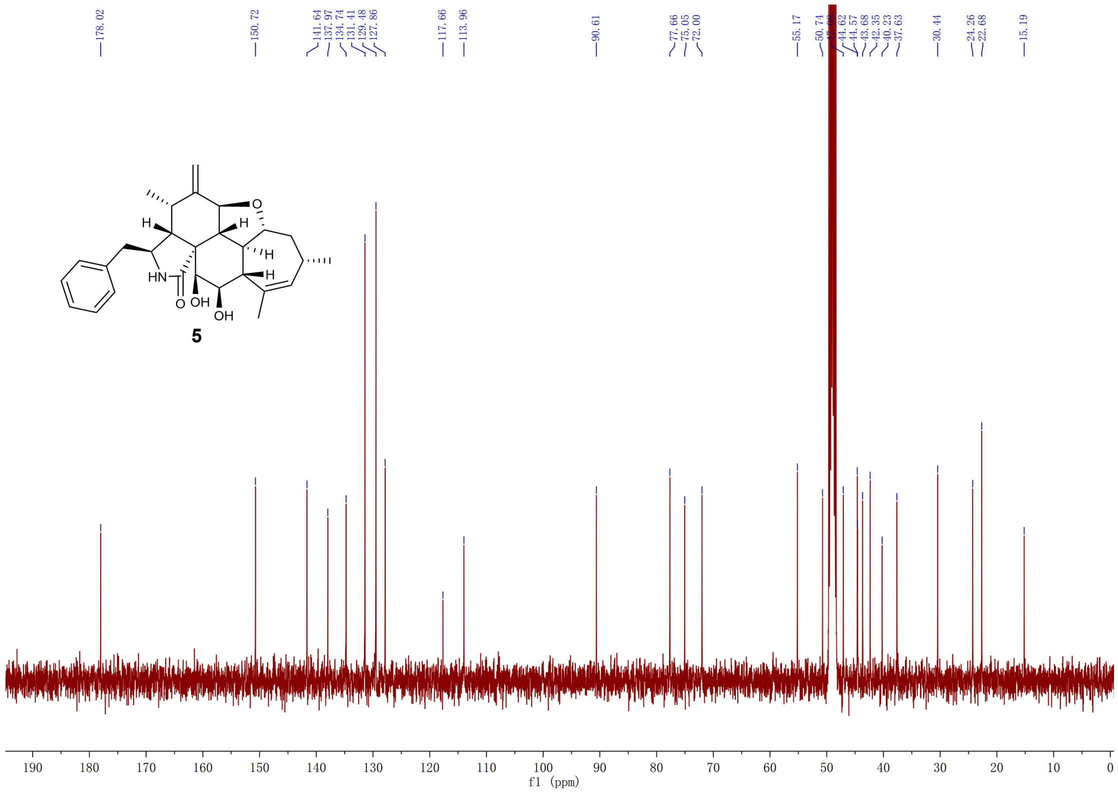

Figure S32. ^{13}C-NMR of phomopchalasin B (**5**)

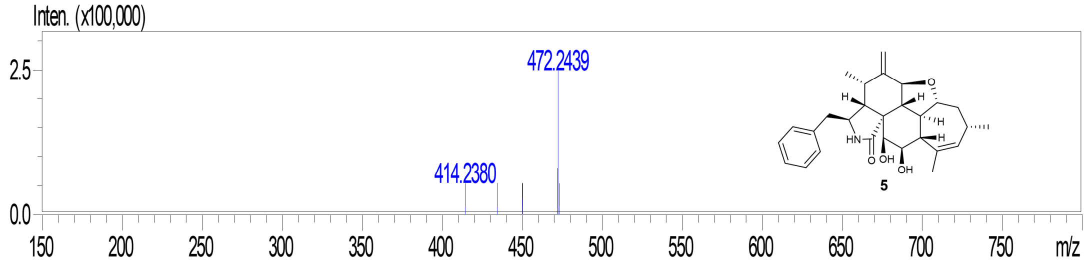

Figure S33. HR-ESI-MS of phomopchalasin B (**5**)

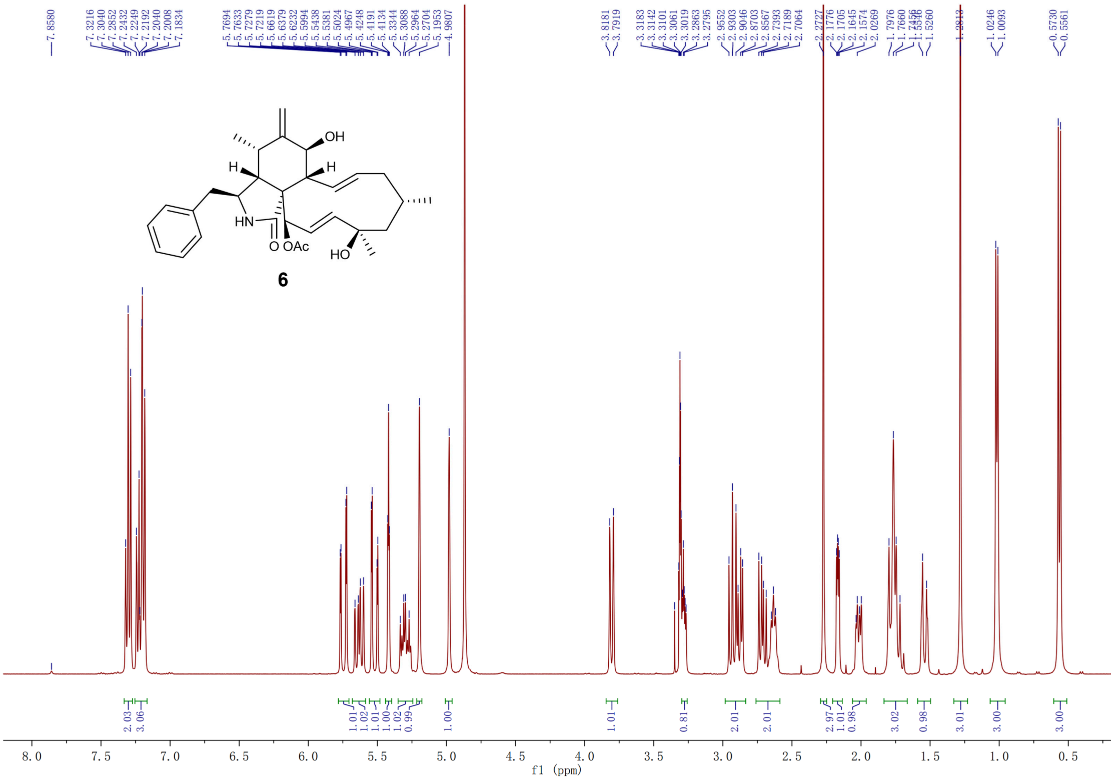

Figure S34. ^{1}H-NMR of cytochalasin H (**6**)

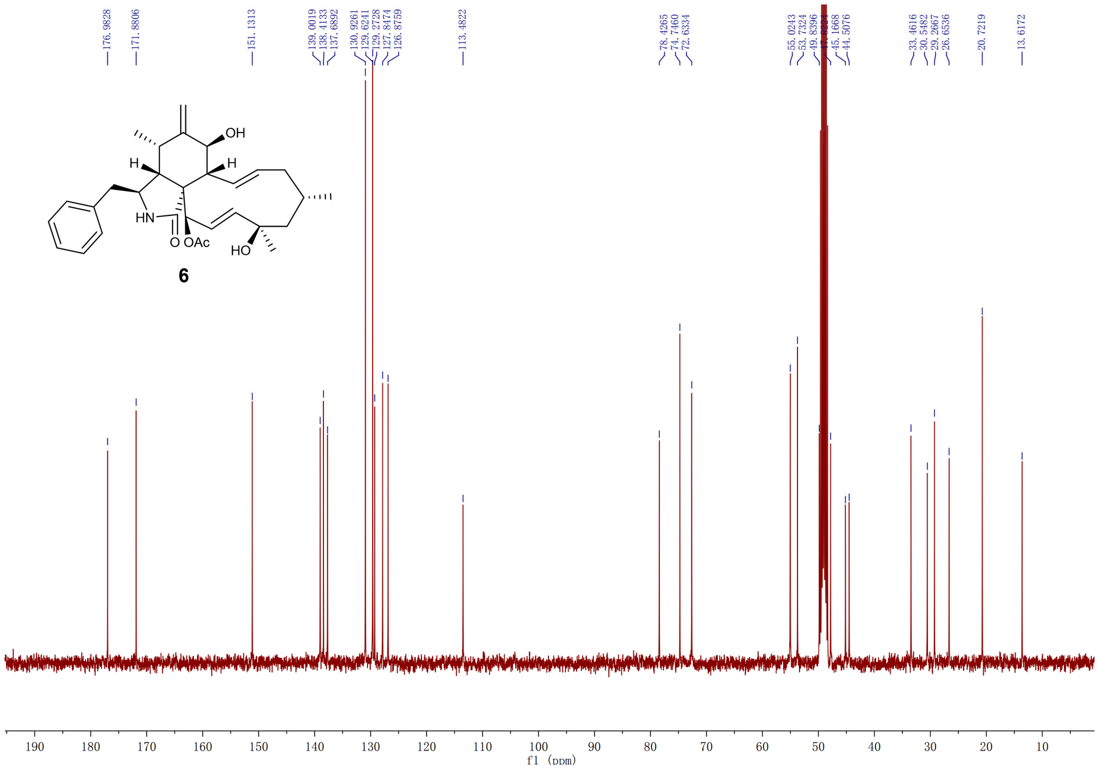

Figure S35. ^{13}C-NMR of cytochalasin H (**6**)

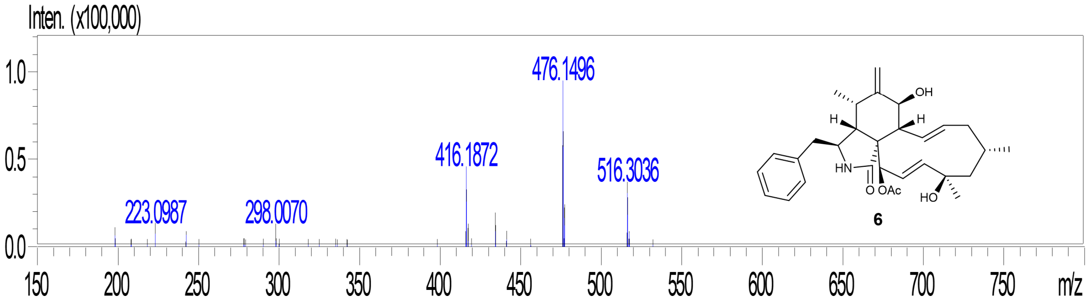

Figure S36. HR-ESI-MS of cytochalasin H (**6**)

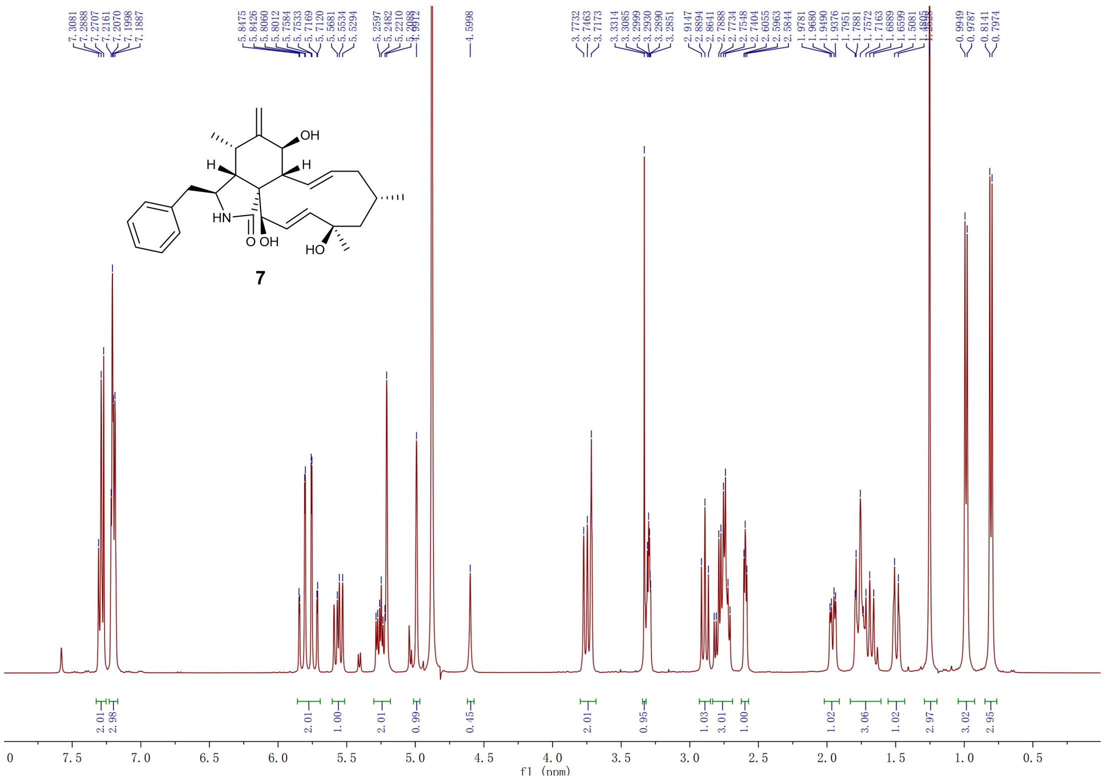

Figure S37. ^{1}H-NMR of cytochalasin J (**7**)

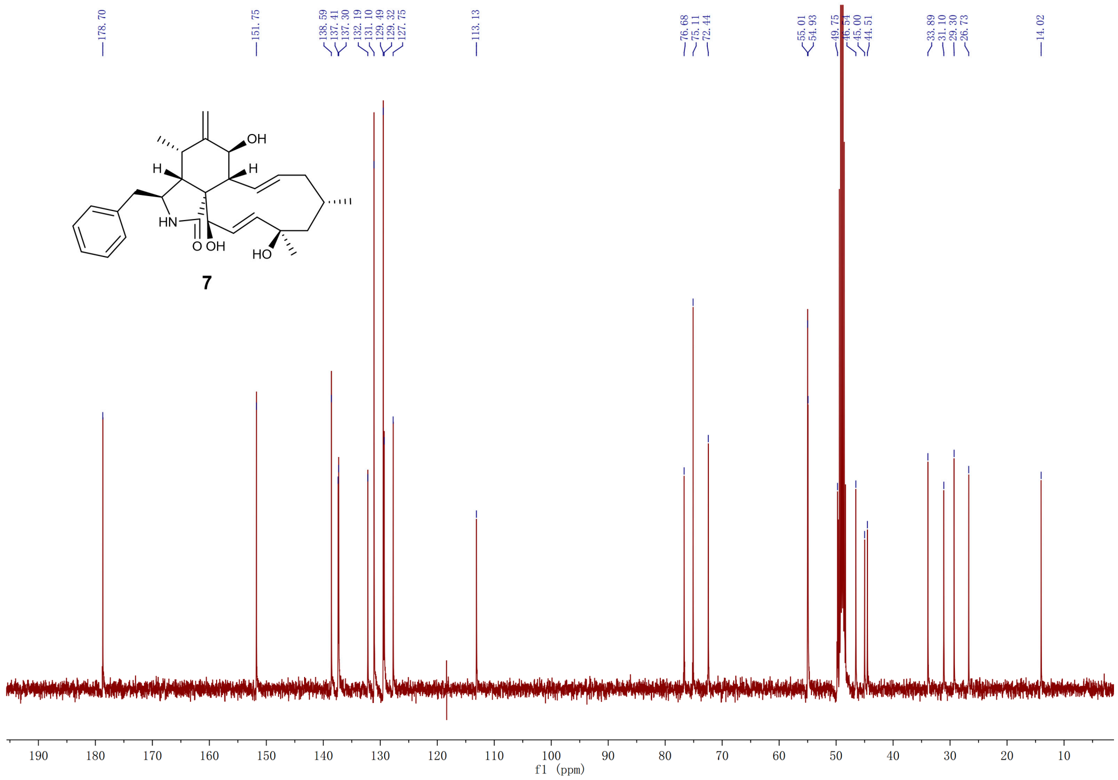

Figure S38. ^{13}C-NMR of cytochalasin J (**7**)

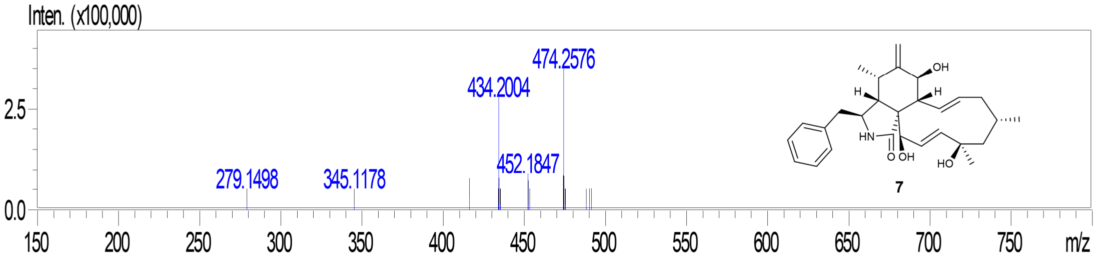

Figure S39. HR-ESI-MS of cytochalasin J (**7**)

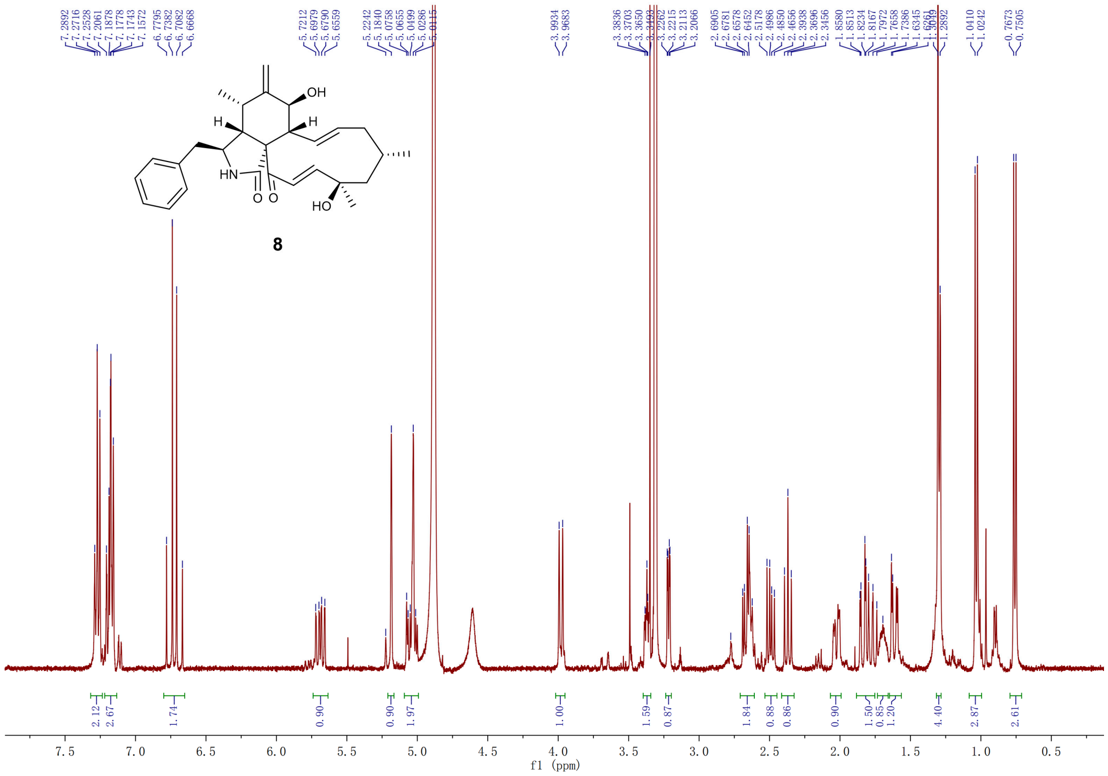

Figure S40. ^{1}H-NMR of fragiformin B (**8**)

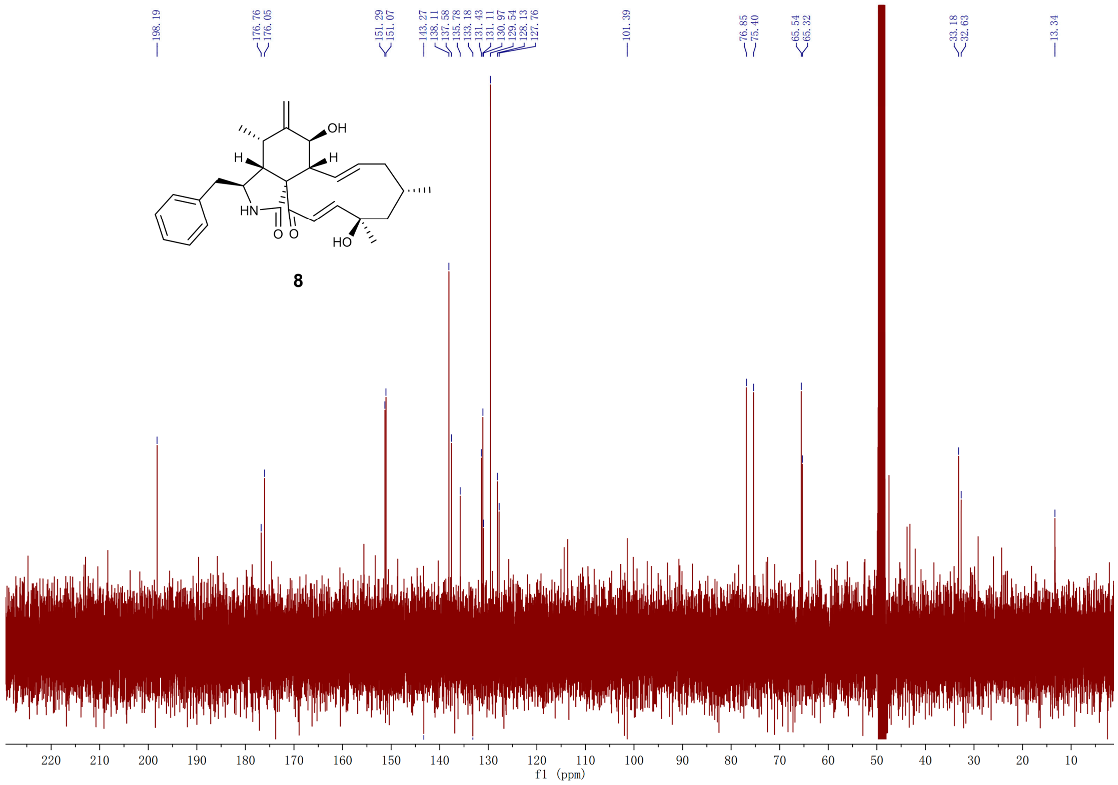

Figure S41. ^{13}C-NMR of fragiformin B (**8**)

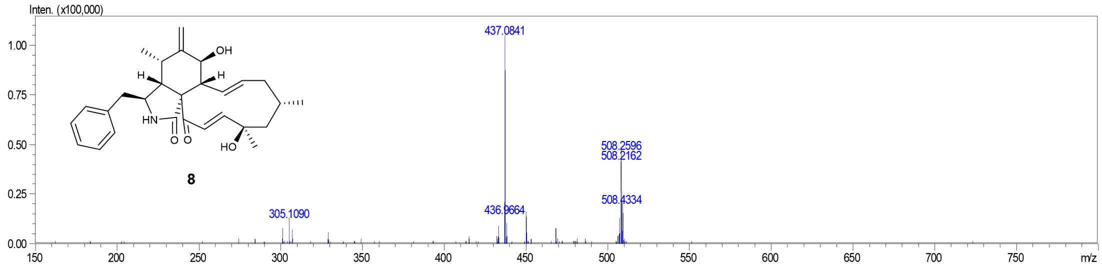

Figure S42. HR-ESI-MS of fragiformin B (**8**)

Figure S43. HPLC spectrum for the purity of tested compounds.

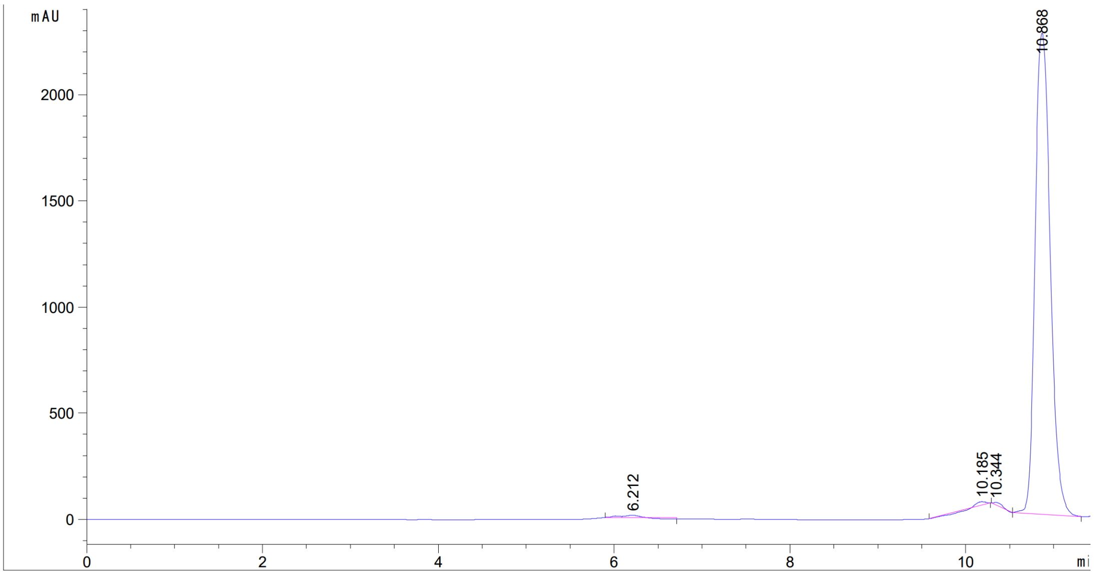

Peak #	Retention time [min]	Peak type	Peak width [min]	Peak area mAU *s	Peak height [mAU]	Peak area% %
1	6.212	BH S	0.0606	36.92249	11.10521	0.1351
2	10.185	BB S	0.0663	36.46985	16.64214	0.1335
3	10.344	BV	0.1171	82.21336	11.66780	0.3008
4	10.868	VB	0.1895	2.71718e4	2265.09033	99.4306
				2.73274e4	2304.50548	

Compound 2

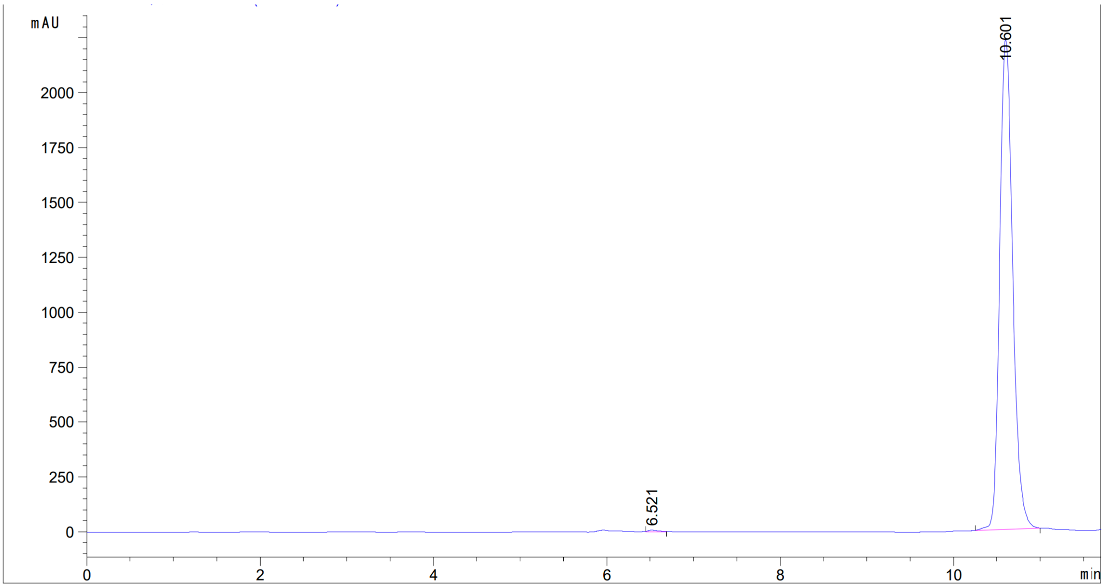

Peak Retention time Peak type Peak width Peak area Peak height Peak area%

Peak	Retention time	Peak type	Peak width	Peak area	Peak height	Peak area%
#	[min]		[min]	mAU *s	[mAU]	%
1	6.521	HH	0.1305	79.65757	8.78695	0.3411
2	10.601	BB S	0.1624	2.32706e4	2231.32593	99.6589
				2.33503e4	2240.11288	

Compound 3

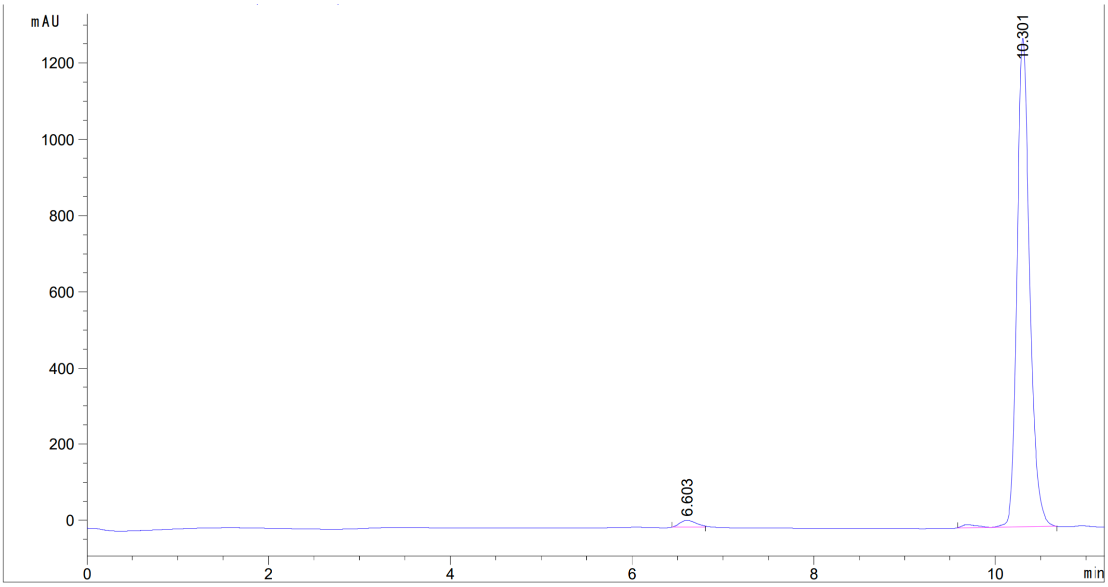

Peak Retention time Peak type Peak width Peak area Peak height Peak area%

#	[min]		[min]	mAU *s	[mAU]	%
1	6.603	HH S	0.1955	229.48477	18.15780	1.7909
2	10.301	BB S	0.1494	1.25846e4	1283.20752	98.2091
				1.28141e4	1301.36532	

Compound 4

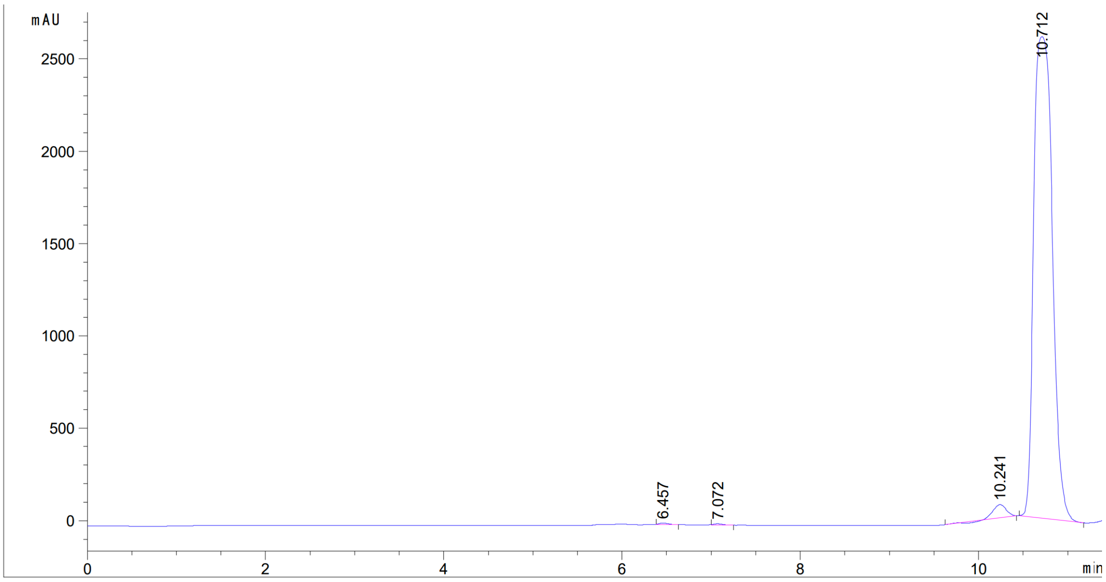

Peak	Retention time	Peak type	Peak width	Peak area	Peak height	Peak area%
#	[min]		[min]	mAU *s	[mAU]	%
1	6.457	HH	0.1029	54.80976	8.34165	0.1422
2	7.072	HB	0.1084	48.62229	6.90464	0.1261
3	10.241	BB S	0.1517	688.45947	71.46004	1.7856
4	10.712	BB	0.2331	3.77639e4	2607.10522	97.9461
				3.85558e4	2693.81156	

Compound 5

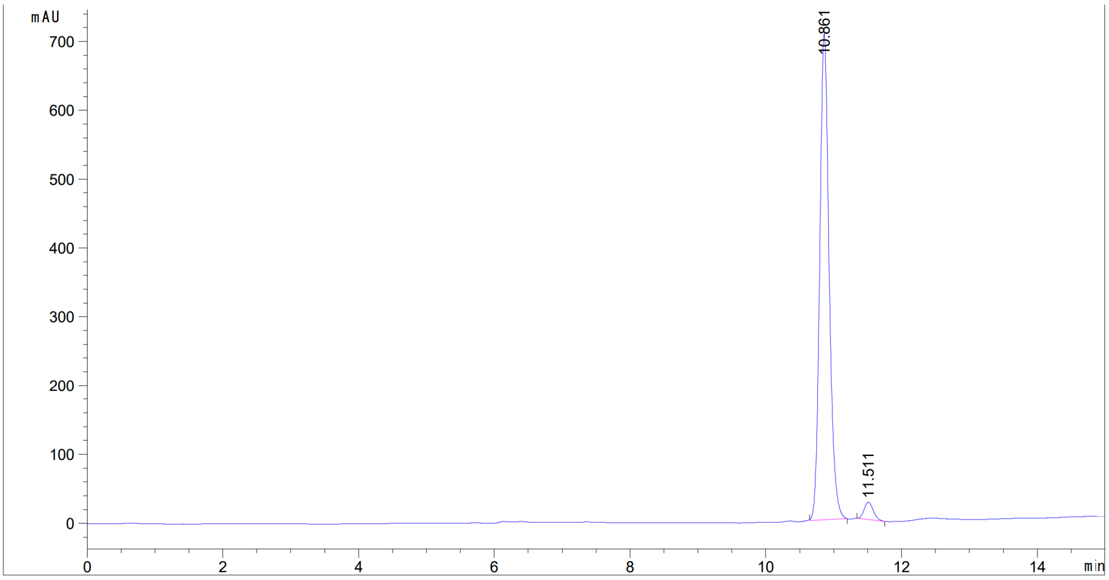

Peak	Retention time	Peak type	Peak width	Peak area	Peak height	Peak area%
#	[min]		[min]	mAU *s	[mAU]	%
1	10.861	BB S	0.1468	6772.04053	706.25555	96.6330
2	11.511	BB	0.1461	235.95837	25.10114	3.3670
				7007.99890	731.35669	

Compound 6

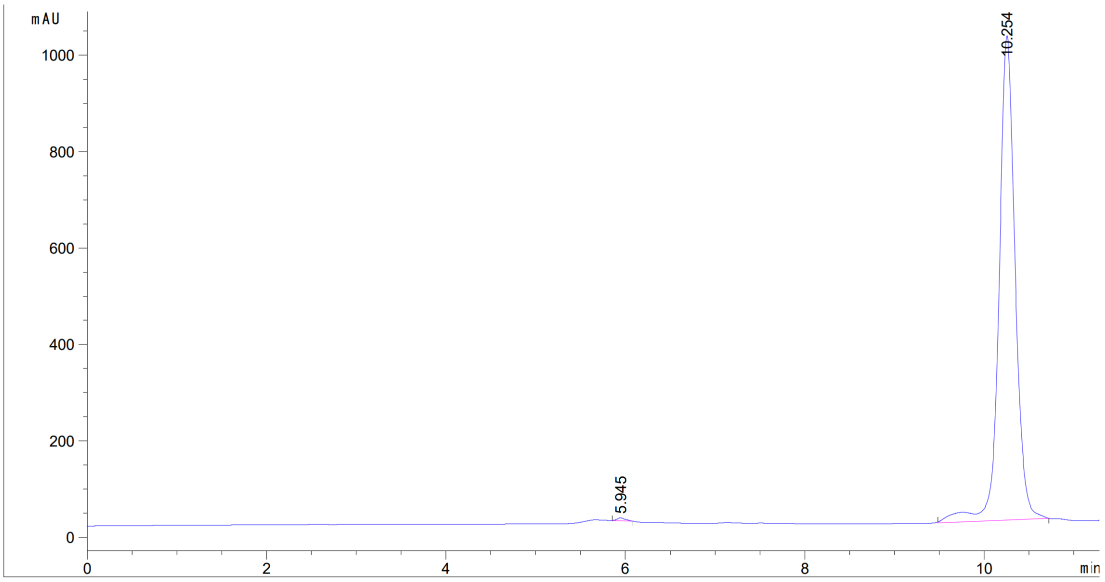

Peak	Retention time	Peak type	Peak width	Peak area	Peak height	Peak area%

#	[min]		[min]	mAU *s	[mAU]	%
1	5.945	BB	0.0916	35.24342	5.65915	0.2787
2	10.254	BB S	0.1887	1.26114e4	1004.30420	99.7213
				1.26466e4	1009.96335	

Compound 7

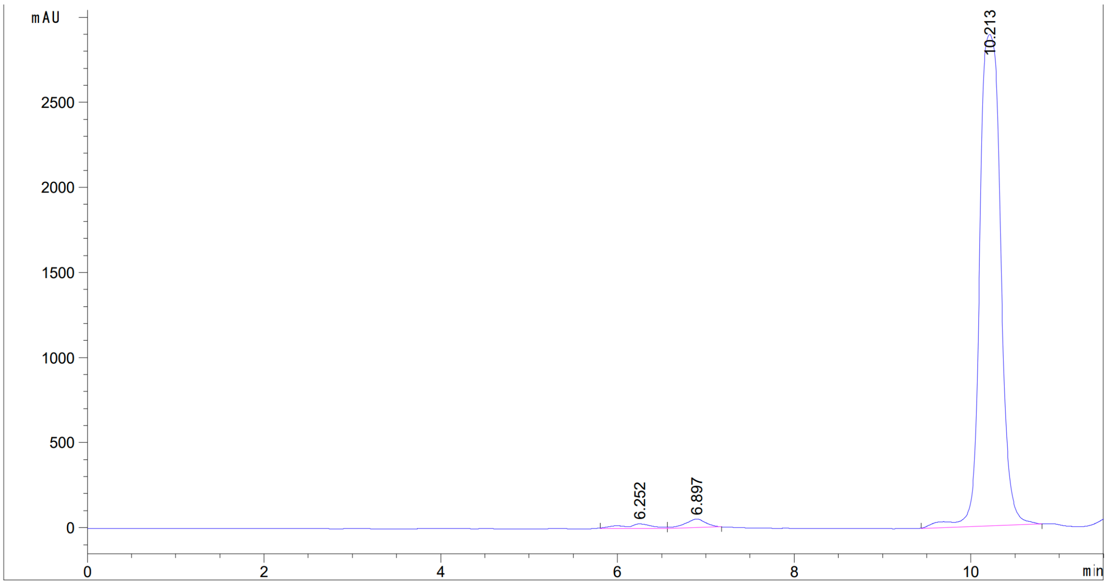

Peak	Retention time	Peak type	Peak width	Peak area	Peak height	Peak area%
#	[min]		[min]	mAU *s	[mAU]	%
1	6.252	BH	0.3366	636.56702	26.40568	1.2993
2	6.897	HB S	0.2333	823.61359	50.67772	1.6811
3	10.213	BB S	0.2604	4.75322e4	2888.53760	97.0196
				4.89924e4	2965.62100	

Figure S44. Effects of **2** on the expression of ConA-induced NFAT protein and analyzed by Western blot

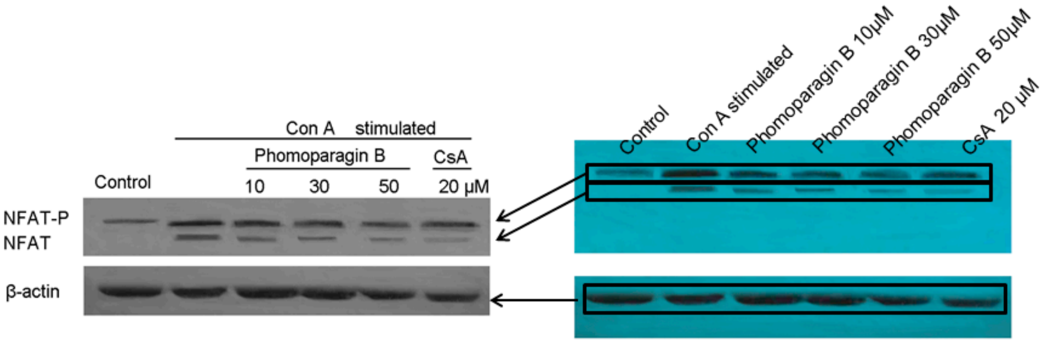
Con A stimulated
Phomoparagin B
CsA
Control
10
30
50
20 μM
NFAT-P
NFAT
β-actin
Control
Con A stimulated
Phomoparagin B 10μM
Phomoparagin B 30μM
Phomoparagin B 50μM
CsA 20 μM
